# Synthesis and Molecular Docking of New N-Acyl Hydrazones-Benzimidazole as hCA I and II Inhibitors

**DOI:** 10.2174/1573406419666221222143530

**Published:** 2023-04-05

**Authors:** Kaan Küçükoğlu, Ulviye Acar Çevik, Hayrunnisa Nadaroglu, Ismail Celik, Ayşen Işık, Hayrani Eren Bostancı, Yusuf Özkay, Zafer Asım Kaplancıklı

**Affiliations:** 1 Department of Pharmaceutical Chemistry, Faculty of Pharmacy, Selçuk University, Konya, Turkey;; 2 Department of Pharmaceutical Chemistry, Faculty of Pharmacy, Anadolu University, Eskişehir, 26470, Turkey;; 3 Department of Food Technology, Erzurum Vocational Training School, Ataturk University, Erzurum, 25240, Turkey;; 4 Department of Pharmaceutical Chemistry, Faculty of Pharmacy, Erciyes University, Kayseri, 38039, Turkey;; 5 Department of Biochemistry, Faculty of Science, Selçuk University, Konya, Turkey;; 6 Department of Biochemistry, Faculty of Pharmacy, Sivas Cumhuriyet University, Sivas, Turkey

**Keywords:** Hydrazone, benzimidazole, carbonic anhydrase I, carbonic anhydrase II, MTT, molecular docking

## Abstract

**Background:**

The carbonic anhydrases (CAs) which are found in most living organisms is a member of the zinc-containing metalloenzyme family. The abnormal levels and activities are frequently associated with various diseases therefore CAs have become an attractive target for the design of inhibitors or activators that can be used in the treatment of those diseases.

**Methods:**

Herein, we have designed and synthesized new benzimidazole-hydrazone derivatives to investigate the effects of these synthesized compounds on CA isoenzymes. Chemical structures of synthesized compounds were confirmed by ^1^H NMR, ^13^C NMR, and HRMS. The synthetic derivatives were screened for their inhibitory potential against carbonic anhydrase I and II by *in vitro* assay.

**Results:**

These compounds have IC_50_ values of 5.156-1.684 µM (hCA I) and 4.334-2.188 µM (hCA II). Inhibition types and Ki values of the compounds were determined. The Ki values of the compounds were 5.44 ± 0.14 µM-0.299 ± 0.01 µM (hCA I) and 3.699 ± 0.041 µM-1.507 ± 0.01 µM (hCA II). The synthetic compounds displayed inhibitory action comparable to that of the clinically utilized reference substance, acetazolamide. According to this, compound **3p** was the most effective molecule with an IC_50_ value of 1.684 µM. Accordingly, the type of inhibition was noncompetitive and the Ki value was 0.299 ± 0.01 µM.

**Conclusion:**

According to the *in vitro* test results, detailed protein-ligand interactions of the compound **3p**, which is more active against hCA I than standard azithromycin (AZM), were analyzed. In addition, the cytotoxic effects of the compounds on the L929 healthy cell line were evaluated.

## 1 INTRODUCTION

Benzimidazole scaffold, which is an N-heterocyclic compound, is the most common in medicinal chemistry. Since heterocycles are used in pharmaceutical, bioinformatics, and drug design, regularly, such scaffolds are often called ‘privileged’ [1]. This heterocyclic is formed by the fusion of a benzene ring to the 4 and 5 positions of an imidazole ring [2]. Some drugs containing benzimidazole ring are marketed like anti-cancer drugs nocodazole and velipralib, anti-protozoal albendazole, phosphodiesterase inhibitor adibenden, analgesic benzitramide, hypotensive diabezole, antiviral maribavir and antihistamine lerisetron [1, 3]. More-over, new benzimidazole analogues continue to be synthe-sized and their biological activities are tested worldwide these biological activities contain antileukemic [4, 5], antimicrobial [6, 7], antiulcer [8, 9], diuretic [10], analgesic [11], calcium channel blocker [12], anti-Alzheimer [13], anti-cancer [14] activities.

The n-Acylhydrazone skeleton which is composed of an amide and an imine group has the ability to interact with hydrogen-bond acceptor and donor sites and could be interacted with a wide range of amino-acid residues. Although there are not many drugs with the N-acyl hydrazone group, [15] it is considered to have potential chemical, therapeutic, biological, and industrial properties [16]. In the search for biological activities of compounds containing N-acyl hydrazone groups, they have been found to exhibit antiprotozoal [17], anti-inflammatory [18, 19], antitrypanosomal [20], antiviral [21], antituberculosis [22], antitumoral [23], antileishmanial [24], and antihypertensive [25] activities.

**Fig. (1):**
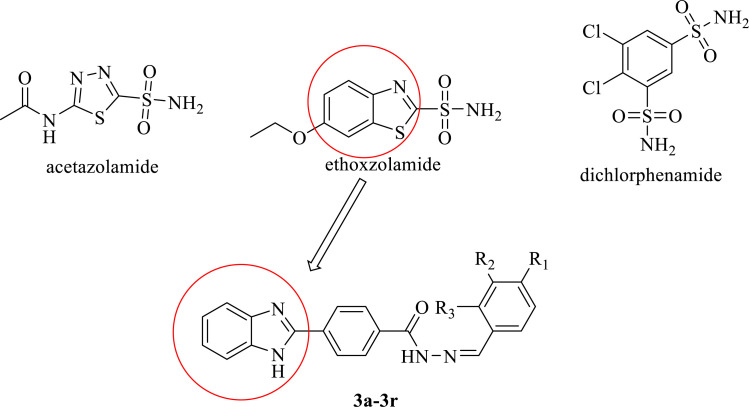
General structure of acetazolamide, ethoxzolamide and dichlorphenamide and synthesized compounds.

The carbonic anhydrases (CAs) which are found in most living organisms are a member of the zinc-containing metalloenzyme family. Their duty is to catalyze the conversion of CO_2_ and H_2_O to HCO_3_^-^ and H^+^ [26, 27]. The type of CAs found in mammals is α-class. Many biochemical processes such as respiration, calcification, pH and bicarbonate homeostasis, signal transduction, lipogenesis, and ureagenesis can be counted among the pivotal physiological events in which these enzymes participate [28, 29]. Moreover, abnormal levels and activities are frequently associated with various diseases so CAs have become an attractive target for the design of inhibitors or activators that can be used in the treatment of those diseases [30-32]. Now, CA inhibitors have been used as anticonvulsants [33], diuretics [34], antiglaucoma [35], or anti-obesity drugs [36]. Among the CAs, CA II, CA IV, and CA XII are CA isoenzymes that are antiglaucoma drug targets [37, 38]. Glaucoma is an optic neuropathy and one of the leading causes of global irreversible blindness in the world [39, 40]. Laser treatment, incisional surgery, and drug therapy are treatment options for glaucoma [41, 42]. Although drug therapy is an essential part of the treatment for glaucoma, systemic side effects such as neurological, psychiatric, and gastrointestinal side effects with currently used drugs create a big problem [43], so there is an urgent need for innovative drug development for glaucoma therapy. Acetazolamide, ethoxzolamide, and dichlorphenamide are clinically important CA inhibitors (Fig. **1**).

Considering above mentioned problems and the logic explained, the design and synthesis of novel *N*-acyl hydrazones containing benzimidazole ring were considered to develop new hCA I (human carbonic anhydrase) and hCA II inhibitor agents. Antimicrobial effects of compounds **3a**, **3b**, **3e**, **3g**, **3k**, **3m**, **3n**, and **3p** have been reported in previous studies [44]. In this study, the effects of carbonic anhydrase were investigated.

## 2 MATERIALS AND METHODS

### 2.1 Chemistry

Synthesis of sodium metabisulfite salt of benzaldehyde derivative:

Ethanol was used to dissolve 5g (0.03 mol) of methyl 4-formyl benzoate. Drop by drop, ethanol-dissolved sodium metabisulfite (6.84 g, 0.036 mol) was added to the benzaldehyde solution. The reaction's components were mixed for an hour at room temperature once the dripping process was finished. It was filtered to obtain the precipitated product.

Synthesis of methyl 4-(1*H*-benzimidazole-2-yl)benzoate (**1**):

The sodium metabisulfite salt of the benzaldehyde derivative (7.09 g, 0.026 mol) was added after the benzene-1,2-diamine (0.022 mol) had been dissolved in dimethylformamide (DMF). The reaction was completed, and the result was precipitated by adding the reaction's components to iced water. The precipitated product was filtered off and crystallized from ethanol.

Synthesis of 4-(1*H*-benzimidazole-2-yl)-benzohydrazide derivatives (**2**):

Compound **1** (0.018 mol), excess of hydrazine hydrate (5 mL), and ethanol (15 mL) were all put into the same vial. Refluxing the mixture for 12 hours. Following the completion of the reaction, the mixture was poured into iced water, the product was filtered.

Synthesis of target compounds **3a**-**3r**:

The compound **2** and appropriate benzaldehyde derivatives in ethanol were refluxed. The precipitated product is filtered off.

#### 2.1.1 N-(1H-benzimidazole-2-yl)-N’-benzylidenebenzo hydrazide (3a)

Yield: 74%. M.p. 279.5^o^C. ^1^H NMR (300 MHz, DMSO-*d_6_*): δ = 7.22-7.27 (2H, m, Aromatic CH), 7.46 (3H, s, Aromatic CH), 7.57 (1H, d, *J=* 6.06 Hz, Aromatic CH), 7.70-7.77 (3H, m, Aromatic CH), 8.11 (2H, d, *J=*7.50 Hz, 1,4-disubstituebenzene), 8.33 (2H, d, *J=*7.59 Hz, 1,4-disubstituebenzene), 8.50 (1H, s, Aromatic CH), 12.00 (1H, s, NH), 13.12 (1H, s, NH). ^13^C NMR (75 MHz, DMSO-*d_6_*): δ(ppm): 110.99, 113.04, 118.58, 121.36, 123.57, 125.72, 127.77, 128.34, 128.68, 130.43, 133.48, 134.68, 135.54, 147.40, 149.56, 150.70, 162.98. [M+H]^+^ calcd for C_21_H_16_N_4_O: 341.1383; found: 341.1397.

#### 2.12 4-((2-(4-(1H-benzimidazole-2-yl)benzoyl)hydrazine ylidene)methyl)benzoic acid (3b)

Yield: 78%. M.p. 338.3^o^C. ^1^H NMR (300 MHz, DMSO-*d_6_*): δ = 7.23-7.26 (2H, m, Aromatic CH), 7.61-7.66 (2H, m, Aromatic CH), 7.87 (2H, d, *J=* 8.37 Hz, Aromatic CH), 8.03 (2H, d, *J=* 7.83 Hz, Aromatic CH), 8.10 (2H, d, *J=* 8.64 Hz, Aromatic CH), 8.33 (2H, d, *J=*8.16 Hz, Aromatic CH), 8.54 (1H, s, Aromatic CH), 12.15 (1H, s, NH). ^13^C NMR (75 MHz, DMSO-*d_6_*): δ(ppm): 125.76, 126.63, 127.83, 127.98, 128.74, 129.18, 129.28, 131.33, 131.41, 132.19, 133.59, 134.38, 138.84, 146.13, 148.33, 150.66, 163.12, 167.38. [M+H]^+^ calcd for C_22_H_16_N_4_O_3_: 385.1285; found: 385.1295.

#### 2.1.3 4-(1H-benzimidazole-2-yl)-N’-(4-(diethylamino) benzylidene)benzohydrazide (3c)

Yield: 72%. M.p. 175.5^o^C. ^1^H NMR (300 MHz, DMSO-*d_6_*): δ = 1.12 (6H, s, -CH_3_), 3.36 (4H, s, -CH_2_), 6.72 (2H, s, Aromatic CH), 7.24 (2H, s, Aromatic CH), 7.54 (3H, s, Aromatic CH), 7.70 (1H, s, Aromatic CH), 8.07 (2H, s, Aromatic CH), 8.30 (3H, s, Aromatic CH), 11.64 (1H, s, NH), 13.10 (1H, s, NH). ^13^C NMR (75 MHz, DMSO-*d_6_*): δ(ppm): 12.92, 44.20, 111.52, 111.99, 119.53, 120.93, 121.74, 122.52, 123.49, 126.75, 128.64, 128.95, 129.35, 133.20, 135.50, 135.63, 149.38, 150.64, 162.38. [M+H]^+^ calcd for C_25_H_25_N_5_O: 412.2140; found: 412.2132.

#### 2.1.4 4-(1H-benzimidazole-2-yl)-N’-(4-isopropyl benzylidene)benzohydrazide (3d)

Yield: 76%. M.p. 280.9^o^C. ^1^H NMR (300 MHz, DMSO-*d_6_*): δ = 1.22 (6H, d, *J=* 6.81 Hz, -CH_3_), 2.89-2.98 (1H, m, -CH), 7.22-7.27 (2H, m, Aromatic CH), 7.35 (2H, d, *J=*7.95 Hz, Aromatic CH), 7.57 (1H, dd, *J_1_*= 6.39 Hz, *J_2_*=1.29 Hz, Aromatic CH), 7.66-7.72 (3H, m, Aromatic CH), 8.09 (2H, d, *J=*8.37 Hz, Aromatic CH), 8.32 (2H, d, *J=*8.43 Hz, Aromatic CH), 8.45 (1H, s, Aromatic CH), 11.93 (1H, s, NH), 13.11 (1H, s, NH). ^13^C NMR (75 MHz, DMSO-d_6_): δ(ppm): 24.15, 33.87, 112.01, 119.58, 122.45, 123.50, 126.80, 127.33, 127.72, 128.78, 132.43, 133.41, 134.65, 135.53, 144.25, 148.49, 150.70, 151.26, 162.88. [M+H]^+^ calcd for C_24_H_22_N_4_O: 383.1863; found: 383.1866.

#### 2.1.5 4-(1H-benzimidazole-2-yl)-N’-(4-chlorobenzylidene) benzohydrazide (3e)

Yield: 76%. M.p. 322.4^o^C. ^1^H NMR (300 MHz, DMSO-*d_6_*): δ = 7.07-7.10 (1H, m, Aromatic CH), 7.20 (1H, d, *J=*8.31 Hz, Aromaric CH), 7.45 (2H, d, *J=*8.28 Hz, Aromaric CH), 7.78-7.81 (3H, m, Aromatic C-H), 7.89-7.90 (3H, m, Aromatic C-H), 7.98-8.00 (3H, m, Aromatic C-H). ^13^C NMR (75 MHz, DMSO-d_6_): δ(ppm):112.68, 114.05, 118.19, 118.43, 119.12, 121.11, 123.14, 128.38, 130.92, 131.80, 132.38, 134.83, 149.62, 151.71, 153.68, 156.20, 193.17. [M+H]^+^ calcd for C_21_H_15_N_4_OCl: 375.1005; found: 375.1007.

#### 2.1.6 4-(1H-benzimidazole-2-yl)-N’-(4-methylthio benzylidene)benzohydrazide (3f)

Yield: 78%. M.p. 290.4^o^C. ^1^H NMR (300 MHz, DMSO-*d_6_*): δ = 2.52 (3H, s, -CH_3_), 7.25-7.27 (2H, m, Aromatic CH), 7.33 (2H, d, *J=*8.37 Hz, 1,4-disubstituebenzene), 7.53-7.58 (1H, m, Aromatic CH), 7.68 (3H, d, *J=*8.37 Hz, Aromatic CH), 8.09 (2H, d, *J=*8.37 Hz, 1,4-disubstituebenzene), 8.32 (2H, d, *J=*8.40 Hz, 1,4-disubstituebenzene), 8.44 (1H, s, Aromatic CH), 11.95 (1H, s, NH), 13.12 (1H, s, NH). ^13^C NMR (75 MHz, DMSO-d_6_): δ(ppm): 13.71, 113.14, 118.56, 121.39, 125.05, 125.71, 127.10, 127.74, 127.89, 129.12, 131.07, 133.33, 133.43, 133.53, 134.58, 144.19, 147.01, 150.70, 162.87. [M+H]^+^ calcd for C_22_H_18_N_4_OS: 387.1263; found: 387.1274.

#### 2.1.7 4-(1H-benzimidazole-2-yl)-N’-(4-trifluoromethyl benzylidene)benzohydrazide (3g)

Yield: 81%. M.p. 335.5^o^C. ^1^H NMR (300 MHz, DMSO-*d_6_*): δ = 7.23-7.25 (2H, m, Aromatic CH), 7.64-7.66 (2H, m, Aromatic CH), 7.83-7.85 (2H, m, Aromatic CH), 7.97-7.99 (2H, m, Aromatic CH), 8.10-8.12 (2H, m, Aromatic CH), 8.32-8.35 (2H, m, Aromatic CH), 8.55 (1H, s, Aromatic CH), 12.16 (1H, s, NH). ^13^C NMR (75 MHz, DMSO-d_6_): δ(ppm): 105.78, 113.20, 114.05, 118.24, 119.31, 120.99, 122.61, 123.41, 133.31, 135.23, 136.28, 144.02, 149.57, 150.78, 151.69, 153.66, 190.35. [M+H]^+^ calcd for C_22_H_15_N_4_OF_3_: 409.1279; found: 409.1271.

#### 2.1.8 N’-([1,1’-biphenyl]-4-ylmethylene)-4-(1H-benzimidazole-2-yl) benzohydrazide (3h)

Yield: 69%. M.p. 307.4^o^C. ^1^H NMR (300 MHz, DMSO-*d_6_*): δ = 7.09 (2H, dd, *J_1_*=3.30 Hz, *J_2_*=9.06 Hz, Aromatic CH), 7.19 (1H, s, Aromatic CH), 7.22 (1H, s, Aromatic CH), 7.36-7.38 (4H, m, Aromatic CH), 7.44 (2H, dd, *J_1_*=1.59 Hz, *J_2_*=8.34 Hz, Aromatic CH), 7.78 (1H, s, Aromatic CH), 7.81 (1H, s, Aromatic CH), 7.87-7.90 (4H, m, Aromatic CH), 7.93 (2H, d, *J=*8.25 Hz, Aromatic CH).^13^C NMR (75 MHz, DMSO-d_6_): δ(ppm): 125.76, 126.11, 126.54, 127.17, 127.78, 127.93, 128.40, 128.50, 128.58, 128.66, 129.28, 129.88, 130.63, 133.47, 133.90, 134.57, 139.79, 146.92, 149.09, 150.66, 162.98. [M+H]^+^ calcd for C_27_H_20_N_4_O: 417.1727; found: 417.1710.

#### 2.1.9 4-(1H-benzimidazole-2-yl)-N’-(4-ethoxybenzylidene) benzohydrazide (3i)

Yield: 79%. M.p. 270.1^o^C. ^1^H NMR (300 MHz, DMSO-*d_6_*): δ = 1.36 (3H, t, *J=*6.90 Hz, CH3), 4.09-4.12 (2H, m, -CH2), 7.13 (3H, d, *J=*8.88 Hz, Aromatic C-H), 7.43-7.47 (2H, m, Aromatic C-H), 7.81 (2H, s, Aromatic CH), 8.04-8.07 (3H, m, Aromatic C-H), 8.15 (3H, d, *J=*8.79 Hz, Aromatic C-H). ^13^C NMR (75 MHz, DMSO-d_6_): δ(ppm): 15.53, 64.36, 115.87, 117.31, 119.49, 121.84, 122.20, 123.70, 129.42, 129.82, 134.75, 136.27, 138.81, 145.57, 150.31, 153.74, 156.50, 161.45, 194.29. [M+H]^+^ calcd for C_23_H_20_N_4_O_2_: 385.1670; found: 385.1659.

#### 2.1.10 4-(1H-benzimidazole-2-yl)-N’-(4-(benzyloxy) benzylidene)benzohydrazide (3j)

Yield: 70%. M.p. 287.0^o^C. ^1^H NMR (300 MHz, DMSO-*d_6_*): δ= 5.18 (2H, s, -CH_3_), 7.12 (2H, d, *J=*8.64 Hz, Aromatic CH), 7.30-7.41 (4H, m, Aromatic CH), 7.47 (2H, d, *J=*7.35 Hz, Aromatic CH), 7.67-7.72 (4H, m, Aromatic CH), 8.11 (2H, d, *J=*8.13 Hz, Aromatic CH), 8.32 (2H, d, *J=*7.95 Hz, Aromatic CH), 8.43 (1H, s, Aromatic CH), 11.91 (1H, s, NH).^13^C NMR (75 MHz, DMSO-d_6_): δ(ppm): 69.81, 115.66, 115.99, 116.25, 122.55, 122.93, 123.66, 127.11, 127.45, 128.28, 128.43, 128.83, 128.96, 129.26, 132.31, 135.36, 137.18, 138.26, 139.32, 148.55, 160.48, 162.66. [M+H]^+^ calcd for C_28_H_22_N_4_O_2_: 447.1803; found: 447.1816.

#### 2.1.11 4-(1H-benzimidazole-2-yl)-N’-(4-methoxy benzylidene)benzohydrazide (3k)

Yield: 71%. M.p. 276.6^o^C. ^1^H NMR (300 MHz, DMSO-*d_6_*): δ = 3.81 (3H, s, -OCH_3_), 7.03 (2H, d, *J=*8.67 Hz, Aromatic CH), 7.28-7.31 (2H, m, Aromatic CH), 7.66-7.69 (4H, m, Aromatic CH), 8.10 (2H, d, *J=*8.43 Hz, 1,4-disubstituebenzene), 8.33 (2H, d, *J=*8.34 Hz, 1,4-disubstituebenzene), 8.44 (1H, s, Aromatic CH), 11.91 (1H, s, NH). ^13^C NMR (75 MHz, DMSO-d_6_): δ(ppm): 55.75, 114.84, 115.43, 115.66, 123.51, 127.06, 127.25, 128.38, 128.82, 129.25, 132.31, 135.17, 138.76, 145.41, 148.45, 150.38, 161.37, 162.68. [M+H]^+^ calcd for C_22_H_18_N_4_O_2_: 371.1504; found: 371.1503.

#### 2.1.12 4-(1H-benzimidazole-2-yl)-N’-(4-fluoro benzylidene)benzohydrazide (3l)

Yield: 68%. M.p. 303.9^o^C. ^1^H NMR (300 MHz, DMSO-*d_6_*): δ = 7.22-7.27 (2H, m, Aromatic CH), 7.30-7.36 (2H, m, Aromatic CH), 7.57 (1H, dd, *J_1_*= 6.54 Hz, *J_2_*=1.29 Hz, Aromatic CH), 7.71 (1H, dd, *J_1_*= 7.02 Hz, *J_2_*=1.50 Hz, Aromatic CH), 7.80-7.85 (2H, m, Aromatic C-H), 8.09 (2H, d, *J=*8.31 Hz, 1,4-disubstituebenzene), 8.32 (2H, d, *J=*8.31 Hz, 1,4-disubstituebenzene), 8.49 (1H, s, Aromatic CH), 12.01 (1H, s, NH), 13.12 (1H, s, NH). ^13^C NMR (75 MHz, DMSO-d_6_): δ(ppm): 112.01, 116.30, 116.59, 119.58, 122.46, 123.51, 126.82, 127.75, 128.81, 129.76, 133.48, 134.53, 135.53, 144.26, 147.32, 150.68, 162.97. [M+H]^+^ calcd for C_21_H_15_N_4_OF: 359.1301; found: 359.1303.

#### 2.1.13 4-(1H-benzimidazole-2-yl)-N’-(4-cyano benzylidene)benzohydrazide (3m)

Yield: 66%. M.p. 297.1^o^C. ^1^H NMR (300 MHz, DMSO-*d_6_*): δ = 7.23-7.26 (2H, m, Aromatic CH), 7.64-7.66 (2H, m, Aromatic CH), 7.94 (4H, s, Aromatic CH), 8.11 (2H, d, *J=*8.01 Hz, 1,4-disubstituebenzene), 8.32 (2H, d, *J=*8.43 Hz, 1,4-disubstituebenzene), 8.53 (1H, s, Aromatic CH), 12.23 (1H, s, NH). ^13^C NMR (75 MHz, DMSO-d_6_): δ(ppm): 112.35, 119.14, 122.06, 124.48, 125.76, 127.09, 127.87, 129.26, 129.74, 132.19, 133.55, 134.38, 138.95, 139.17, 145.38, 147.71, 150.62, 163.33. [M+H]^+^ calcd for C_22_H_15_N_5_O: 366.1335; found: 366.1349.

#### 2.1.14 4-(1H-benzimidazole-2-yl)-N’-(4-nitrobenzylidene) benzohydrazide (3n)

Yield: 78%. M.p. 333.2^o^C. ^1^H NMR (300 MHz, DMSO-*d_6_*): δ = 7.23-7.26 (2H, m, Aromatic CH), 7.64 (1H, m, Aromatic CH), 8.01 (2H, d, *J=*9.03 Hz, 1,4-disubstituebenzene), 8.11 (2H, d, *J=*8.31 Hz, 1,4-disubstituebenzene), 8.30-8.35 (5H, m, Aromatic CH), 8.58 (1H, s, Aromatic CH), 12.29 (1H, s, NH). ^13^C NMR (75 MHz, DMSO-d_6_): δ(ppm): 123.48, 125.72, 127.43, 127.92, 129.44, 130.14, 133.61, 134.16, 136.11, 136.23, 140.99, 144.81, 147.05, 148.32, 150.62, 155.13, 163.25.

#### 2.1.15 4-(1H-benzimidazole-2-yl)-N’-(3-nitrobenzylidene) benzohydrazide (3o)

Yield: 73%. M.p. 310.1^o^C. ^1^H NMR (300 MHz, DMSO-*d_6_*): δ = 7.23-7.26 (2H, m, Aromatic CH), 7.64 (2H, s, Aromatic CH), 7.74-7.79 (1H, m, Aromatic CH), 8.11 (2H, d, *J=*8.16 Hz, 1,4-disubstituebenzene), 8.17 (1H, d, *J=*7.50 Hz, Aromatic CH), 8.27 (1H, d, *J=*7.92 Hz, Aromatic CH), 8.33 (2H, d, *J=*8.19 Hz, 1,4-disubstituebenzene), 8.59 (2H, s, Aromatic CH), 12.26 (1H, s, NH). ^13^C NMR (75 MHz, DMSO-d_6_): δ(ppm): 121.43, 122.83, 122.99, 123.40, 124.83, 126.85, 127.93, 128.58, 128.92, 130.98, 133.65, 133.93, 134.23, 136.59, 139.24, 146.01, 148.69, 150.63, 163.23.

#### 2.1.16 4-(1H-benzimidazole-2-yl)-N’-(4-methylbenzyli-dene) benzohydrazide (3p)

Yield: 72%. M.p. 325.8^o^C. ^1^H NMR (300 MHz, DMSO-*d_6_*): δ = 2.36 (3H, s, -CH_3_), 7.22-7.25 (2H, m, Aromatic CH), 7.29 (2H, d, *J=*7.98 Hz, Aromatic CH), 7.57 (1H, dd, *J_1_*= 6.48 Hz, *J_2_*=1.35 Hz, Aromatic CH), 7.65 (2H, d, *J=*7.95 Hz, Aromatic CH), 7.70 (1H, dd, *J_1_*= 7.23 Hz, *J_2_*=1.98 Hz, Aromatic CH), 8.08 (2H, d, *J=*8.34 Hz, 1,4-disubstituebenzene), 8.31 (2H, d, *J=*8.43 Hz, 1,4-disubstituebenzene), 8.45 (1H, s, Aromatic CH), 11.92 (1H, s, NH), 13.11 (1H, s, NH). ^13^C NMR (75 MHz, DMSO-d_6_): δ(ppm): 15.54, 115.35, 116.29, 117.31, 120.72, 121.04, 122.86, 128.36, 128.97, 130.92, 132.37, 133.52, 134.89, 149.59, 153.79, 156.18, 160.15, 193.17. [M+H]^+^ calcd for C_22_H_18_N_4_O: 355.1545; found: 355.1553.

#### 2.1.17 4-(1H-benzimidazole-2-yl)-N’-(2-methylbenzyli-dene) benzohydrazide (3r)

Yield: 81%. M.p. 282.3^o^C. ^1^H NMR (300 MHz, DMSO-*d_6_*): δ = 2.36 (3H, s, -CH_3_), 7.20-7.31 (4H, m, Aromatic CH), 7.57 (1H, dd, *J_1_*= 6.48 Hz, *J_2_*=1.35 Hz, Aromatic CH), 7.64-7.71 (3H, m, Aromatic CH), 8.08 (2H, d, *J=*8.34 Hz, 1,4-disubstituebenzene), 8.31 (2H, d, *J=*8.43 Hz, 1,4-disubstituebenzene), 8.45 (1H, s, Aromatic CH), 11.92 (1H, s, NH), 13.11 (1H, s, NH). ^13^C NMR (75 MHz, DMSO-d_6_): δ(ppm): 20.35, 123.79, 125.63, 125.72, 125.84, 127.26, 127.63, 127.72, 127.86, 127.94, 132.78, 133.37, 133.46, 134.56, 137.46, 146.07, 147.64, 148.14, 150.99, 163.73. [M+H]^+^ calcd for C_22_H_18_N_4_O: 355.1544; found: 355.1553.

### 2.2 hCA Inhibition Assay

Purification of hCA I and hCA II by affinity chromatography was performed as described in the previous work [45-47].

### 2.3 Hydratase Activity

The Wilbur-Anderson method, as modified by Wilber *et al*. [47, 48], was used to calculate CA activity. With the help of a bromothymol blue indicator and a measurement of the passing time, the pH changes were calculated using this approach, which causes the hydration of CO_2_ to release H^+^ ions. The equation (to-tc/tc), where to and tc are the times for pH change of the enzymatic and nonenzymatic processes, respectively, was used to compute the enzyme unit (EU).

### 2.4 Inhibition Assay

Investigated were the inhibitory effects of compounds **3a-3r** and AZM on the hydratase activity of the isoenzymes hCA I and hCA II. While keeping the concentration of the substrate constant, IC_50_ values for the various compounds were computed. Enzyme activities in the absence of inhibitors in the medium were taken as 100% activity. By assessing the hydratase activity in the presence of various inhibitor concentrations, the activity % values of enzymes were calculated. Utilizing graphs of activity %-[I] for each inhibitor, the IC_50_ value was determined [48-50]. The Cheng-Prusoff equation was used to derive inhibition constants using the nonlinear least squares method [51-53].

### 2.5 Cytotoxicity Assay

The effect of the compounds between **3a-3r** on the viability of the L929 cell line was analyzed by MTT assay as described in the previous work [54-56]. Cell L929 was obtained from ATCC and multiplied in Hepokur Lab.

### 2.6 Molecular Docking

All stages of molecular docking studies protein preparation, ligand preparation, active site grid generation, and ligand docking were carried out using Schrödinger Suite software 2021.1 version. 3D structures of target proteins hCA I (PDB ID: 3W6H, Resolution: 2.96 Å) [54] and hCA II (PDB ID: 4G0C, Resolution: 2.00 Å) [55] were obtained from the protein data bank (PDB). Target proteins were created using the 'Protein Preparation Wizard' default parameters after water and other heteroatoms other from Zn^2+^ were eliminated. The “LigPrep” tool was used to create the 3D minimizing structures of the compounds **3a-3r** at pH:7.2 With the “Receptor Grid Generation” module based on the cocrystal ligand AZM, the active site coordinates file for both target proteins hCA I (x: 33.6, y: -1.33, z: 9.01) and hCA II (x: -4.98, y: 3.81, z: 14.7) were generated as 20*20*20 Å3. To validate the molecular docking work, re-docking was performed with Glide SP and the cocrystal ligand AZM [56]. Then, molecular docking of all compounds was performed with Glide SP ligand docking tools. 2D protein-ligand interactions diagram ‘Ligand Interaction' module and 3D interactions were made in Maestro v12.8 interface.

## 3 RESULTS AND DISCUSSION

### 3.1 Chemistry

**Scheme 1:**
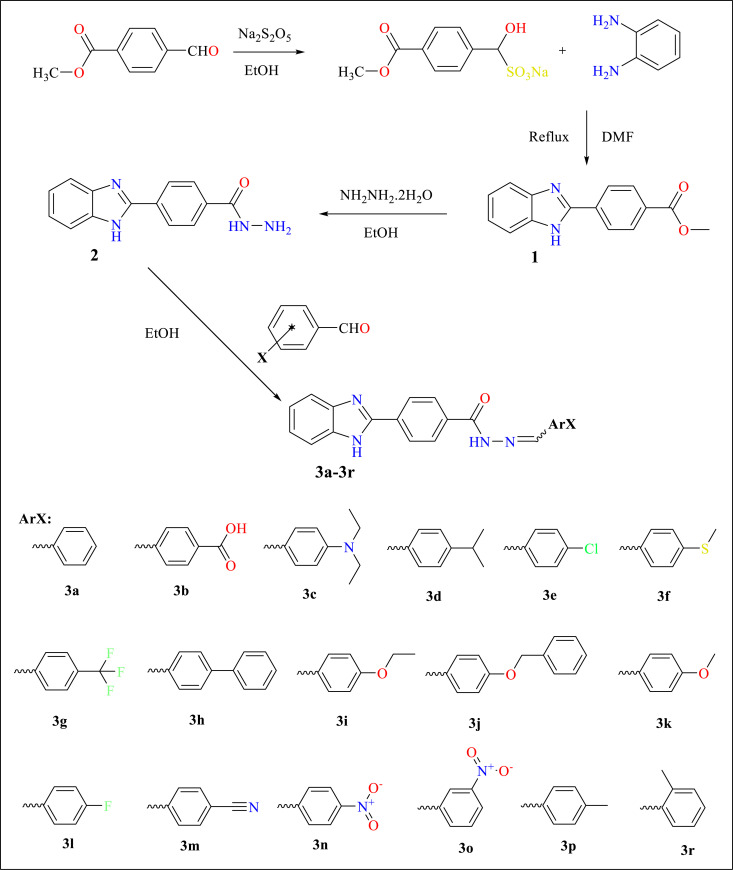
General procedure for synthesis of the final compounds **3a-3r.**

As shown in Scheme **1**, the target molecules were synthesized in four steps. First, the aldehyde part of the methyl 4-formylbenzoate compound was treated with sodium metabisulfite in ethanol to obtain the sodium disulfide addition product of the aldehyde. In the second step, as a result of the condensation reaction of benzaldehyde sodium metabisulfite product and o-phenylenediamine under reflux and methyl 4-(1*H*- benzimidazol-2-yl)benzoate (**1**) was obtained. In the next step, compound **1** was treated with hydrazine hydrate in ethanol to obtain the 4-(1*H*-benzimidazol-2-yl)benzohydra-zide (**2**). The hydrazide derivative compound (**2**) and appropriate benzaldehyde derivatives in ethanol were refluxed and obtained target compounds **3a-3r**. The structures of the target compounds were confirmed via ^1^H NMR, ^13^C NMR, and HRMS spectroscopy.

Methyl protons from the -C_2_H_5_ protons of compound **3c** were observed at 1.12 ppm, and -CH_2_ protons at 3.36 ppm. Methyl protons from the -isopropyl group of compound **3d** were observed at 1.22 ppm as duplet, and -CH protons at 2.89-2.98 ppm as a multiplet. The protons of the -CH_3_ group of thiomethyl substituent of compound **3f** were observed at 2.52 ppm as a singlet. The methoxy group in the 4^th^ position of the phenyl ring of compound **3k** was observed as a singlet at 3.81 ppm. The signals belonging to aromatic protons were found at 6.72-8.59 ppm. The ^13^C NMR spectra showed peaks around 165 ppm due to the carbonyl group (C=O). All of the derivatives' ^13^C NMR spectra revealed carbon values in the expected locations, and the HRMS analysis supported the mass with the target compounds' estimated values.

### 3.2 *In vitro* hCA Activity

The compounds **3a-3r** were tested for their *in vitro* inhibitory effects on hCA I and hCA II isoenzymes and the results are presented in (Fig. **1**). In this work, acetazolamide was used as a reference compound. Compounds **3a**-**3r** showed hCA I inhibitory activity with IC_50_ values ranging from 1.684 to 5.156 µM. In these series, compounds **3p**, **3d**, **3j**, and **3c** were the only compounds that showed better inhibitory activity against hCA I isoenzyme than the reference compound AZM with the IC_50_ values of 1.684, 1.87, 1.952, and 2.093 µM, respectively.

These series of N-acyl hydrazones exhibited inhibitory effects on hCA II isoenzyme with IC_50_ values ranging from 2.188 to 4.334 µM and none of the compounds had better inhibitory effects compared to AZM (IC_50_ = 1.17 µM). Compounds **3a**, **3i**, **3k**, and **3n** showed more inhibitory activity on hCA II than hCA I, but the other tested compounds showed more inhibitory effects on hCA I than hCA II, except compound **3l**. 4-Fluoro derivative **3l** indicated fairly close inhibitory properties on hCA I and hCA II isozymes with the IC_50_ values of 2.716 and 2.738 µM, respectively.

**Fig. (2):**
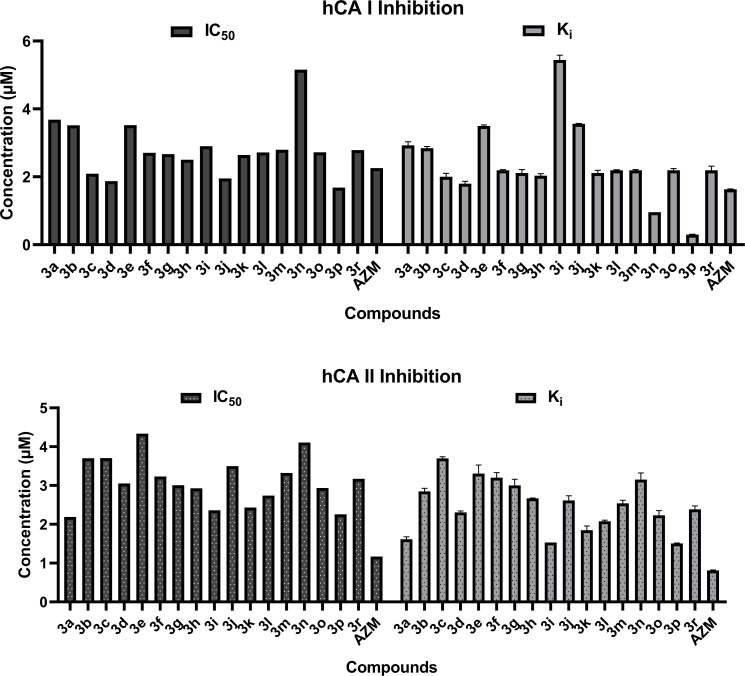
IC_50_ and K_i_ values (µM) of the new N-acyl hydrazones compounds **3a-3r** and standard acetazolamide (AZM) with hCA I and hCA II.

The inhibition constants (Ki) of compounds **3a-3r** for hCA I were established between 0.299 and 5.44 µM and are shown in (Fig. **2**). In these compounds, 4-methylphenyl derivative **3p** (Ki = 0.299 µM) and a 4-nitrophenyl derivative **3n** (Ki = 0.956 µM) had lower Ki values than that of AZM (Ki = 1.63 µM) representing their inhibitory activity against hCA I isoenzyme. On the other hand, all the tested compounds had greater Ki values ranging between 1.507 and 3.699 µM compared to AZM (Ki = 0.812 µM) on hCA II isoenzyme.

The IC_50_ and Ki values obtained from activity tests revealed that all the compounds tested towards hCA I and II isoforms showed noncompetitive type enzyme inhibition. According to *in vitro* assay, compounds **3p**, **3d**, **3j**, and **3c** indicate significant hCA I inhibitory activity, even though the sulfonamide group, which is an important pharmacophore for hCA inhibitory activity, is not available in their structures. 4-methyl substituents on the phenyl ring at the 4^th^ position were found a generally useful modification in increasing hCA I inhibitory activity. In compound **3r**, where the methyl group is in the 2^nd^ position, it is seen that the activity decreases significantly. It was determined that compound **3d**, which has an isopropyl structure instead of methyl in the 4^th^ position, also showed similar activity to compound **3p**.

### 3.3 Cytotoxicity Assay

**Fig. (3):**
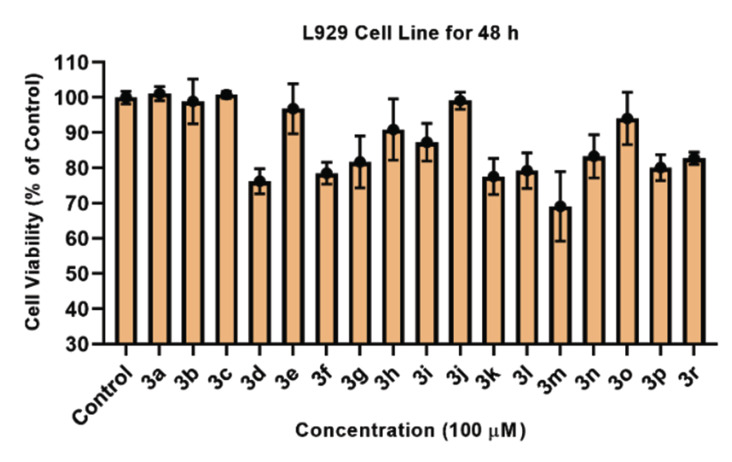
Cell Viability (%) of L929 fibroblast cell line against compounds (**3a-3r**) for 48h.

The cytotoxic bioactivity of synthetic compounds was assessed *in vitro* using the MTT test against the L929 cell line for preliminary screening. The target compounds were administered to the fibroblast cells at a constant dose of 100 M to assess their cytotoxic potential. After the cells had been treated for 48 hours, cell viability percentages were calculated. Preliminary anti ınflammatory effect results of compounds **3a-3r** against L929 fibroblast are presented in (Fig. **3**). As a result of the maximum dose applied, all compounds showed 75% more viability. So as a result of this IC_50_ values of compounds were not calculated because they were greater than 100 µM.

### 3.4 Molecular Docking

A molecular docking study was carried out to detect and show the interaction of the synthesized compounds with hCA I and II. In order to compare the synthesized compounds and to validate the molecular docking study, self-docking was performed on the cocrystal ligand AZM, which is located in the hCA I (PDB ID: 3W6H) [54] and II (PDB ID: 4G0C) [55] crystal three-dimensional structures. The RMSD value of AZM between the natural interaction pose and docking pose was measured as 1.36 Å for 3W6H and 0.17 Å for 4G0C. As given in (Table **1**), the compounds gave docking scores between -3.369 and -6.713 kcal/mol, and glide emodel docking scores of -38.264 and -71.940 kcal/mol against the hCA I enzyme. Glide docking scores between -3.121 and -6.547 kcal/mol, and glide emodel binding energies of -39.199 and -70.942 kcal/mol against the CA II enzyme were formed. The standard compound and cocrystal ligand AZM gave -7.893 kcal/mol Glide binding energy and -70.865 Glide emodel binding energy to hCA I enzyme, while they gave -7.097 kcal/mol and -67.269 kcal/mol binding energies to hCA II enzyme, respectively.

**Fig. (4):**
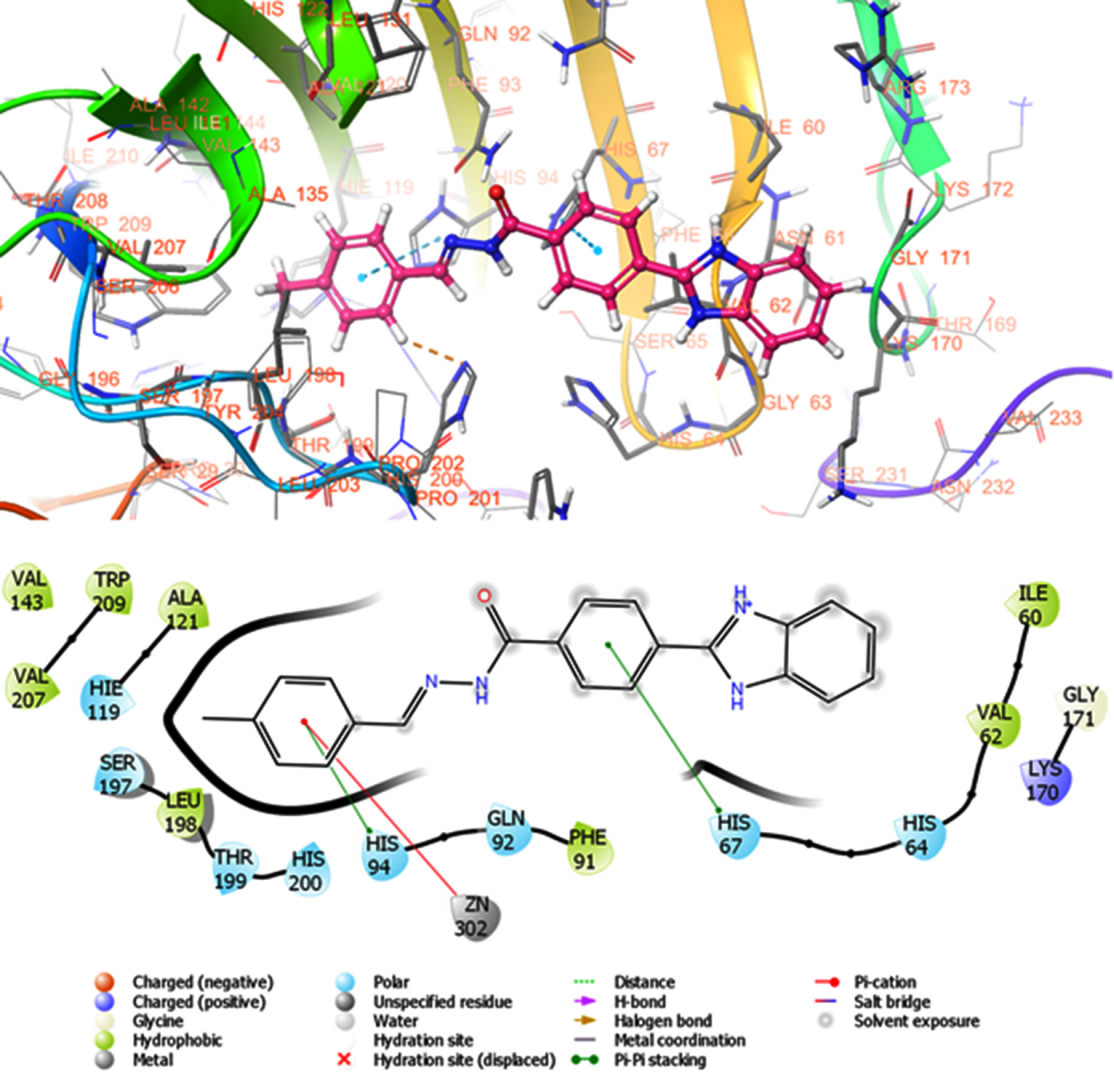
Binding poses and protein-ligand interaction diagram of most active compounds **3p** against hCA I obtained from Glide SP molecular docking.

According to the *in vitro* test results, detailed protein-ligand interactions of the compound **3p**, which is more active against hCA I than standard AZM, were analyzed. As given in (Fig. **4**), compound **3p** formed one H bond with His67 (2.22 Å), π-π stacking interactions with His67 (4.83 Å) and His94 (4.75 Å), and pi-cation interactions with Zn^2+^ (4.25 Å). In addition, hydrophobic interactions with Ile60, Val62, Phe91, Ala121, Val143, Leu198, Val207 and Trp209, positively charged interactions with Lys170, polar van der Waals interactions with His64, His67, Gln92, His94, Hie119, Ser197, Thr199, and His200. The other active compound 3d, two H bonds with Trp5 (2.66 Å) and His67 (2.49 Å), π-π stacking with His64 (5.01 Å) and His200 (4.86 Å and 5.08 Å), and residues His94 (5.86 Å) and Lys170. (4.49 Å) with π-cation interactions. Compound 3j formed π-π stacking with His94 (4.86 Å), hydrophobic interactions with Leu198, Pro202, Tyr204, Ala135, Tyr20 and Ala121, and polar van der Waals interactions with His64, Gln92, His94, His200 and Thr199.

## CONCLUSION

In this paper, new N-acyl hydrazones containing benzimidazole ring compounds **3a-3r**, were synthesized and evaluated for their ability to inhibit hCA I and hCA II isoforms. Despite the absence of a sulfonamide group, which is an important functional group for carbonic anhydrase enzyme inhibitory activity, in the structures of these compounds, it is attractive that enzyme inhibition activity is observed. In this study, we have shown that the sulfonamide group is not a must for carbonic anhydrase inhibiting activity. Among them, a 4-methylphenyl derivative **3p** was the strongest compound on hCA I isozyme according to IC_50_ and Ki values. According to the result, we showed that built-in guanidine or =NNH-CO moieties may also play a crucial role in the process of inhibition. These compounds may serve as a promising candidate for further studies to develop new hCA inhibitory compounds. According to the *in vitro* test results, detailed protein-ligand interactions of the compound **3p**, which is more active against hCA I than standard AZM, were analyzed by molecular docking.

## Figures and Tables

**Table 1:** Molecular docking binding energies (kcal/mol) of compounds 3a-3r and reference acetazolamide (AZM) with hCA I and hCA II.

**-**	**hCA I**		**hCA II**	
**Comp.**	**Glide gscore**	**Glide ** **emodel**	**Glide gscore**	**Glide ** **emodel**
**3a**	-3.708	-44.053	-3.819	-39.199
**3b**	-6.713	-71.940	-6.547	-70.942
**3c**	-3.657	-50.887	-3.022	-44.450
**3d**	-4.536	-38.264	-3.482	-45.824
**3e**	-3.955	-39.943	-3.250	-48.468
**3f**	-3.369	-45.741	-3.233	-50.193
**3g**	-3.771	-47.527	-3.137	-48.827
**3h**	-3.878	-50.963	-3.453	-53.708
**3i**	-3.243	-45.768	-3.121	-47.667
**3j**	-3.692	-50.278	-3.473	-53.466
**3k**	-3.887	-46.158	-3.199	-46.962
**3l**	-3.887	-46.158	-3.199	-46.962
**3m**	-4.926	-47.682	-3.823	-44.852
**3n**	-4.224	-51.377	-3.103	-50.201
**3o**	-3.969	-50.551	-3.301	-51.546
**3p**	-4.430	-44.340	-4.213	-47.914
**3r**	-4.295	-49.790	-3.514	-45.492
**AZM**	-7.893	-70.865	-7.097	-67.269

## Data Availability

The data that support the findings of this study are available within the article.

## LIST OF ABBREVIATIONS

CAs: Carbonic Anhydrases
DMF: Dimethylformamide
EU: Enzyme Unit
