# Supplementary material for: Synthesis and Molecular Docking of New N-Acyl Hydrazones-Benzimidazole as hCA I and II Inhibitors
Source: Med Chem. 2023 Apr 5;19(5):485–94. doi: 10.2174/1573406419666221222143530 (PMC10258912; doi:10.2174/1573406419666221222143530)
Supplement: Supplementary file 1 — Supplementary material is available on the publisher's website along with the published article. [file MC-19-485_SD1.pdf]

Supplementary Materials

Synthesis and Molecular Docking of New N-Acyl Hydrazones-Benzimidazole as hCA I and II Inhibitors

Kaan Küçükoğlu*[1], Ulviye Acar Çevik[2], Hayrunnisa Nadaroglu[3], İsmail Çelik[4], Ayşen Işık[5], Hayrani Eren Bostanlı[6], Yusuf Özkay[2], Zafer Asım Kaplancıklı[2]

[1] Department of Pharmaceutical Chemistry, Faculty of Pharmacy, Selçuk University, Konya 26470, Turkey.
[2] Department of Pharmaceutical Chemistry, Faculty of Pharmacy, Anadolu University, Eskişehir 26470, Turkey.
[3] Department of Food Technology, ErzurumVocational Training School, Ataturk University, 25240 Erzurum, Turkey
[4] Department of Pharmaceutical Chemistry, Faculty of Pharmacy, Erciyes University, Kayseri 38039, Turkey.
[5] Department of Biochemistry, Faculty of Science, Selçuk University, Konya, Turkey.
[6] Department of Biochemistry, Faculty of Pharmacy, Cumhuriyet University, Sivas, Turkey.
* Corresponding Author. E-mail: uacar@anadolu.edu.tr; Tel. +90-222-335-0580/3775

Address: Anadolu University, Faculty of Pharmacy, Department of Pharmaceutical Chemistry, 38039, Eskişehir, Turkey.

 © 2023 Bentham Science Publishers

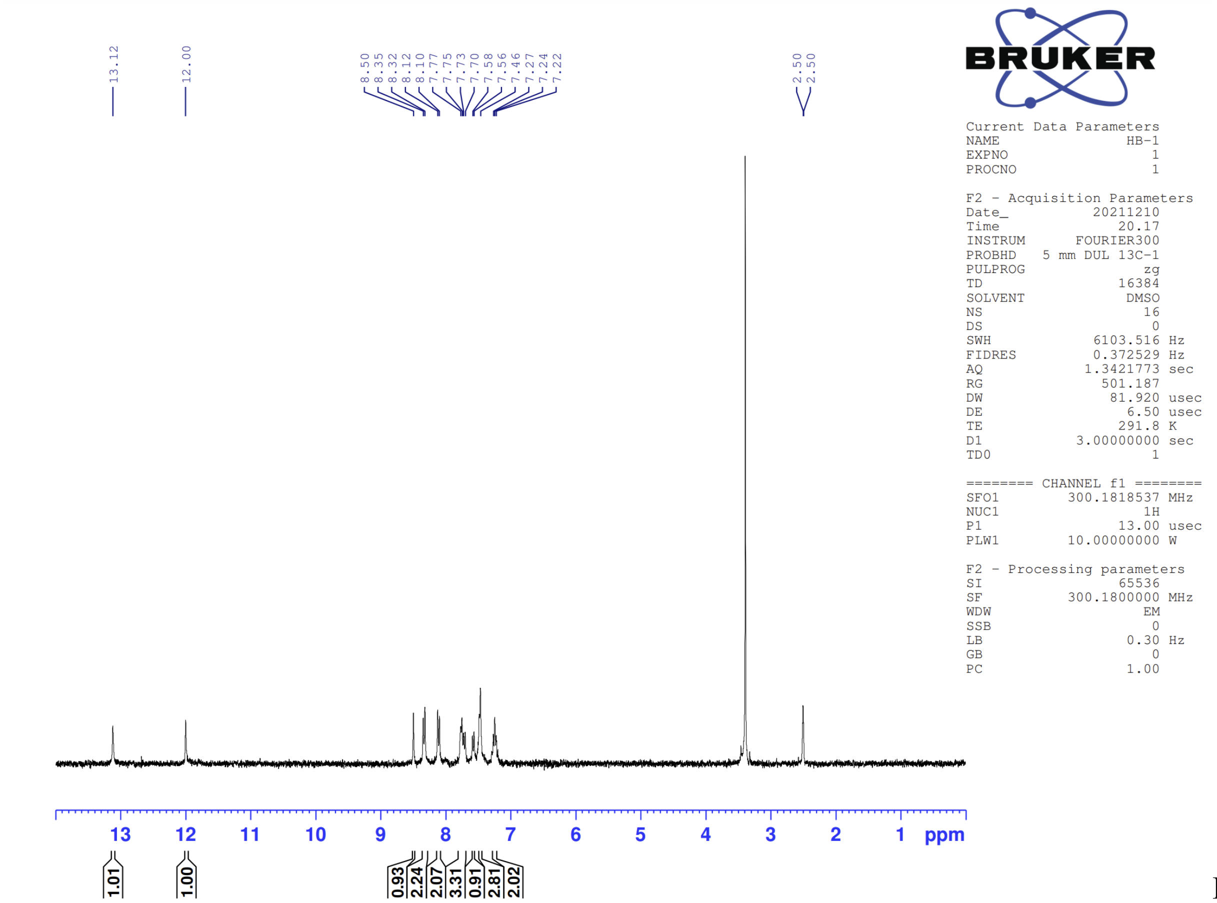

Figure 1.

^{1}H-NMR spectrum of compound **3a**

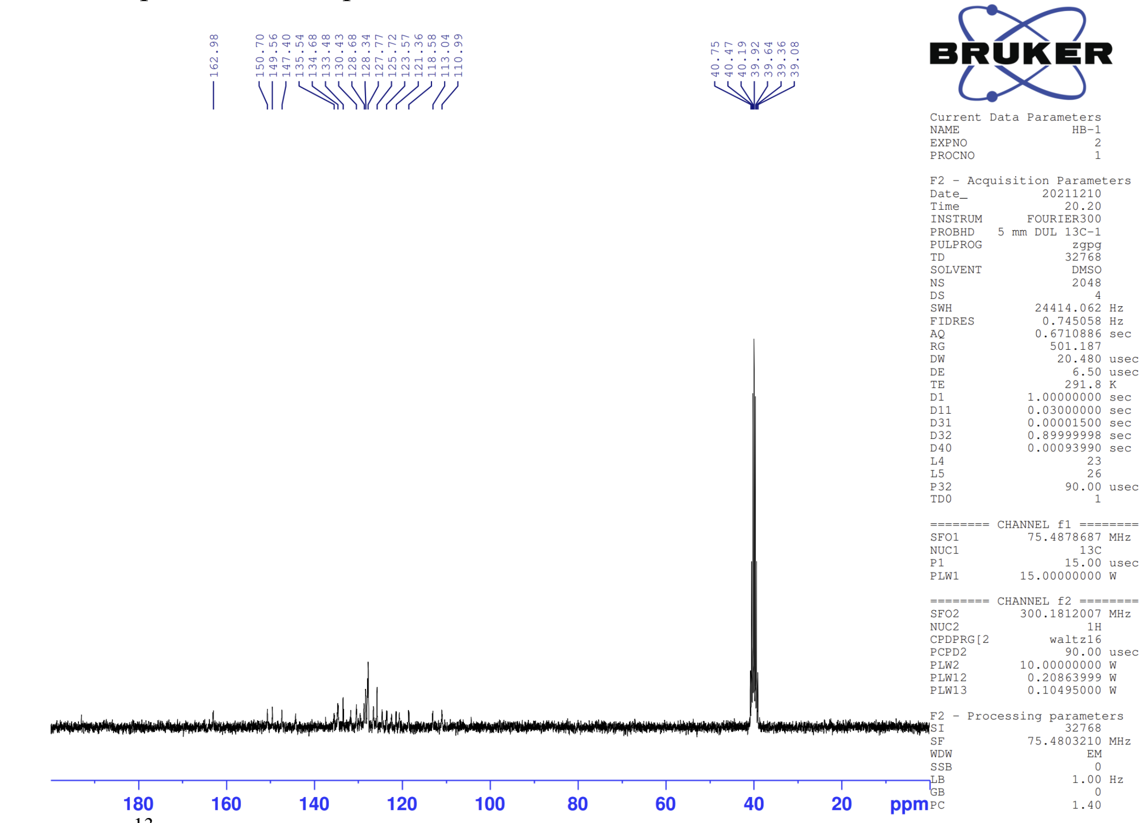

Figure 2. ^{13}C-NMR spectrum of compound **3a**

Formula Predictor Report - HB-1_51.lcd Page 1 of 1

Data File: C:\LabSolutions\Data\Analiz\uac\HB-1_51.lcd

Elmt	Val.	Min	Max	Elmt	Val.	Min	Max	Elmt	Val.	Min	Max	Elmt	Val.	Min	Max	Use Adduct
H	1	10	40	O	2	0	7	S	2	0	0	Ru	2	0	0	H
C	4	9	40	F	1	0	0	Cl	1	0	0	Pd	2	0	0	
N	3	4	6	P	3	0	0	Br	1	0	0	I	3	0	0	

Error Margin (ppm): 5
HC Ratio: unlimited
Max Isotopes: 3
MSn Iso RI (%): 10.00

DBE Range: 6.0 - 25.0
Apply N Rule: yes
Isotope RI (%): 1.00
MSn Logic Mode: AND

Electron Ions: both
Use MSn Info: yes
Isotope Res: 9000
Max Results: 150

Event#: 1 MS(E+) Ret. Time : 3.093 -> 3.093 Scan# : 465 -> 465

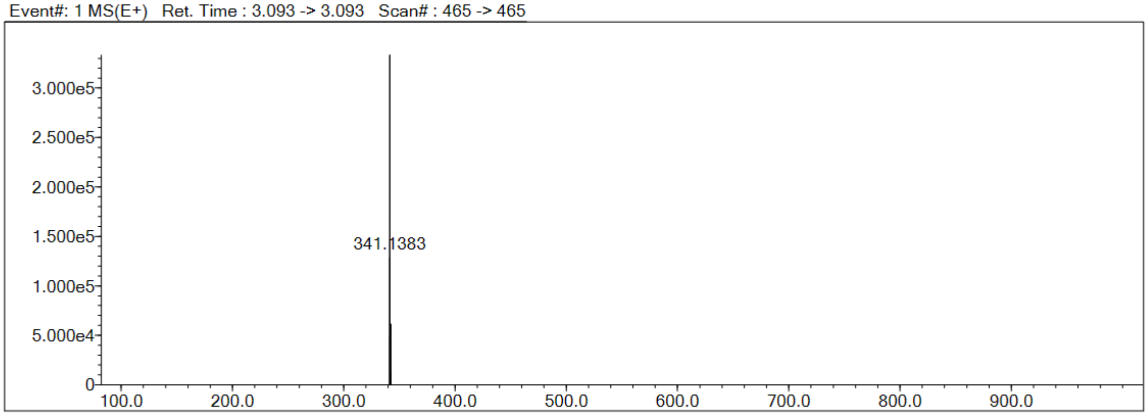

Measured region for 341.1383 m/z

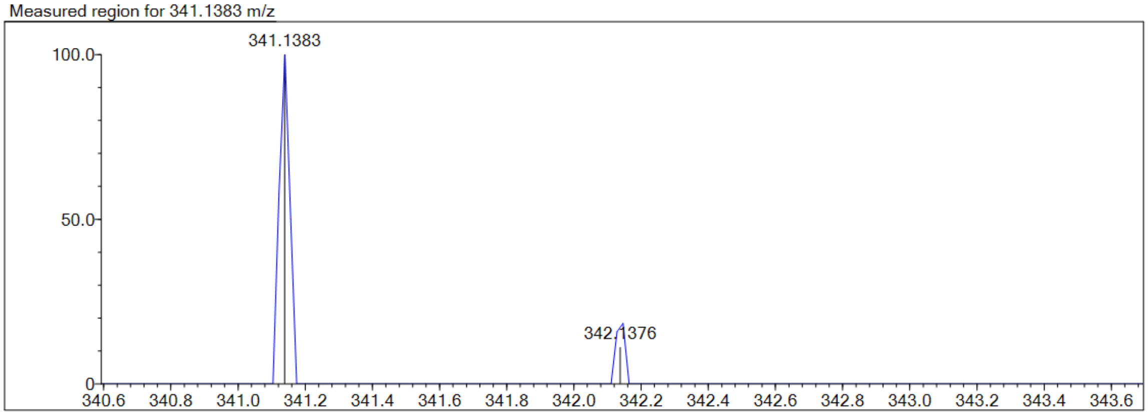

C21 H16 N4 O [M+H]+ : Predicted region for 341.1397 m/z

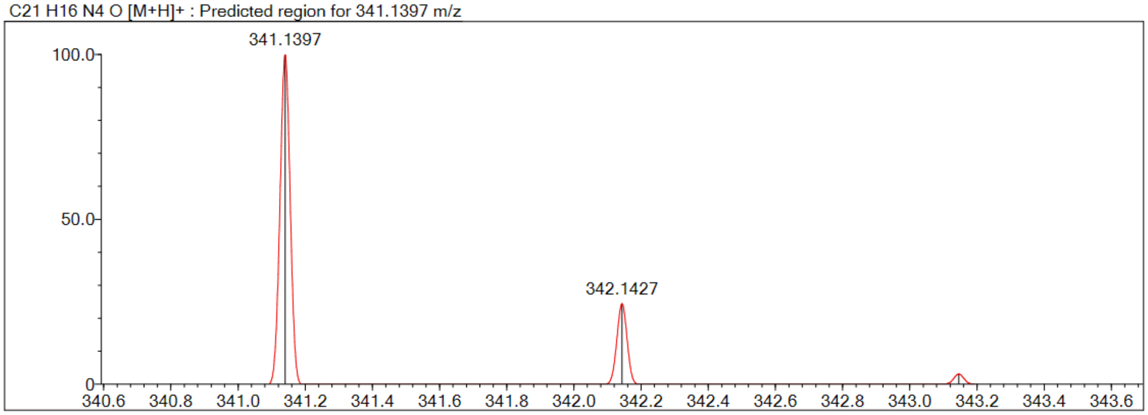

Rank	Score	Formula (M)	Ion	Meas. m/z	Pred. m/z	Df. (mDa)	Df. (ppm)	Iso	DBE
1	57.31	C21 H16 N4 O	[M+H]+	341.1383	341.1397	-1.4	-4.10	62.12	16.0

Figure 3. Mass spectrum of compound **3a**

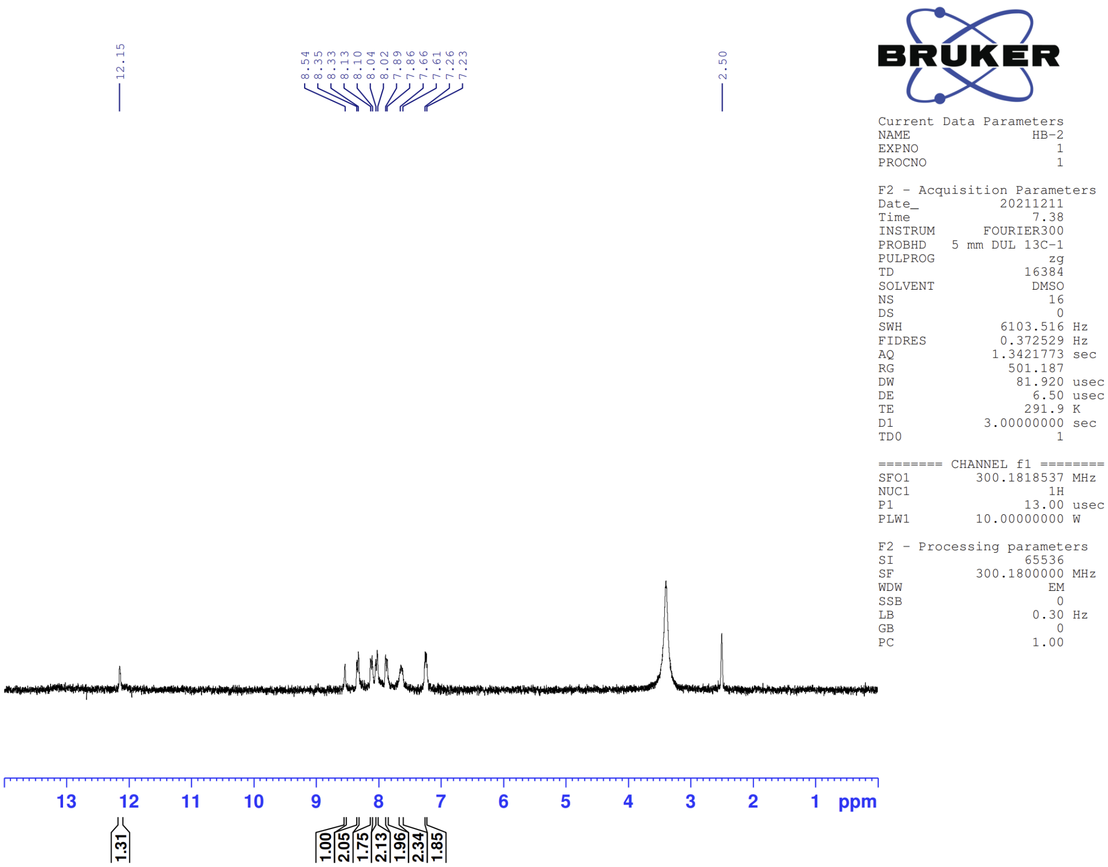

Figure 4. ¹H-NMR spectrum of compound **3b**

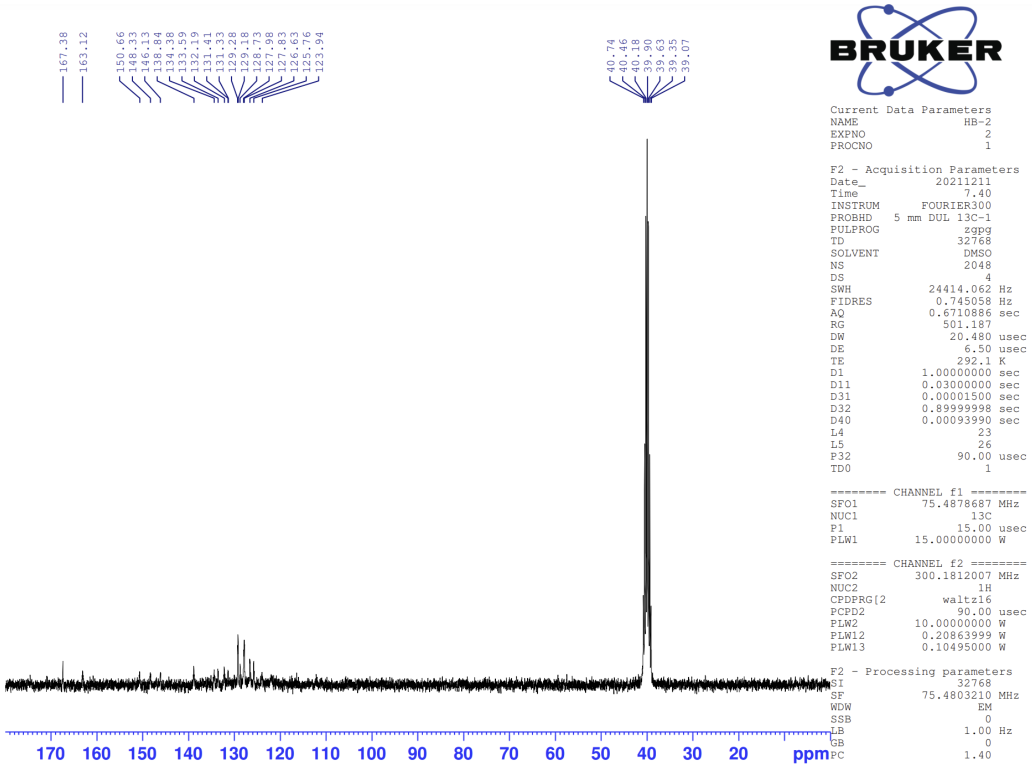

Figure 5. ¹³C-NMR spectrum of compound **3b**

Formula Predictor Report - HB-2_52.lcd Page 1 of 1

Data File: C:\LabSolutions\Data\Analiz\uac\HB-2_52.lcd

Elmt	Val.	Min	Max	Elmt	Val.	Min	Max	Elmt	Val.	Min	Max	Elmt	Val.	Min	Max	Use Adduct
H	1	10	40	O	2	0	4	S	2	0	0	Ru	2	0	0	H
C	4	9	40	F	1	0	0	Cl	1	0	0	Pd	2	0	0	
N	3	2	6	P	3	0	0	Br	1	0	0	I	3	0	0	

Error Margin (ppm):	5	DBE Range:	5.0 - 25.0	Electron Ions:	both
HC Ratio:	unlimited	Apply N Rule:	yes	Use MSn Info:	yes
Max Isotopes:	3	Isotope RI (%):	1.00	Isotope Res:	9000
MSn Iso RI (%):	10.00	MSn Logic Mode:	AND	Max Results:	150

Event#: 1 MS(E+) Ret. Time : 2.800 -> 2.987 Scan# : 421 -> 449

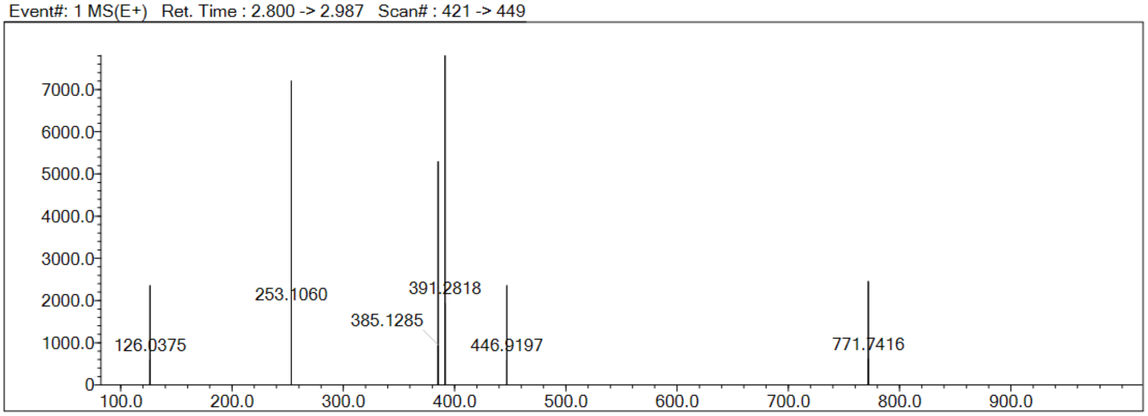

Measured region for 385.1285 m/z

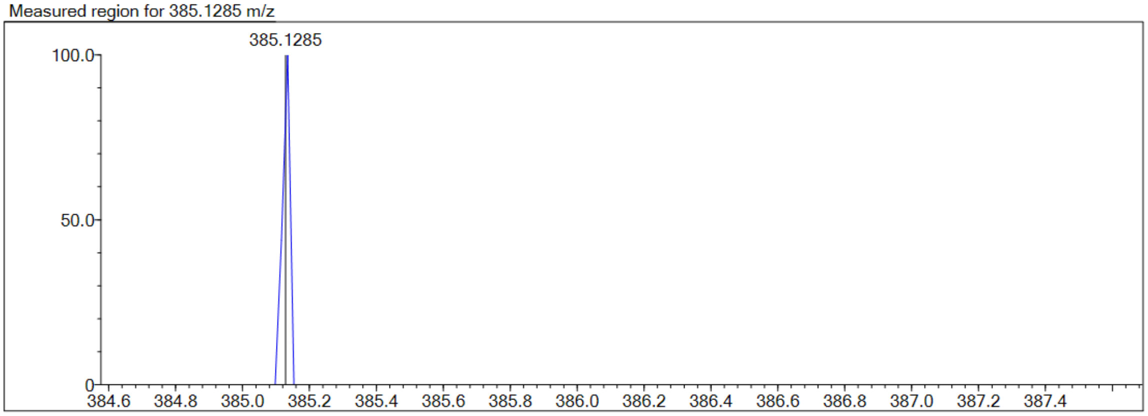

C22 H16 N4 O3 [M+H]+ : Predicted region for 385.1295 m/z

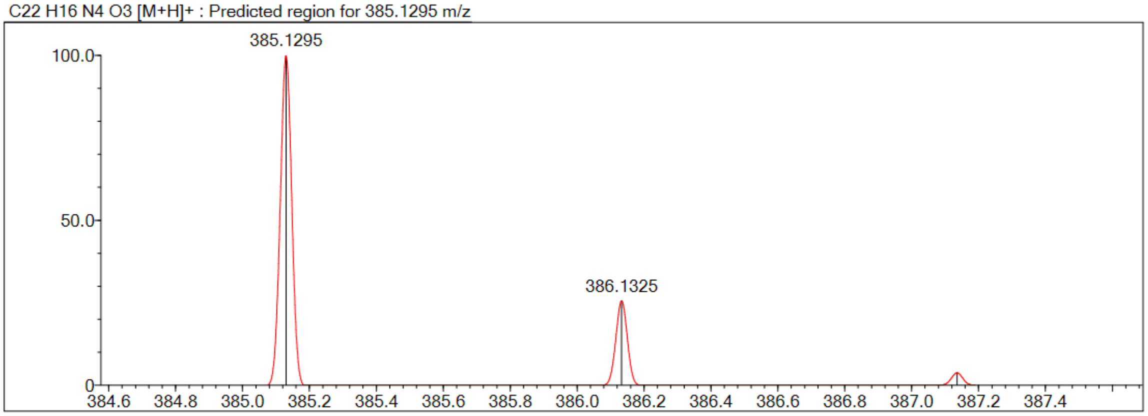

Rank	Score	Formula (M)	Ion	Meas. m/z	Pred. m/z	Df. (mDa)	Df. (ppm)	Iso	DBE
1	0.00	C22 H16 N4 O3	[M+H]+	385.1285	385.1295	-1.0	-2.60	0.00	17.0

Figure 6. Mass spectrum of compound **3b**

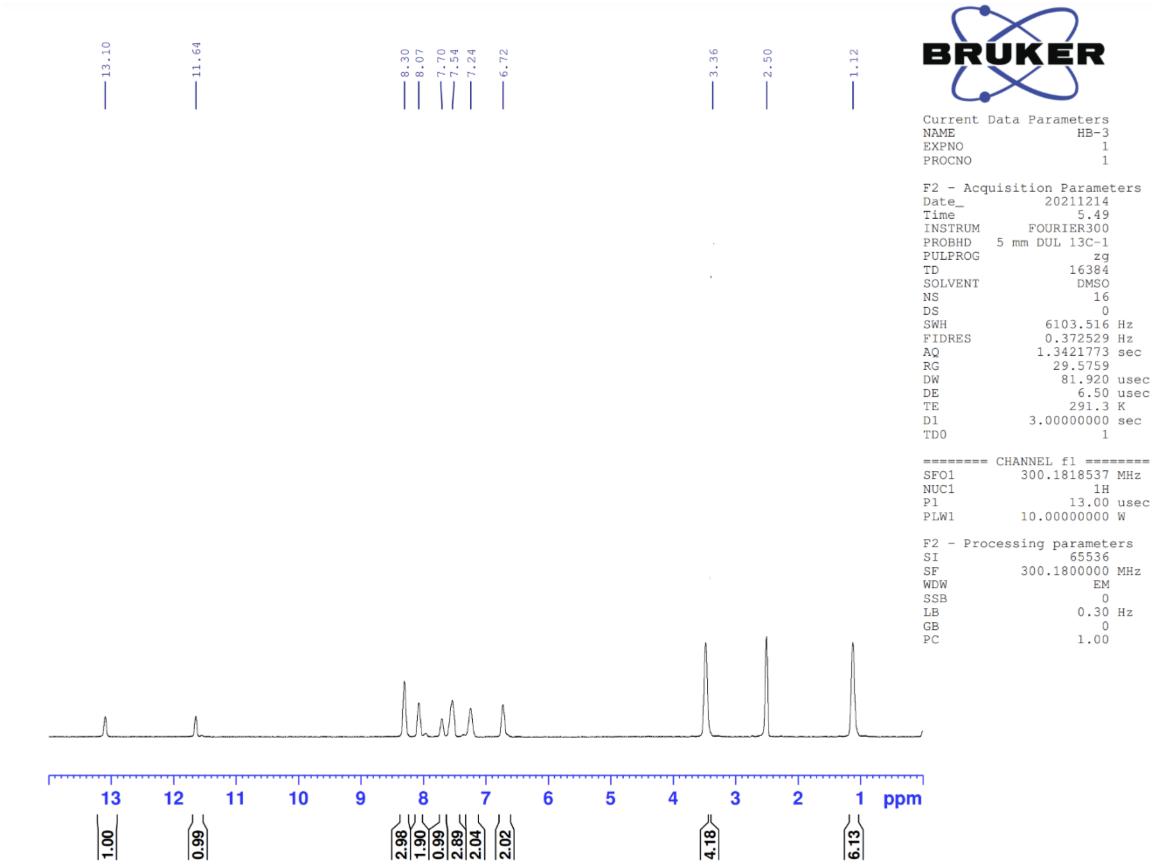

Figure 7. ^{1}H-NMR spectrum of compound **3c**

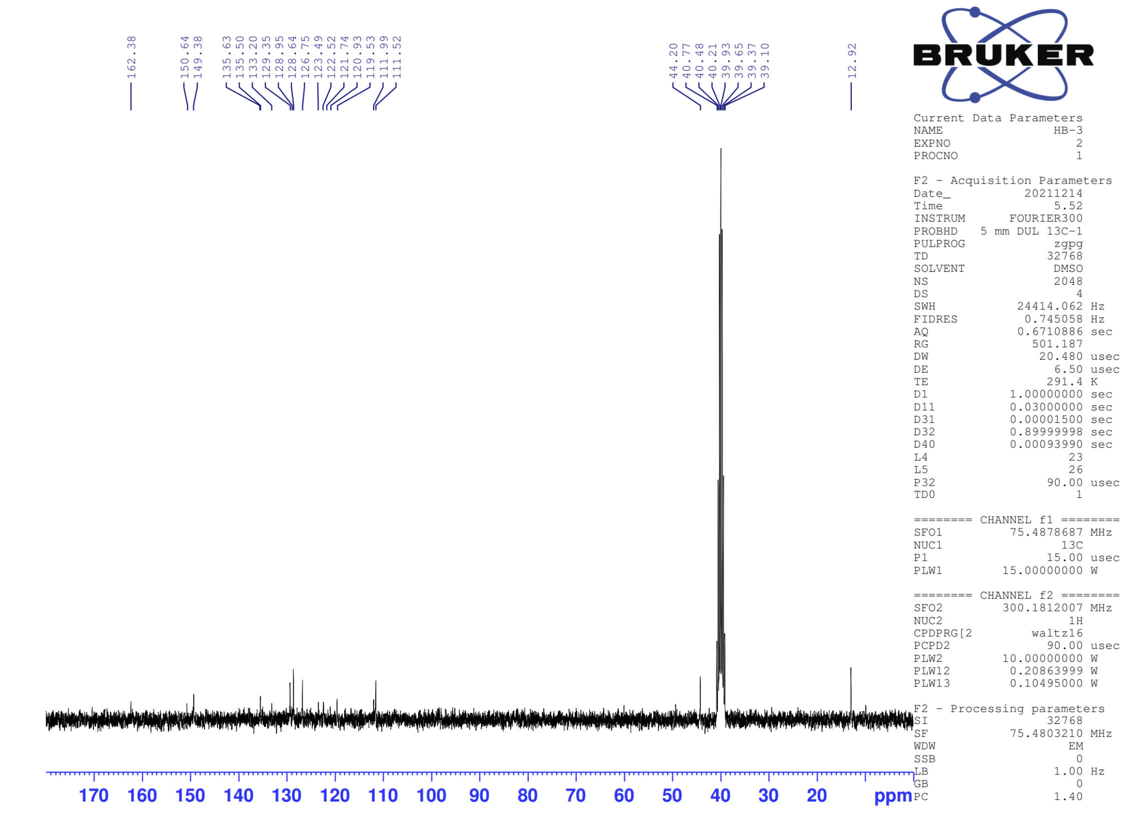

Figure 8. ^{13}C-NMR spectrum of compound **3c**

Formula Predictor Report - HB-3_53.lcd Page 1 of 1

Data File: C:\LabSolutions\Data\Analiz\uac\HB-3_53.lcd

Elmt	Val.	Min	Max	Elmt	Val.	Min	Max	Elmt	Val.	Min	Max	Elmt	Val.	Min	Max	Use Adduct
H	1	10	40	O	2	0	7	S	2	0	0	Ru	2	0	0	H
C	4	9	40	F	1	0	0	Cl	1	0	0	Pd	2	0	0	
N	3	4	6	P	3	0	0	Br	1	0	0	I	3	0	0	

Error Margin (ppm):	5	DBE Range:	6.0 - 25.0	Electron Ions:	both
HC Ratio:	unlimited	Apply N Rule:	yes	Use MSn Info:	yes
Max Isotopes:	3	Isotope RI (%):	1.00	Isotope Res:	9000
MSn Iso RI (%):	10.00	MSn Logic Mode:	AND	Max Results:	150

Event#: 1 MS(E+) Ret. Time : 3.587 Scan# : 539

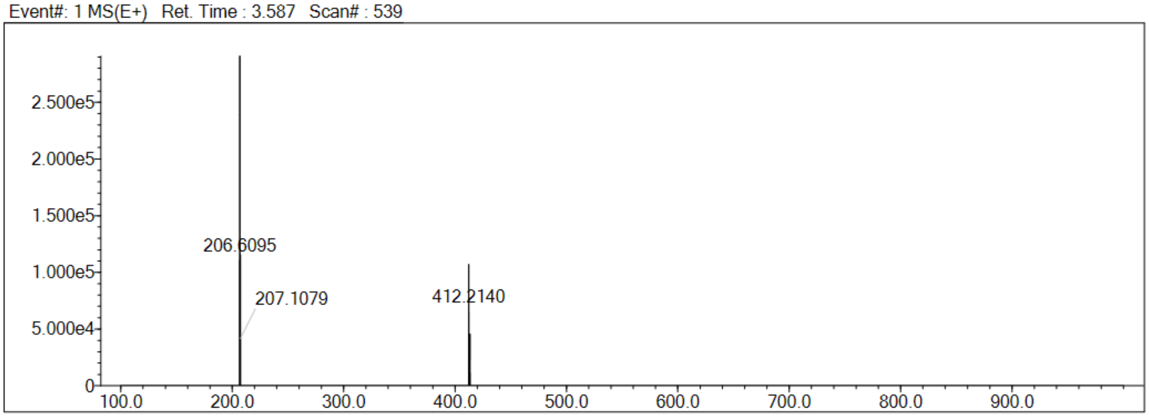

Measured region for 412.2140 m/z

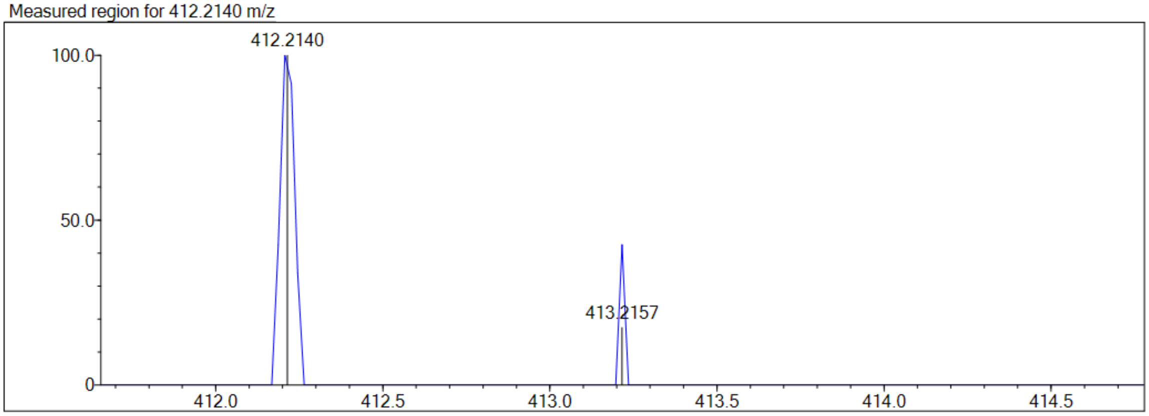

C25 H25 N5 O [M+H]+ : Predicted region for 412.2132 m/z

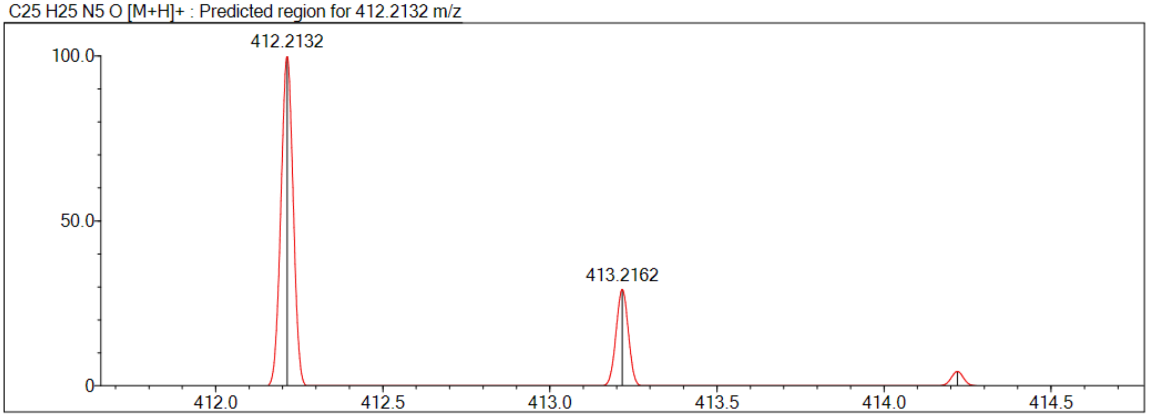

Rank	Score	Formula (M)	Ion	Meas. m/z	Pred. m/z	Df. (mDa)	Df. (ppm)	Iso	DBE
1	60.80	C25 H25 N5 O	[M+H]+	412.2140	412.2132	0.8	1.94	62.26	16.0

Figure 9. Mass spectrum of compound **3c**

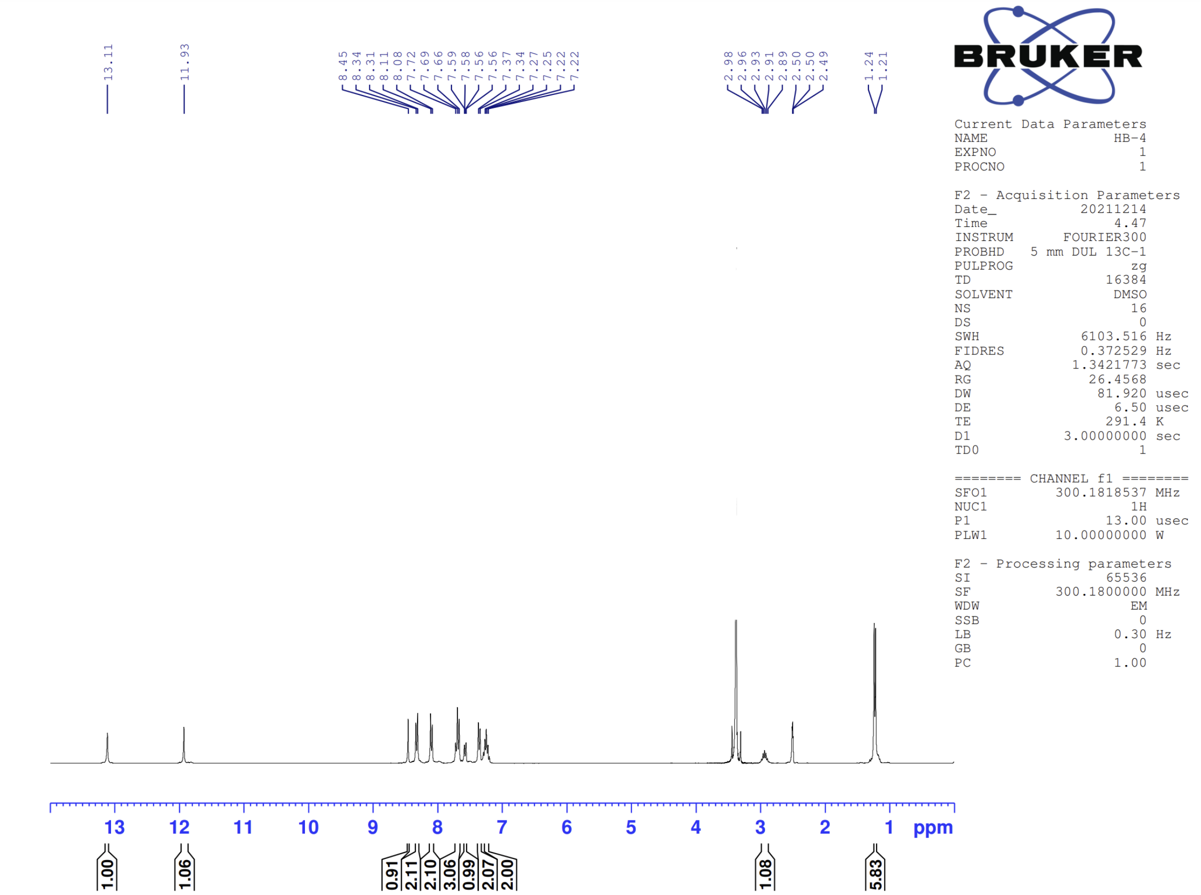

Figure 10.

^{1}H-NMR spectrum of compound **3d**

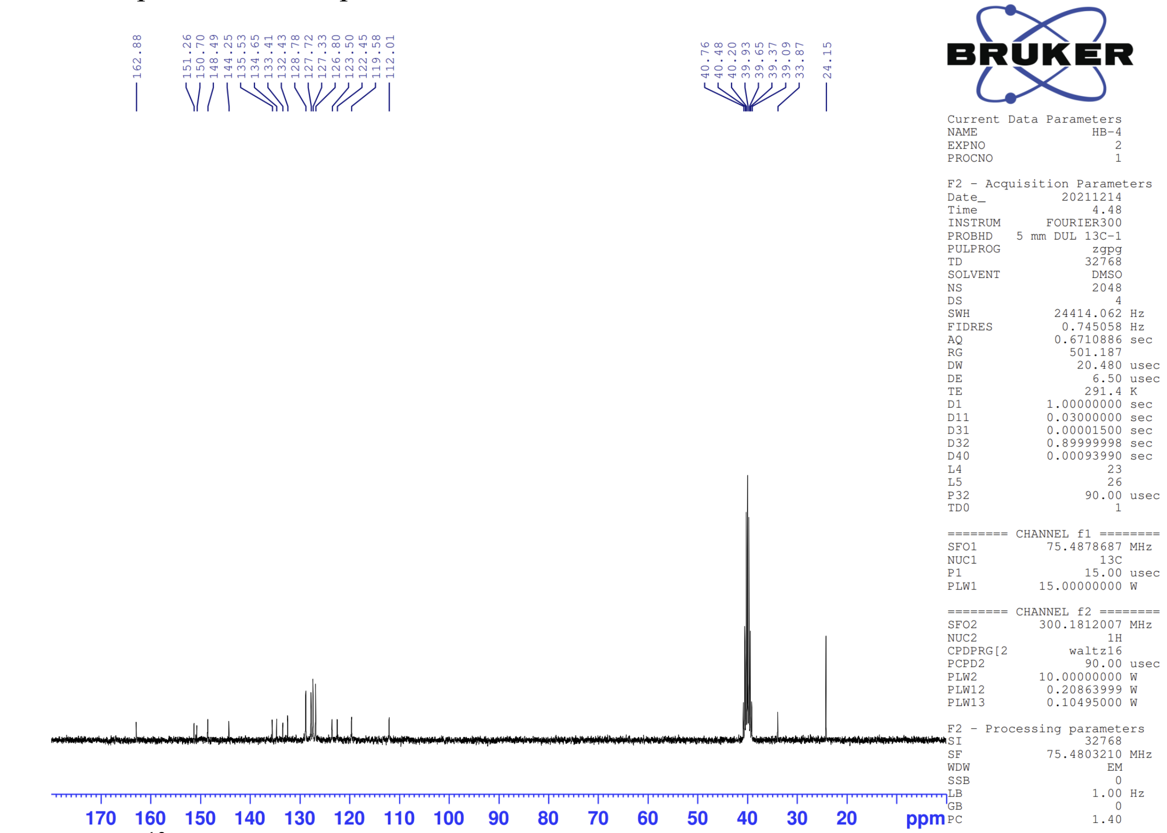

Figure 11. ^{13}C-NMR spectrum of compound **3d**

Formula Predictor Report - HB-4_54.lcd Page 1 of 1

Data File: C:\LabSolutions\Data\Analiz\uac\HB-4_54.lcd

Elmt	Val.	Min	Max	Elmt	Val.	Min	Max	Elmt	Val.	Min	Max	Elmt	Val.	Min	Max	Use Adduct
H	1	10	40	O	2	0	7	S	2	0	0	Ru	2	0	0	H
C	4	9	40	F	1	0	0	Cl	1	0	0	Pd	2	0	0	
N	3	4	6	P	3	0	0	Br	1	0	0	I	3	0	0	

Error Margin (ppm): 5
HC Ratio: unlimited
Max Isotopes: 3
MSn Iso RI (%): 10.00

DBE Range: 6.0 - 25.0
Apply N Rule: yes
Isotope RI (%): 1.00
MSn Logic Mode: AND

Electron Ions: both
Use MSn Info: yes
Isotope Res: 9000
Max Results: 150

Event#: 1 MS(E+) Ret. Time : 3.987 Scan# : 599

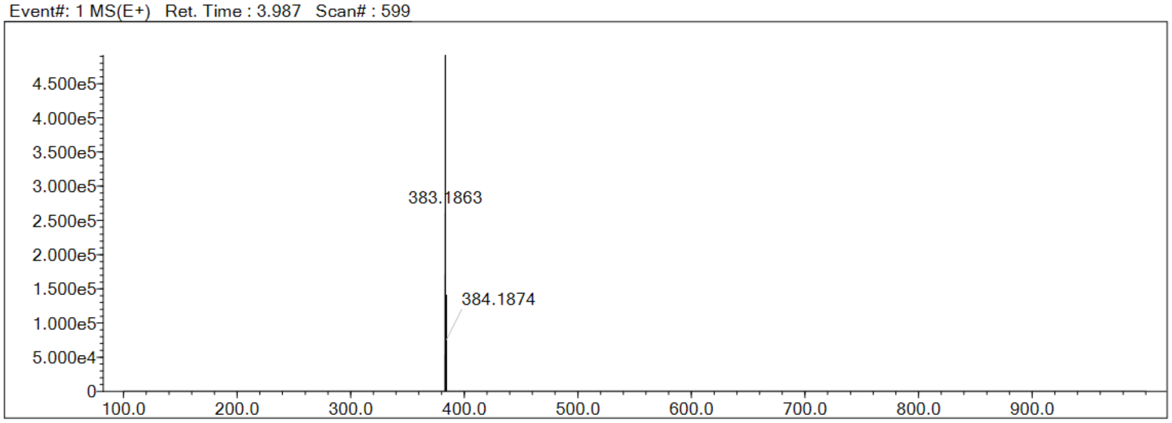

Measured region for 383.1863 m/z

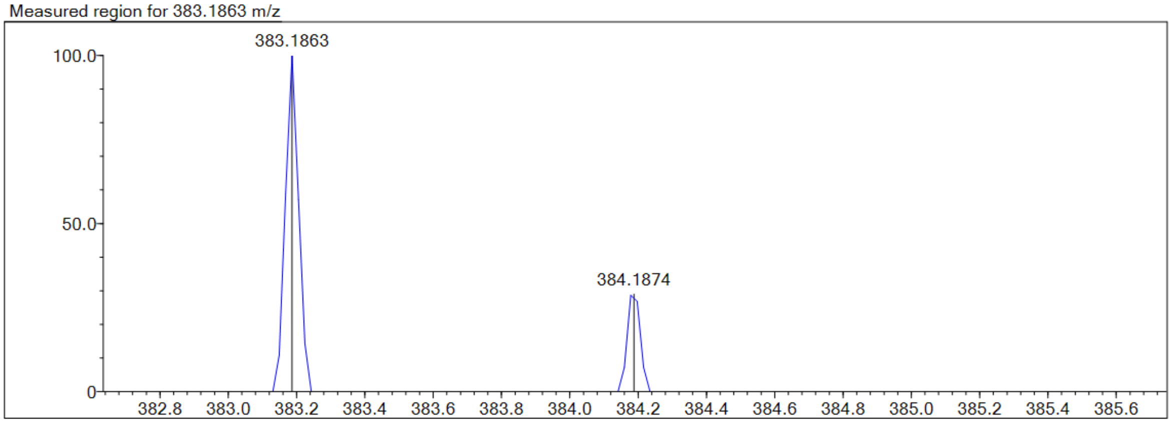

C24 H22 N4 O [M+H]+ : Predicted region for 383.1866 m/z

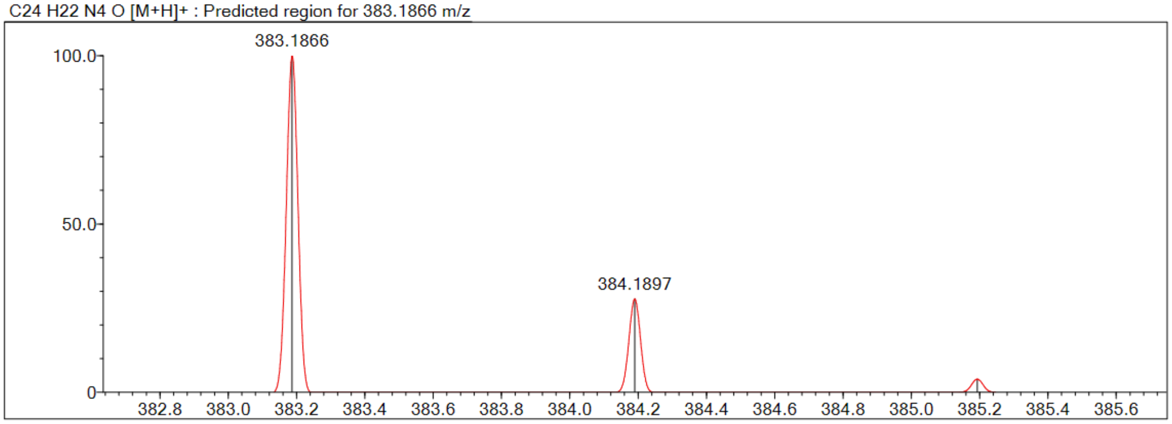

Rank	Score	Formula (M)	Ion	Meas. m/z	Pred. m/z	Df. (mDa)	Df. (ppm)	Iso	DBE
1	100.00	C24 H22 N4 O	[M+H]+	383.1863	383.1866	-0.3	-0.78	100.00	16.0

Figure 12. Mass spectrum of compound **3d**

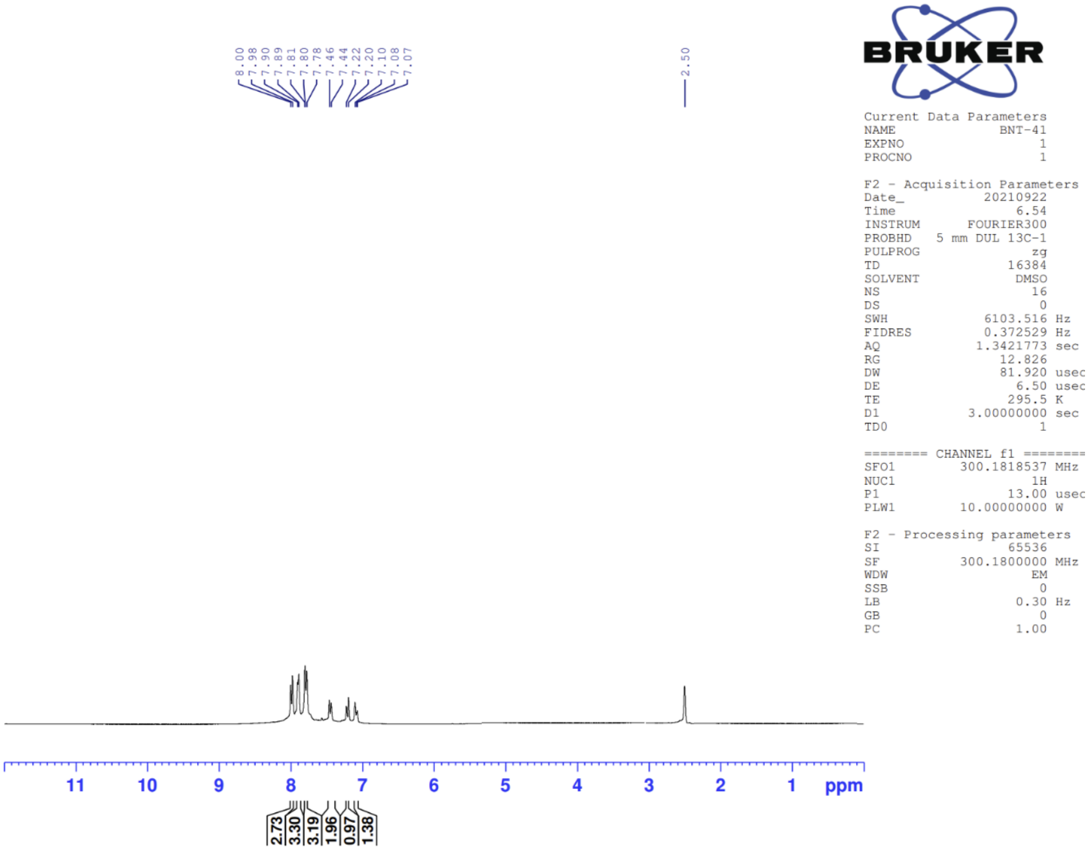

Figure 13. ^{1}H-NMR spectrum of compound **3e**

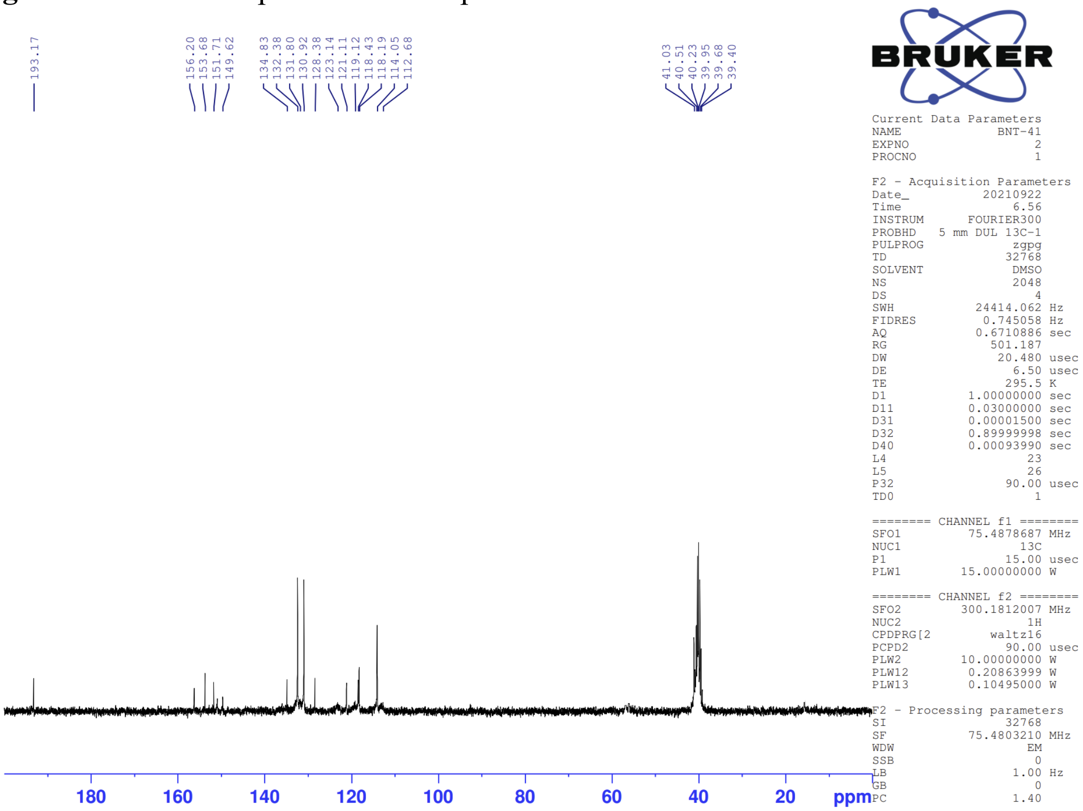

Figure 14. ^{13}C-NMR spectrum of compound **3e**

Formula Predictor Report - HB-5_55.lcd Page 1 of 1

Data File: C:\LabSolutions\Data\Analiz\uac\HB-5_55.lcd

Elmt	Val.	Min	Max	Elmt	Val.	Min	Max	Elmt	Val.	Min	Max	Elmt	Val.	Min	Max	Use Adduct
H	1	10	40	O	2	0	7	S	2	0	0	Ru	2	0	0	H
C	4	9	40	F	1	0	0	Cl	1	1	1	Pd	2	0	0	
N	3	4	6	P	3	0	0	Br	1	0	0	I	3	0	0	

Error Margin (ppm):	5	DBE Range:	5.0 - 20.0	Electron Ions:	both
HC Ratio:	unlimited	Apply N Rule:	yes	Use MSn Info:	yes
Max Isotopes:	3	Isotope RI (%):	1.00	Isotope Res:	9000
MSn Iso RI (%):	10.00	MSn Logic Mode:	AND	Max Results:	150

Event#: 1 MS(E+) Ret. Time : 3.467 Scan# : 521

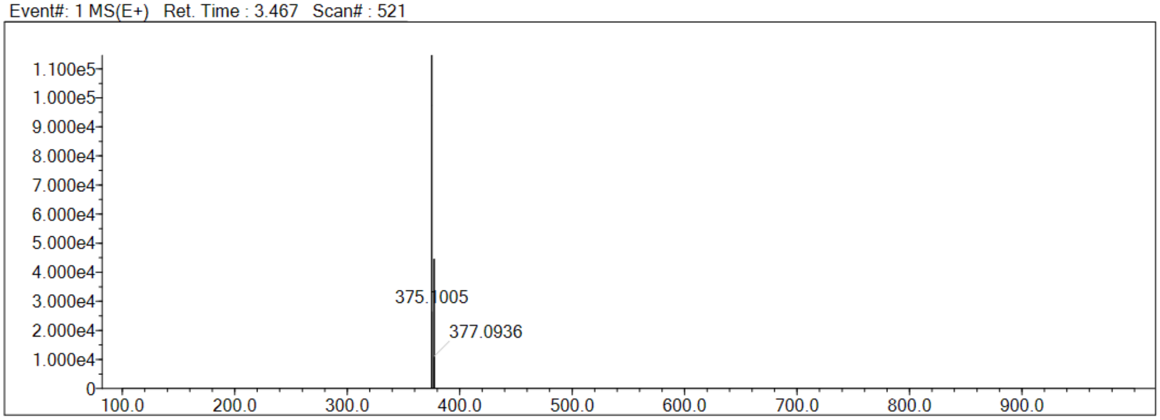

Measured region for 375.1005 m/z

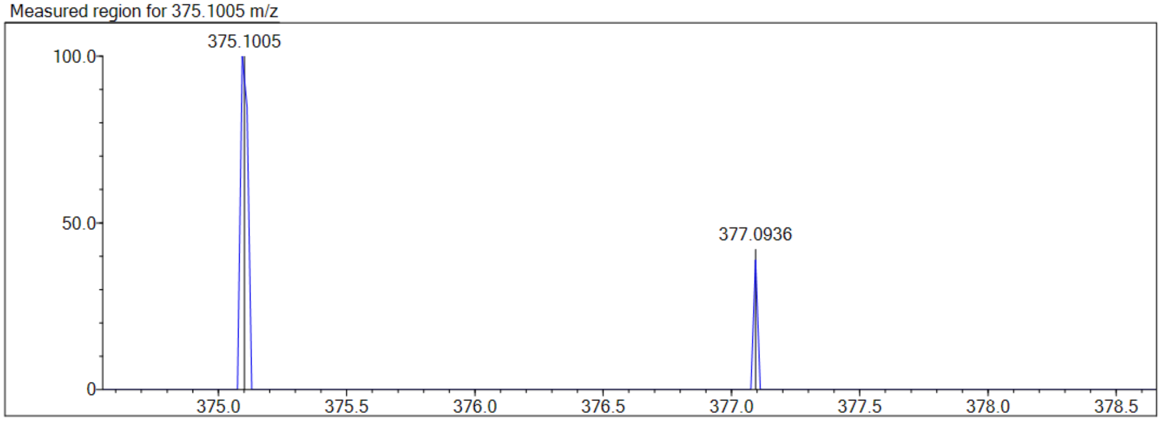

C21 H15 N4 O Cl [M+H]+ : Predicted region for 375.1007 m/z

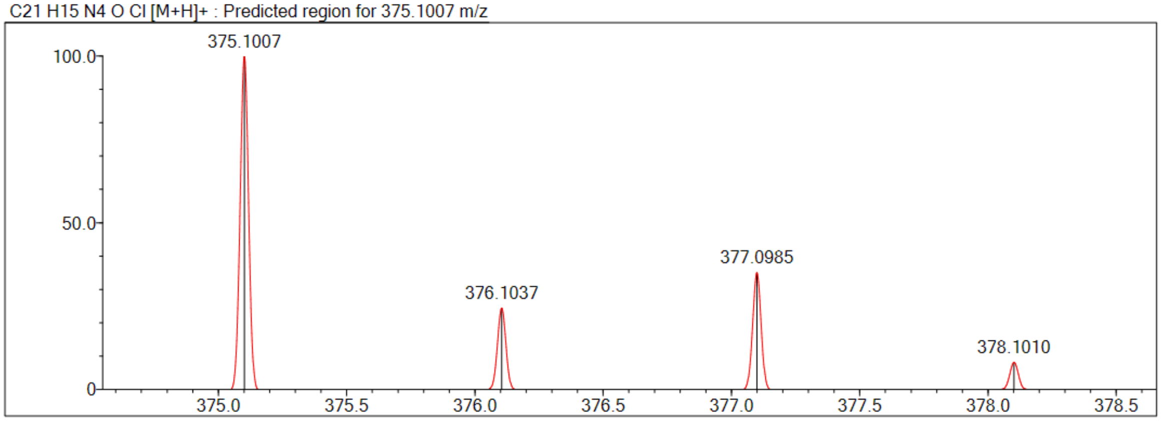

Rank	Score	Formula (M)	Ion	Meas. m/z	Pred. m/z	Df. (mDa)	Df. (ppm)	Iso	DBE
1	53.52	C21 H15 N4 O Cl	[M+H]+	375.1005	375.1007	-0.2	-0.53	53.52	16.0

Figure 15. Mass spectrum of compound **3e**

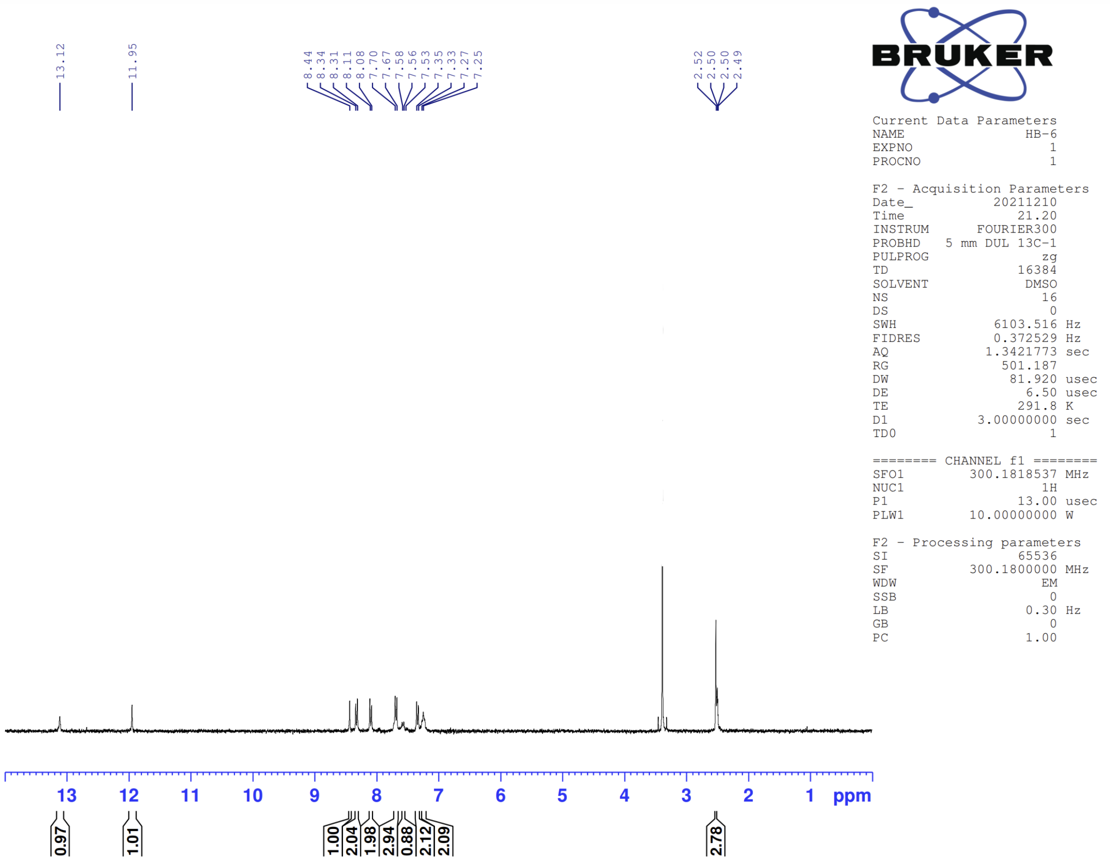

Figure 16. ^{1}H-NMR spectrum of compound **3f**

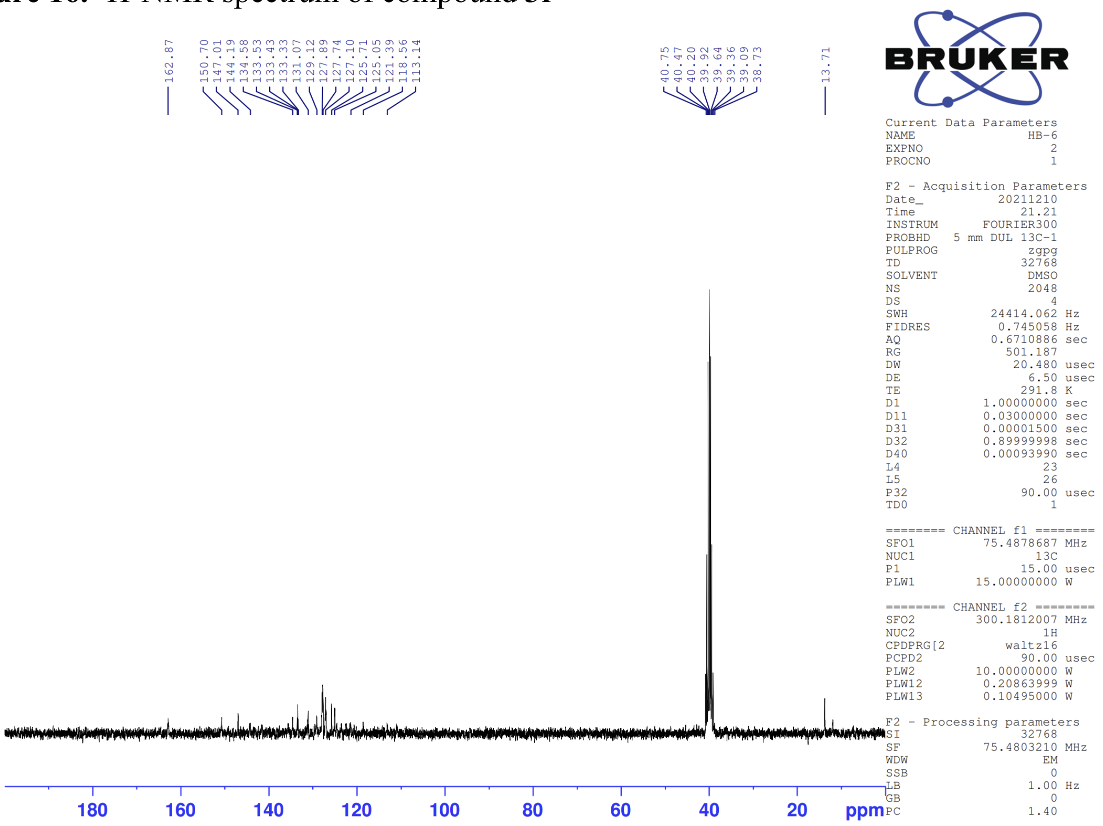

Figure 17. ^{13}C-NMR spectrum of compound **3f**

Formula Predictor Report - HB-6_56.lcd Page 1 of 1

Data File: C:\LabSolutions\Data\Analiz\uac\HB-6_56.lcd

Elmt	Val.	Min	Max	Elmt	Val.	Min	Max	Elmt	Val.	Min	Max	Elmt	Val.	Min	Max	Use Adduct
H	1	10	40	O	2	0	7	S	2	1	1	Ru	2	0	0	H
C	4	9	40	F	1	0	0	Cl	1	0	0	Pd	2	0	0	
N	3	4	6	P	3	0	0	Br	1	0	0	I	3	0	0	

Error Margin (ppm):	5	DBE Range:	5.0 - 20.0	Electron Ions:	both
HC Ratio:	unlimited	Apply N Rule:	yes	Use MSn Info:	yes
Max Isotopes:	3	Isotope RI (%):	1.00	Isotope Res:	9000
MSn Iso RI (%):	10.00	MSn Logic Mode:	AND	Max Results:	150

Event#: 1 MS(E+) Ret. Time : 3.480 Scan# : 523

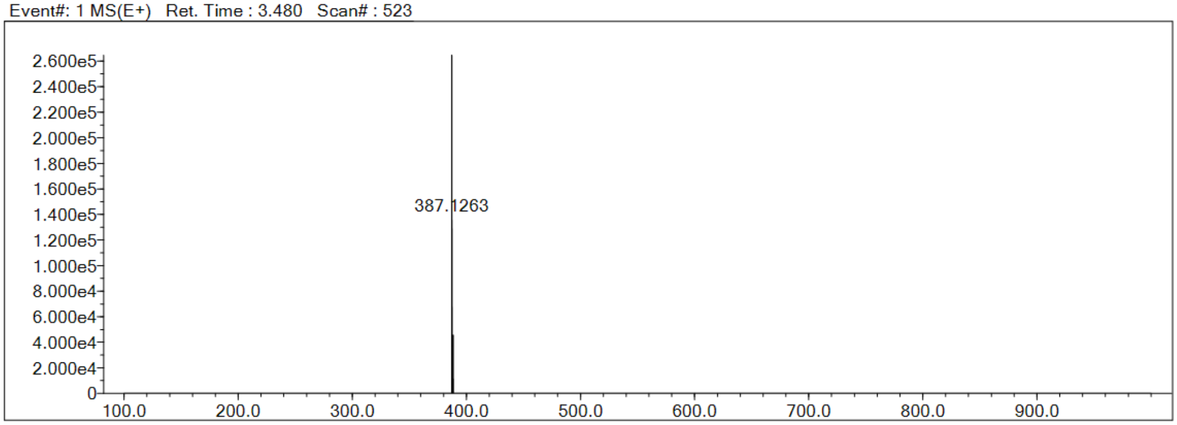

Measured region for 387.1263 m/z

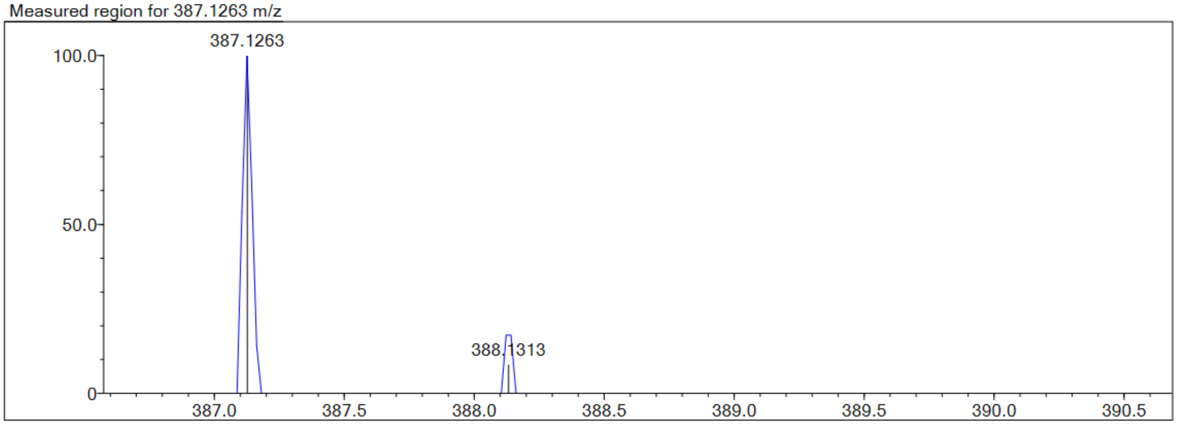

C22 H18 N4 O S [M+H]+ : Predicted region for 387.1274 m/z

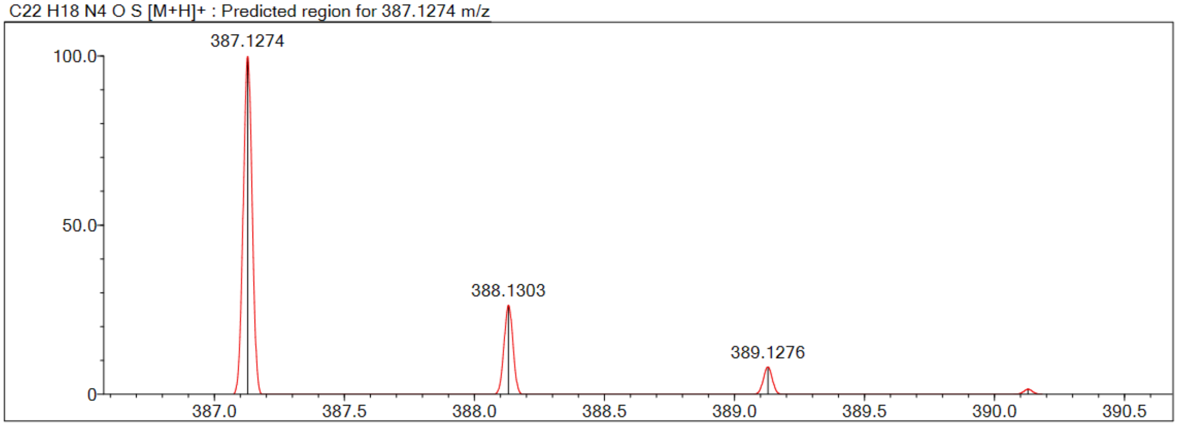

Rank	Score	Formula (M)	Ion	Meas. m/z	Pred. m/z	Df. (mDa)	Df. (ppm)	Iso	DBE
1	0.00	C22 H18 N4 O S	[M+H]+	387.1263	387.1274	-1.1	-2.84	0.00	16.0

Figure 18. Mass spectrum of compound **3f**

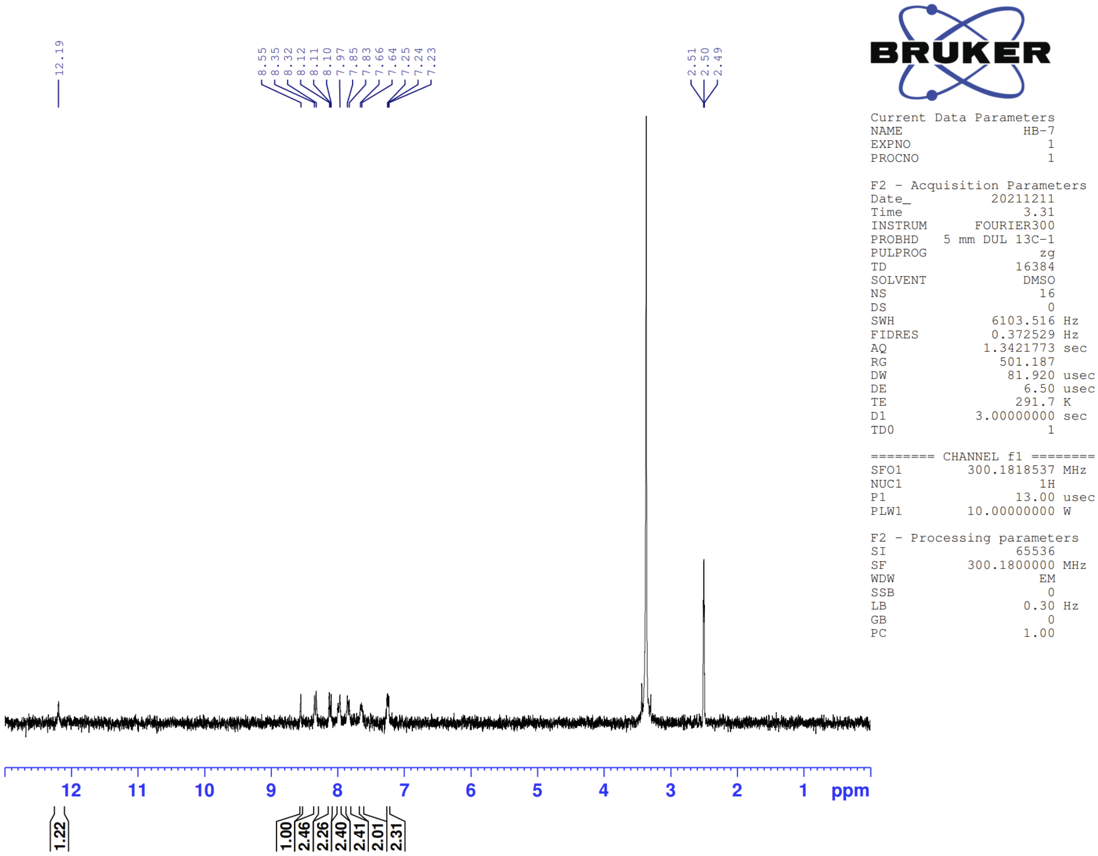

Figure 19. ^{1}H-NMR spectrum of compound **3g**

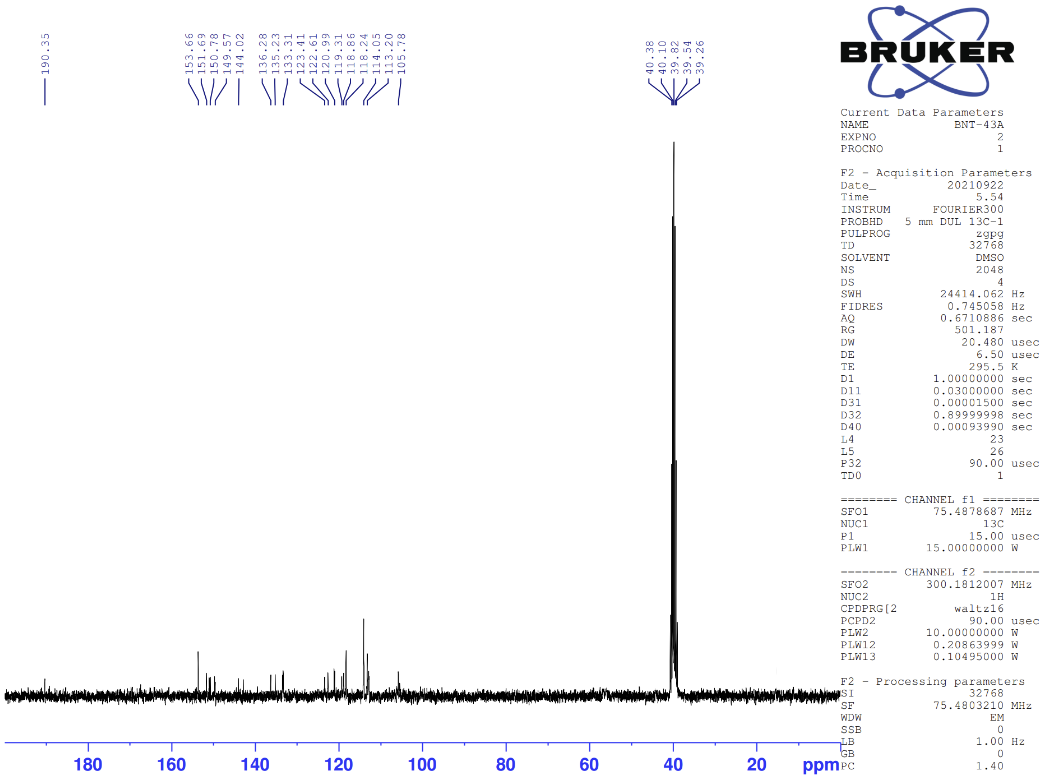

Figure 20. ^{13}C-NMR spectrum of compound **3g**

Formula Predictor Report - HB-7_57.lcd Page 1 of 1

Data File: C:\LabSolutions\Data\Analiz\uac\HB-7_57.lcd

Elmt	Val.	Min	Max	Elmt	Val.	Min	Max	Elmt	Val.	Min	Max	Elmt	Val.	Min	Max	Use Adduct
H	1	10	40	O	2	1	3	S	2	0	0	Ru	2	0	0	H
C	4	9	40	F	1	1	3	Cl	1	0	0	Pd	2	0	0	
N	3	4	6	P	3	0	0	Br	1	0	0	I	3	0	0	

Error Margin (ppm): 5
HC Ratio: unlimited
Max Isotopes: 3
MSn Iso RI (%): 10.00

DBE Range: 5.0 - 20.0
Apply N Rule: yes
Isotope RI (%): 1.00
MSn Logic Mode: AND

Electron Ions: both
Use MSn Info: yes
Isotope Res: 9000
Max Results: 150

Event#: 1 MS(E+) Ret. Time : 3.533 Scan# : 531

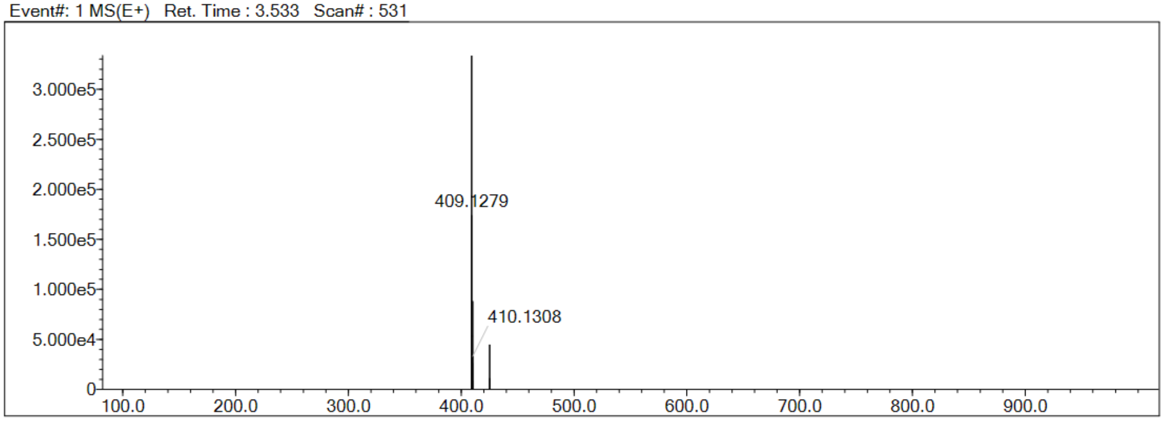

Measured region for 409.1279 m/z

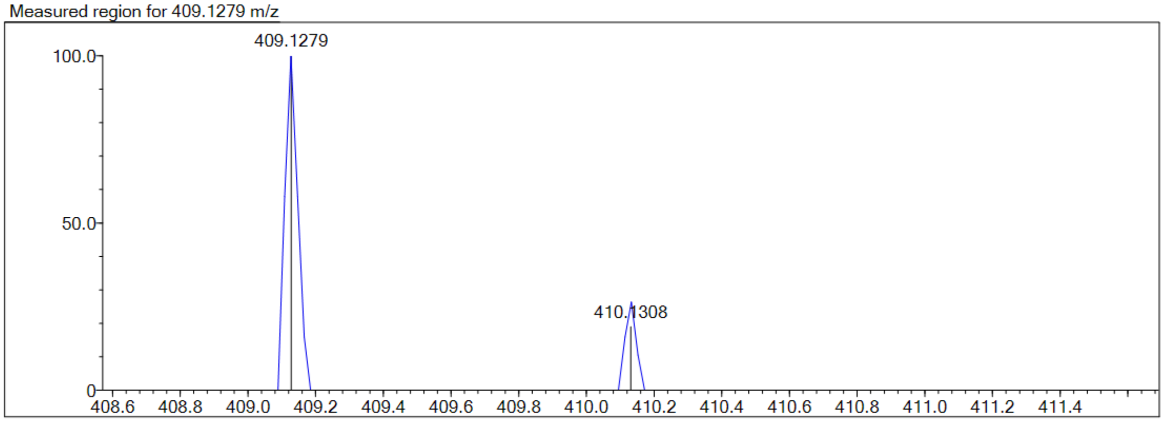

C22 H15 N4 O F3 [M+H]+ : Predicted region for 409.1271 m/z

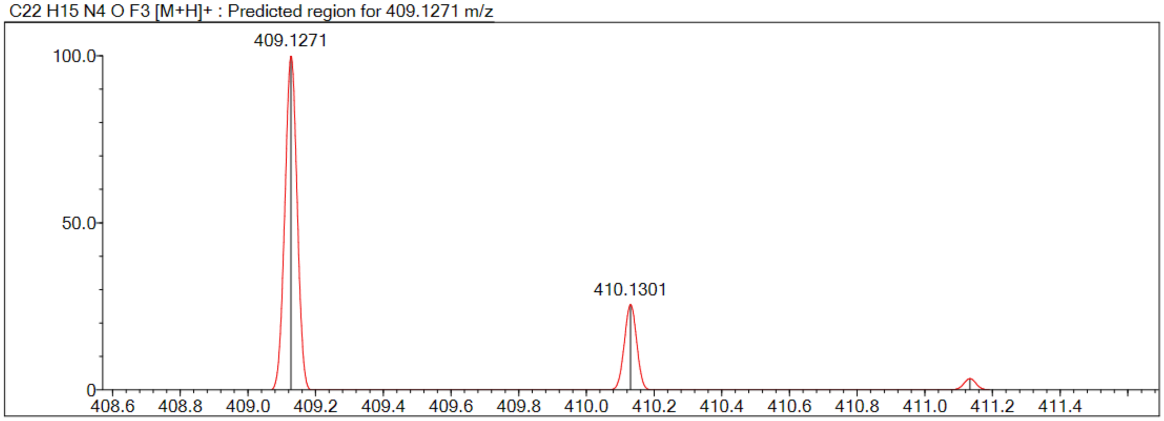

Rank	Score	Formula (M)	Ion	Meas. m/z	Pred. m/z	Df. (mDa)	Df. (ppm)	Iso	DBE
1	96.86	C22 H15 N4 O F3	[M+H]+	409.1279	409.1271	0.8	1.96	99.24	16.0

Figure 21. Mass spectrum of compound **3g**

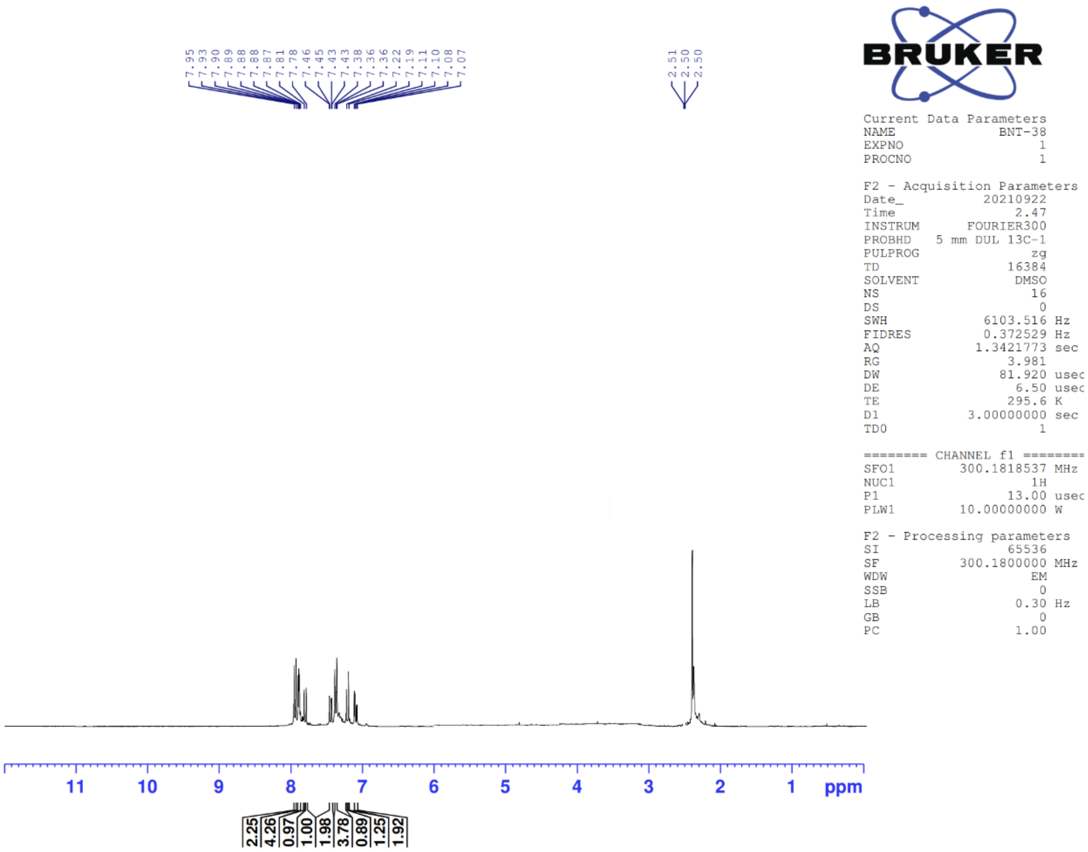

Figure 22. ^{1}H-NMR spectrum of compound **3h**

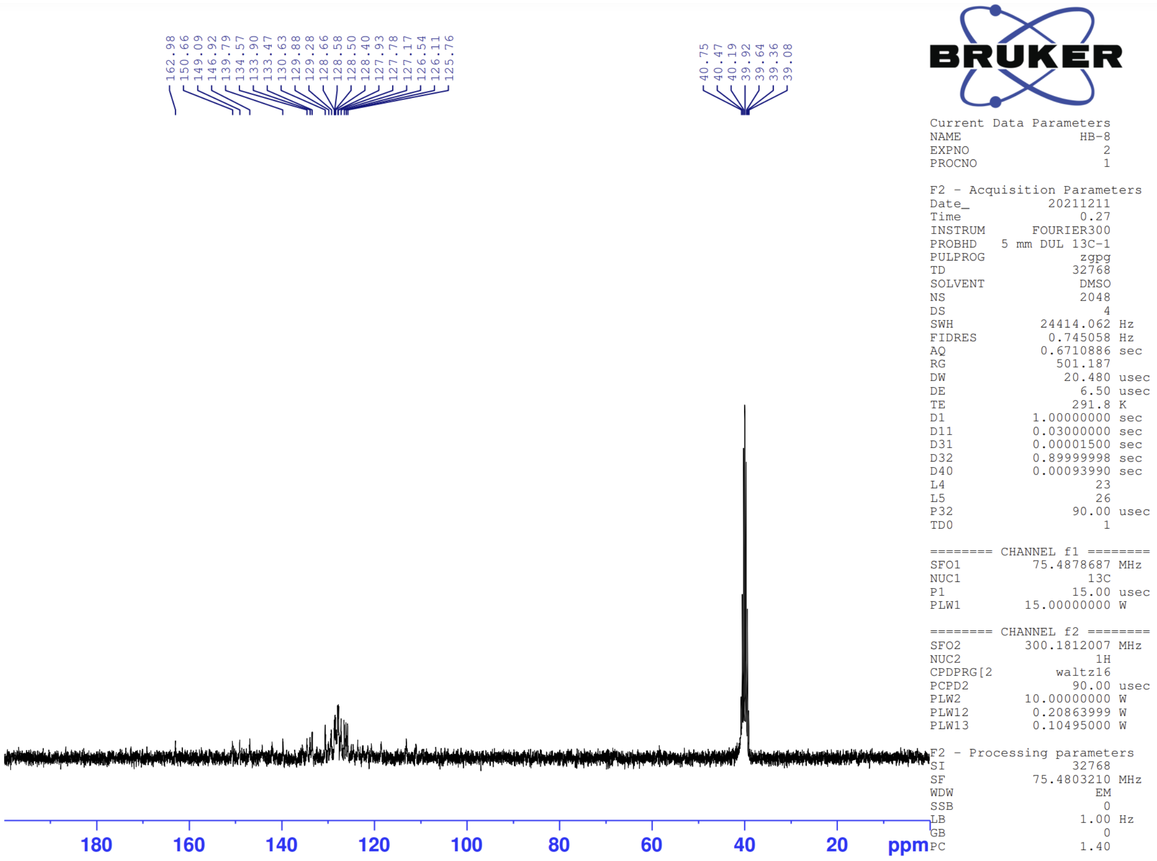

Figure 23.

^{13}C-NMR spectrum of compound **3h**

Formula Predictor Report - HB-8_58.lcd

Page 1 of 1

Data File: C:\LabSolutions\Data\Analiz\uac\HB-8_58.lcd

Elmt	Val.	Min	Max	Elmt	Val.	Min	Max	Elmt	Val.	Min	Max	Elmt	Val.	Min	Max	Use Adduct
H	1	10	40	O	2	1	3	S	2	0	0	Ru	2	0	0	H
C	4	9	40	F	1	0	0	Cl	1	0	0	Pd	2	0	0	
N	3	4	6	P	3	0	0	Br	1	0	0	I	3	0	0	

Error Margin (ppm): 5
HC Ratio: unlimited
Max Isotopes: 3
MSn Iso RI (%): 10.00

DBE Range: 5.0 - 20.0
Apply N Rule: yes
Isotope RI (%): 1.00
MSn Logic Mode: AND

Electron Ions: both
Use MSn Info: yes
Isotope Res: 9000
Max Results: 150

Event#: 1 MS(E+) Ret. Time : 3.933 Scan# : 591

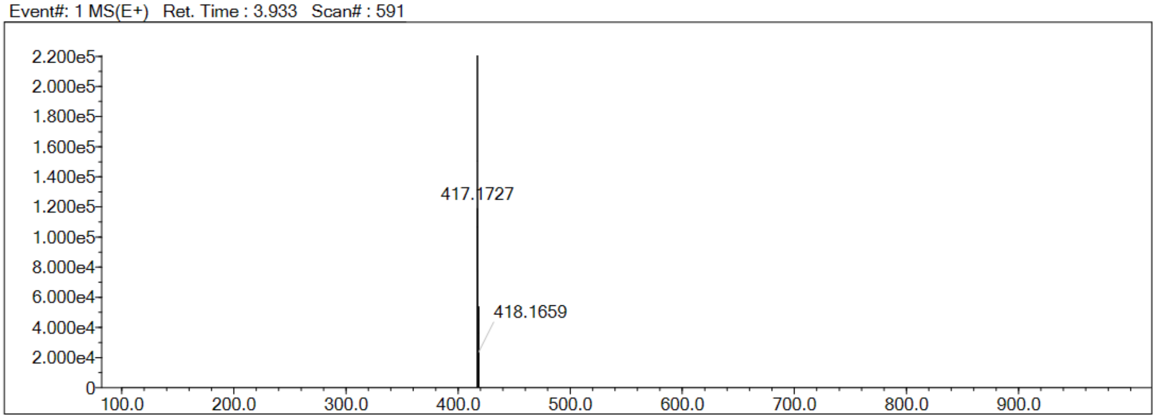

Measured region for 417.1727 m/z

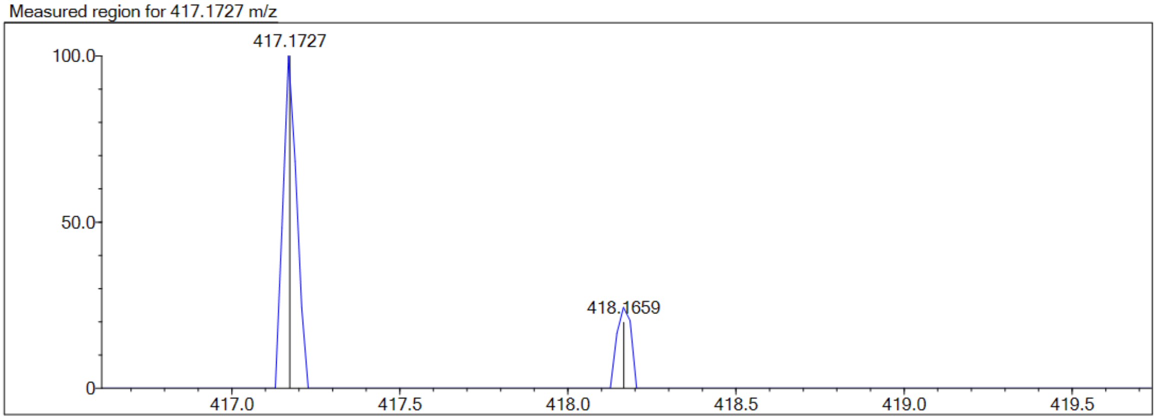

C27 H20 N4 O [M+H]+ : Predicted region for 417.1710 m/z

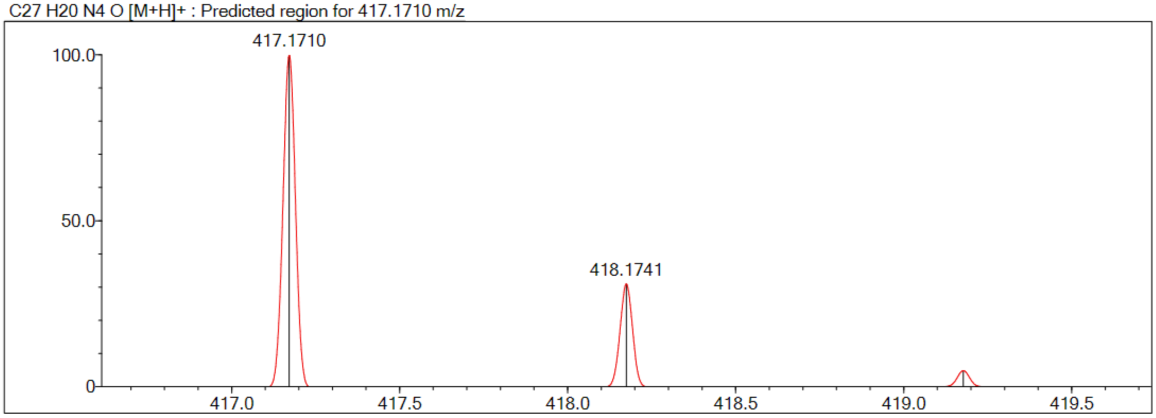

Rank	Score	Formula (M)	Ion	Meas. m/z	Pred. m/z	Df. (mDa)	Df. (ppm)	Iso	DBE
1	43.04	C27 H20 N4 O	[M+H]+	417.1727	417.1710	1.7	4.08	46.63	20.0

Figure 24. Mass spectrum of compound **3h**

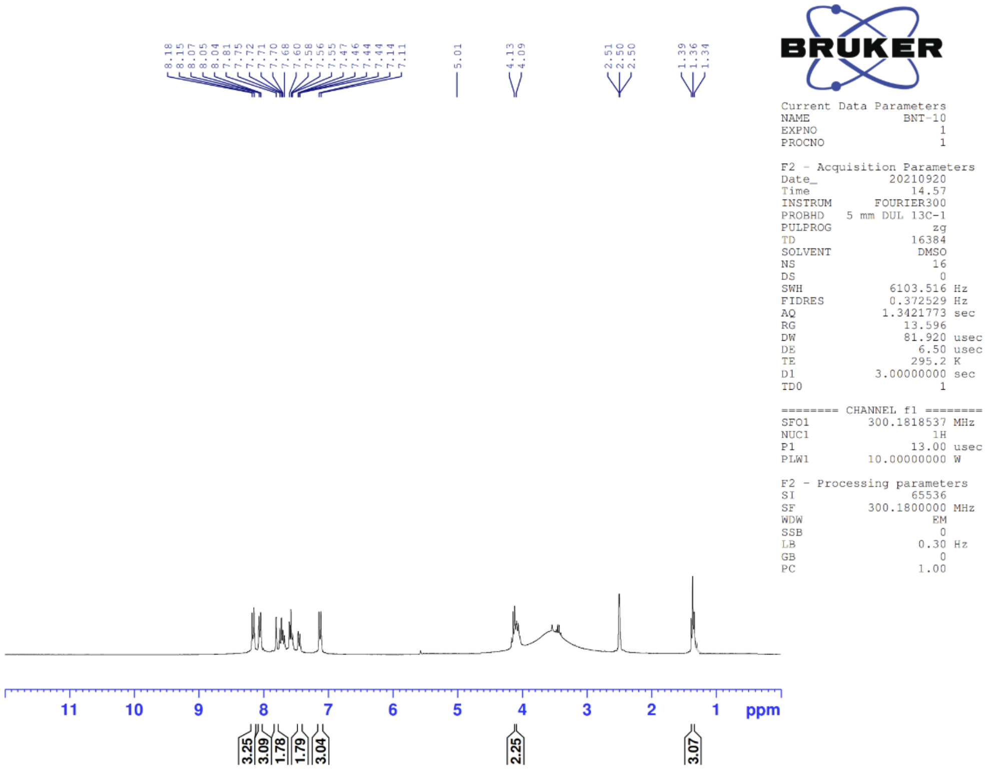

Figure 25. ^{1}H-NMR spectrum of compound **3i**

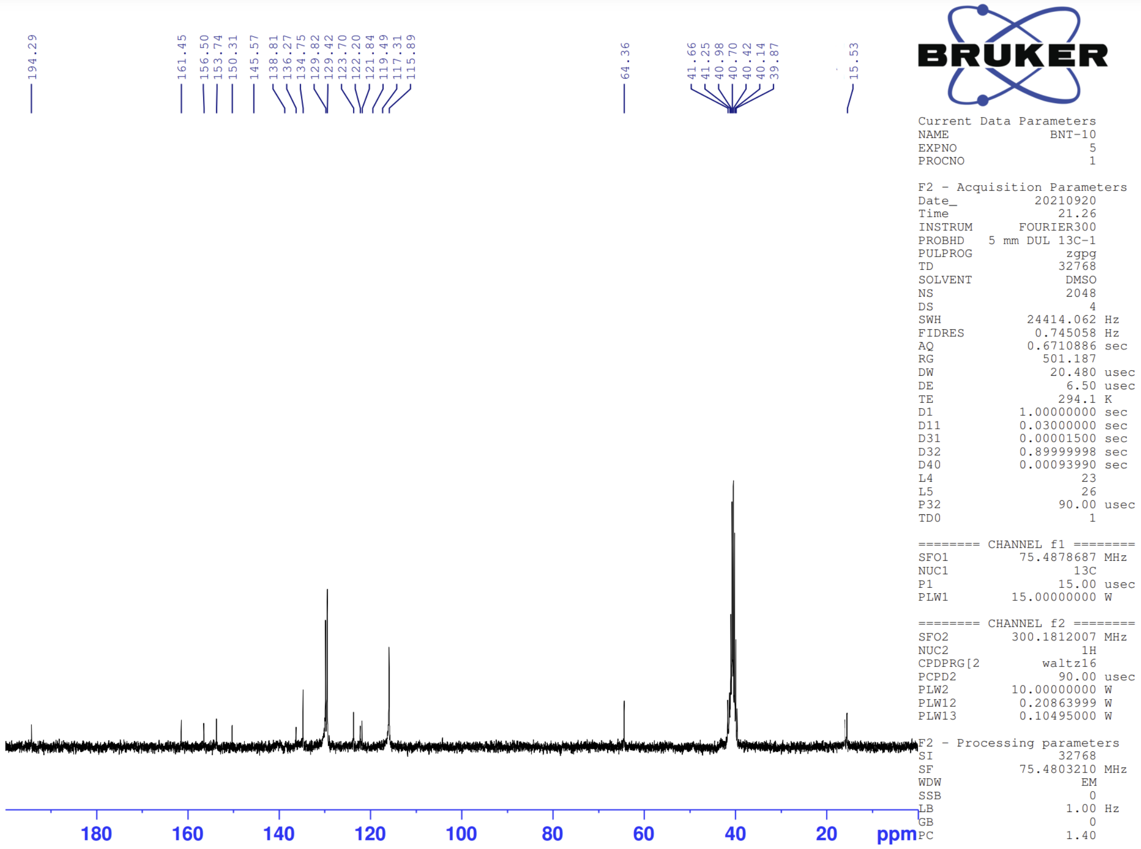

Figure 26. ^{13}C-NMR spectrum of compound **3i**

Formula Predictor Report - HB-9_59.lcd Page 1 of 1

Data File: C:\LabSolutions\Data\Analiz\uac\HB-9_59.lcd

Elmt	Val.	Min	Max	Elmt	Val.	Min	Max	Elmt	Val.	Min	Max	Elmt	Val.	Min	Max	Use Adduct
H	1	10	40	O	2	1	3	S	2	0	0	Ru	2	0	0	H
C	4	9	40	F	1	0	0	Cl	1	0	0	Pd	2	0	0	
N	3	4	6	P	3	0	0	Br	1	0	0	I	3	0	0	

Error Margin (ppm): 5
HC Ratio: unlimited
Max Isotopes: 3
MSn Iso RI (%): 10.00

DBE Range: 5.0 - 20.0
Apply N Rule: yes
Isotope RI (%): 1.00
MSn Logic Mode: AND

Electron Ions: both
Use MSn Info: yes
Isotope Res: 9000
Max Results: 150

Event#: 1 MS(E+) Ret. Time : 3.347 Scan# : 503

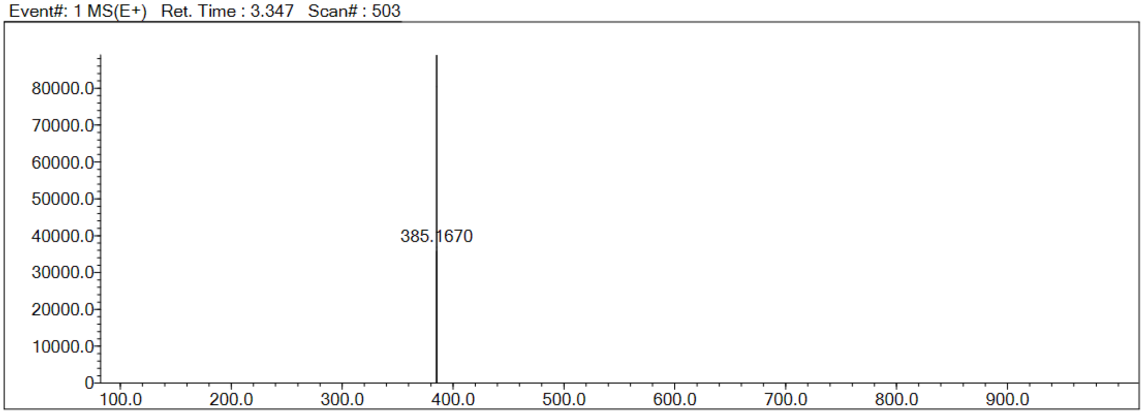

Measured region for 385.1670 m/z

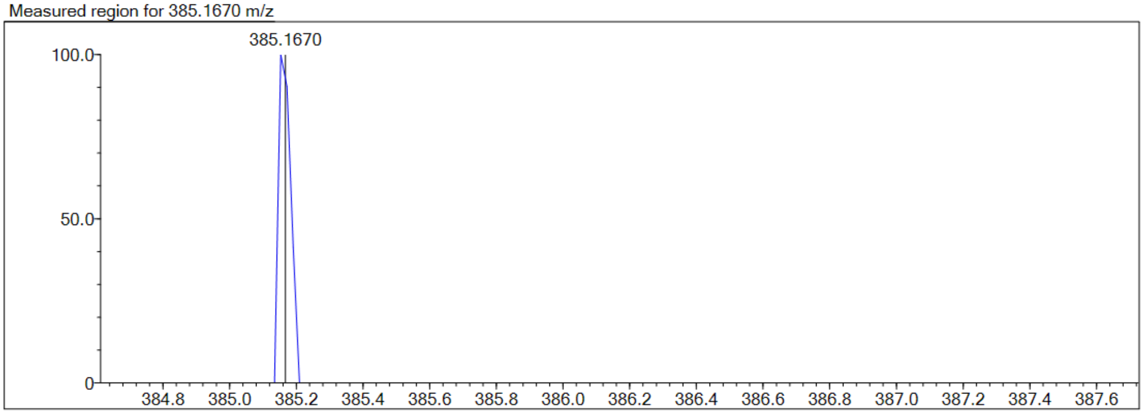

C23 H20 N4 O2 [M+H]+ : Predicted region for 385.1659 m/z

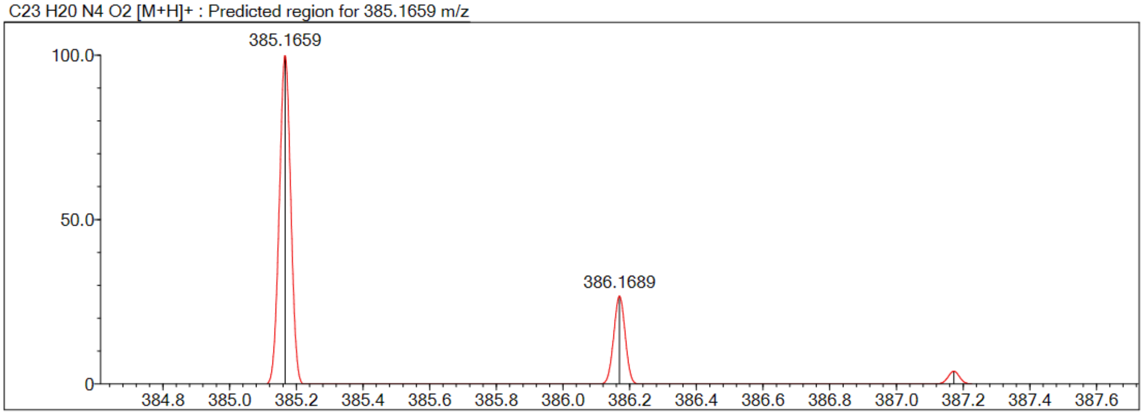

Rank	Score	Formula (M)	Ion	Meas. m/z	Pred. m/z	Df. (mDa)	Df. (ppm)	Iso	DBE
1	0.00	C23 H20 N4 O2	[M+H]+	385.1670	385.1659	1.1	2.86	0.00	16.0

Figure 27. Mass spectrum of compound **3i**

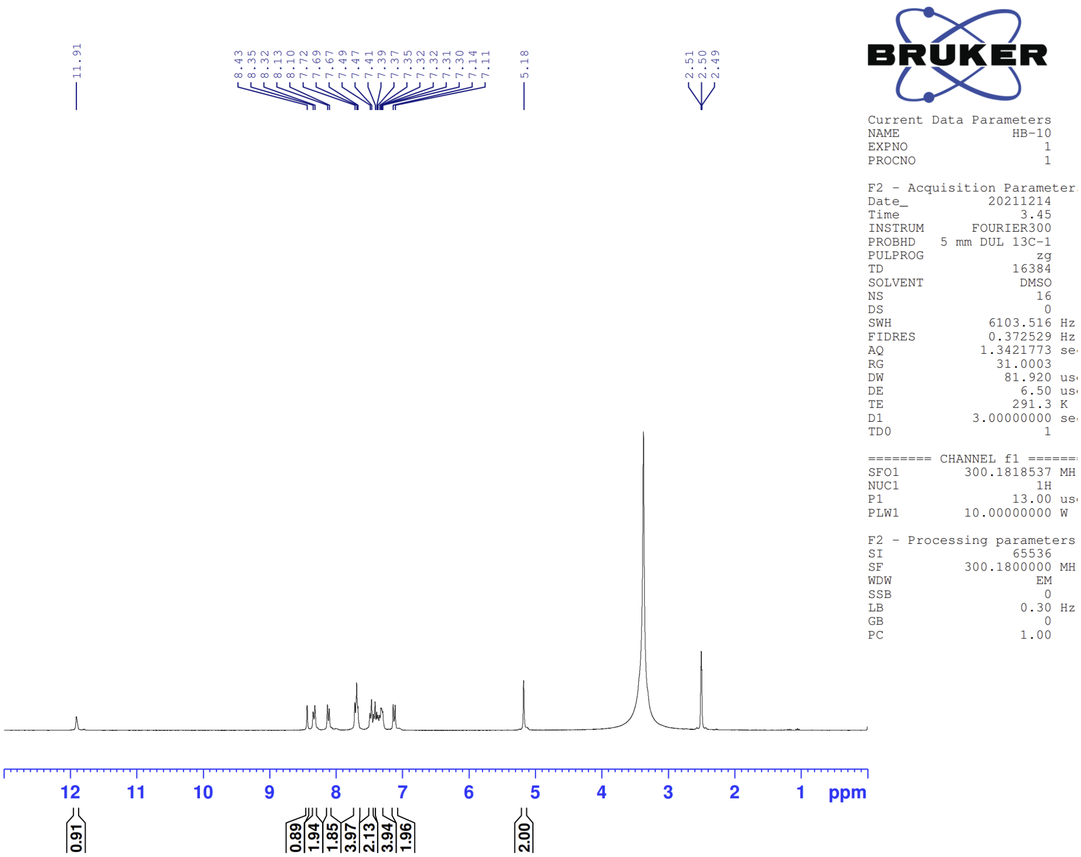

Figure 28. ^{1}H-NMR spectrum of compound **3j**

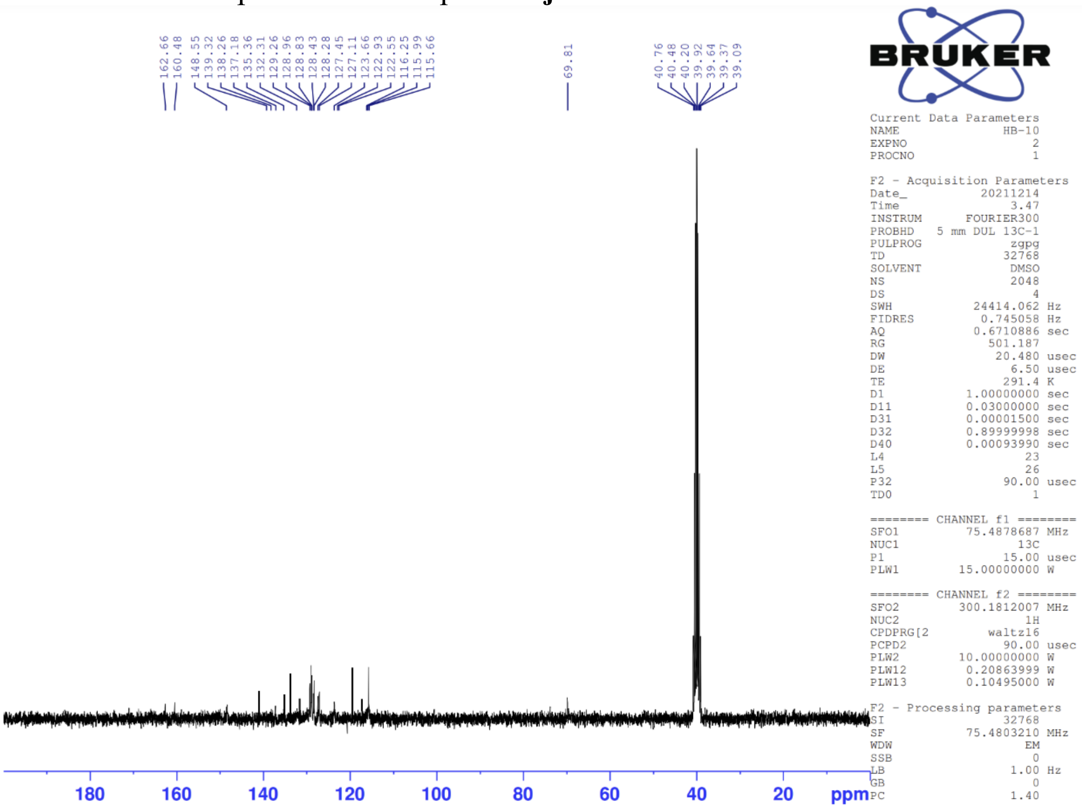

Figure 29. ^{13}C-NMR spectrum of compound **3j**

Formula Predictor Report - HB-10_60.lcd

Data File: C:\LabSolutions\Data\Analiz\uac\HB-10_60.lcd

Elmt	Val.	Min	Max	Elmt	Val.	Min	Max	Elmt	Val.	Min	Max	Elmt	Val.	Min	Max	Use Adduct
H	1	10	40	O	2	1	3	S	2	0	0	Ru	2	0	0	H
C	4	9	40	F	1	0	0	Cl	1	0	0	Pd	2	0	0	
N	3	4	6	P	3	0	0	Br	1	0	0	I	3	0	0	

Error Margin (ppm): 5
HC Ratio: unlimited
Max Isotopes: 3
MSn Iso RI (%): 10.00

DBE Range: 5.0 - 20.0
Apply N Rule: yes
Isotope RI (%): 1.00
MSn Logic Mode: AND

Electron Ions: both
Use MSn Info: yes
Isotope Res: 9000
Max Results: 150

Event#: 1 MS(E+) Ret. Time : 3.960 Scan# : 595

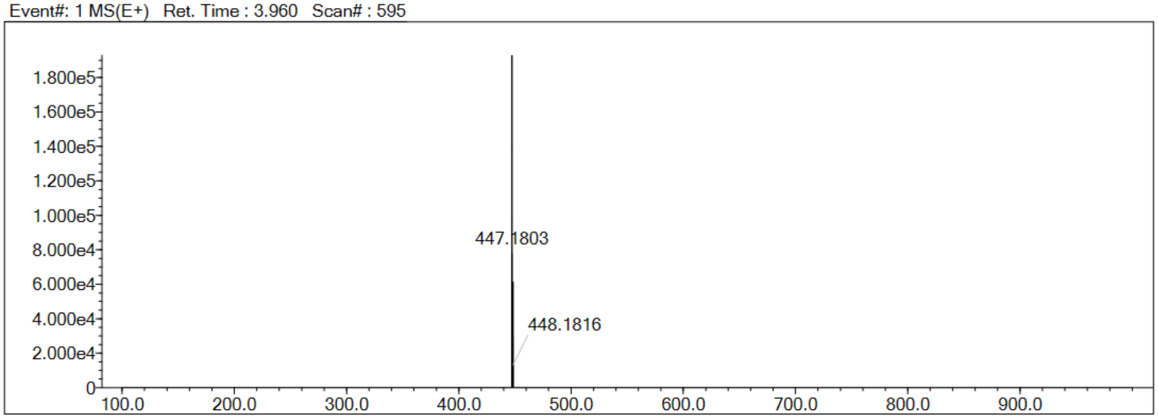

Measured region for 447.1803 m/z

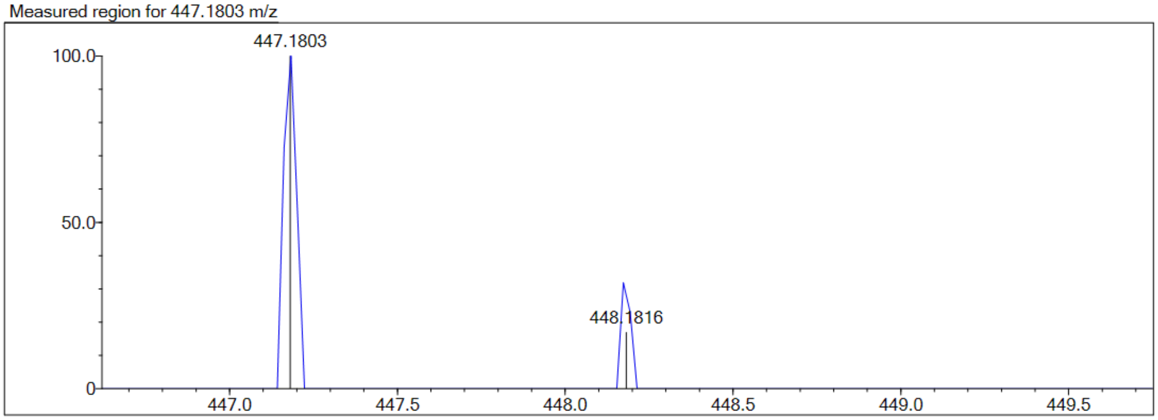

C28 H22 N4 O2 [M+H]+ : Predicted region for 447.1816 m/z

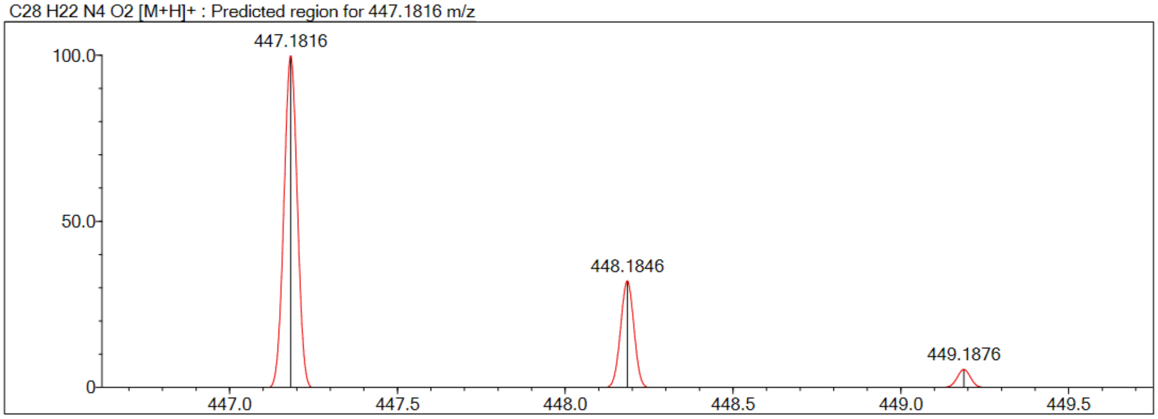

Rank	Score	Formula (M)	Ion	Meas. m/z	Pred. m/z	Df. (mDa)	Df. (ppm)	Iso	DBE
1	64.42	C28 H22 N4 O2	[M+H]+	447.1803	447.1816	-1.3	-2.91	67.65	20.0

Figure 30. Mass spectrum of compound **3j**

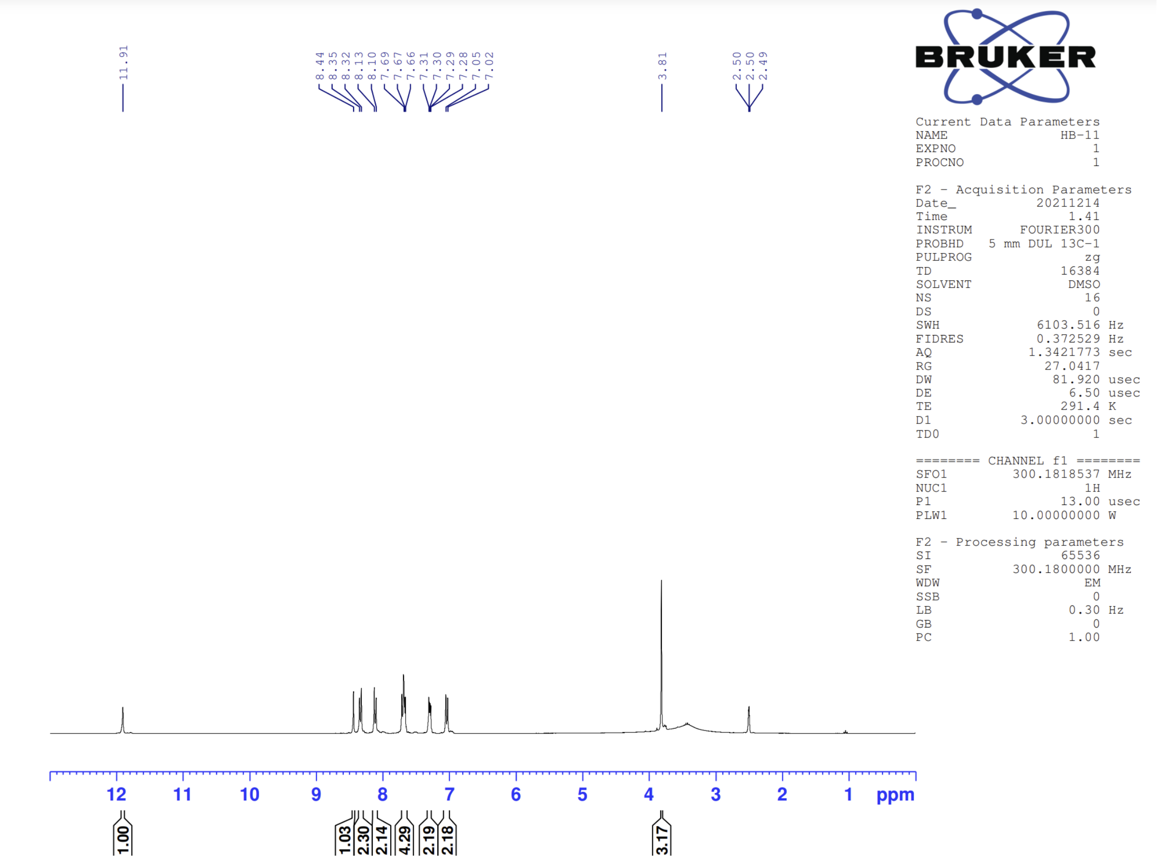

Figure 31. ^{1}H-NMR spectrum of compound **3k**

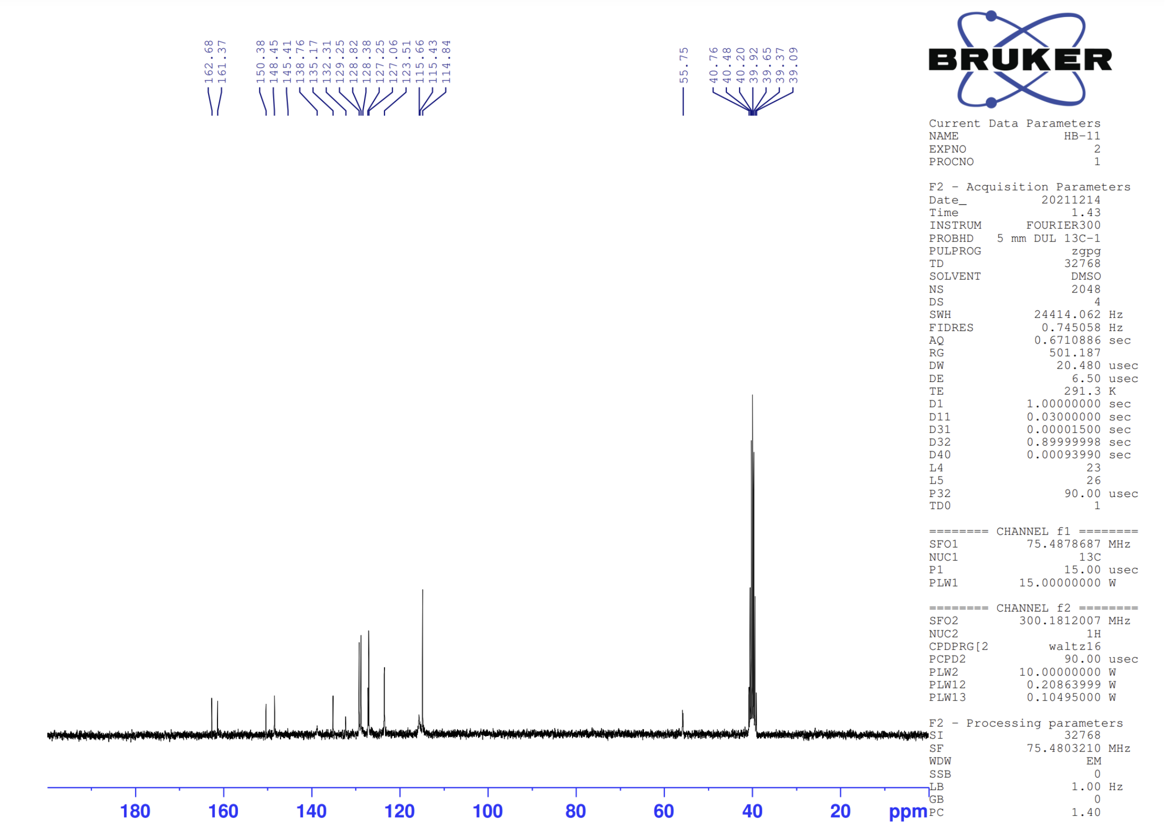

Figure 32. ^{13}C-NMR spectrum of compound **3k**

Formula Predictor Report - HB-11_61.lcd Page 1 of 1

Data File: C:\LabSolutions\Data\Analiz\uac\HB-11_61.lcd

Elmt	Val.	Min	Max	Elmt	Val.	Min	Max	Elmt	Val.	Min	Max	Elmt	Val.	Min	Max	Use Adduct
H	1	10	40	O	2	1	3	S	2	0	0	Ru	2	0	0	H
C	4	9	40	F	1	0	0	Cl	1	0	0	Pd	2	0	0	
N	3	4	6	P	3	0	0	Br	1	0	0	I	3	0	0	

Error Margin (ppm):	5	DBE Range:	5.0 - 20.0	Electron Ions:	both
HC Ratio:	unlimited	Apply N Rule:	yes	Use MSn Info:	yes
Max Isotopes:	3	Isotope RI (%):	1.00	Isotope Res:	9000
MSn Iso RI (%):	10.00	MSn Logic Mode:	AND	Max Results:	150

Event#: 1 MS(E+) Ret. Time : 3.013 Scan# : 453

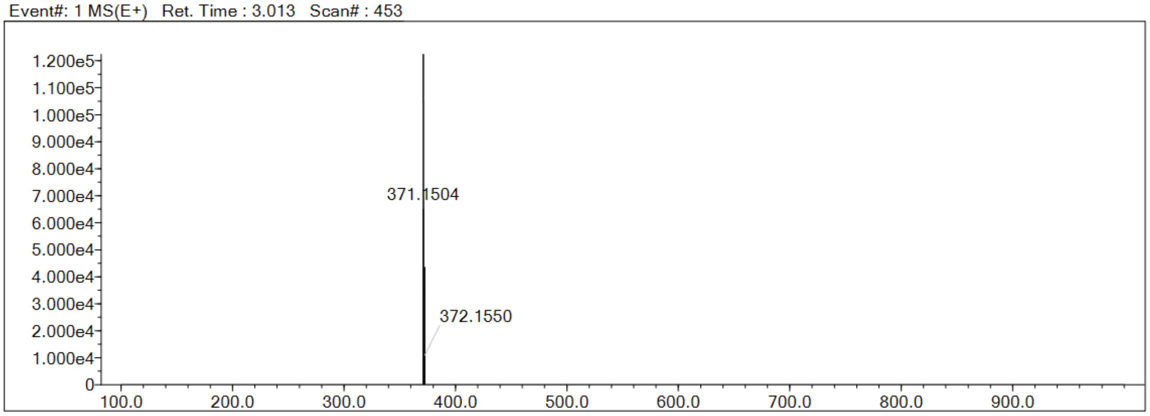

Measured region for 371.1504 m/z

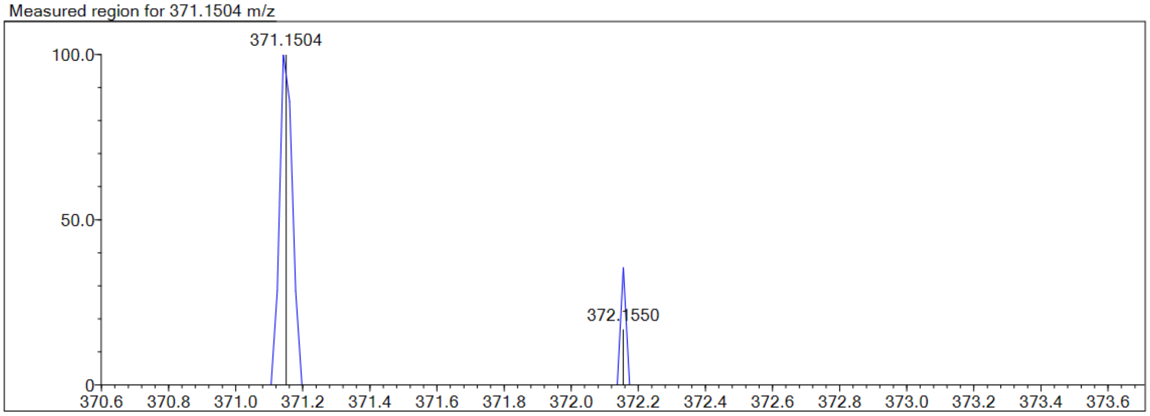

C22 H18 N4 O2 [M+H]+ : Predicted region for 371.1503 m/z

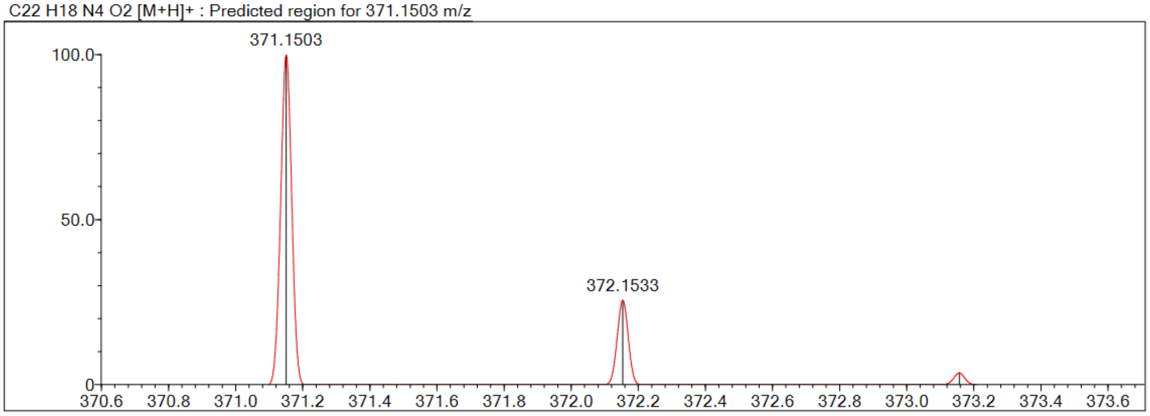

Rank	Score	Formula (M)	Ion	Meas. m/z	Pred. m/z	Df. (mDa)	Df. (ppm)	Iso	DBE
1	68.87	C22 H18 N4 O2	[M+H]+	371.1504	371.1503	0.1	0.27	68.87	16.0

Figure 33. Mass spectrum of compound **3k**

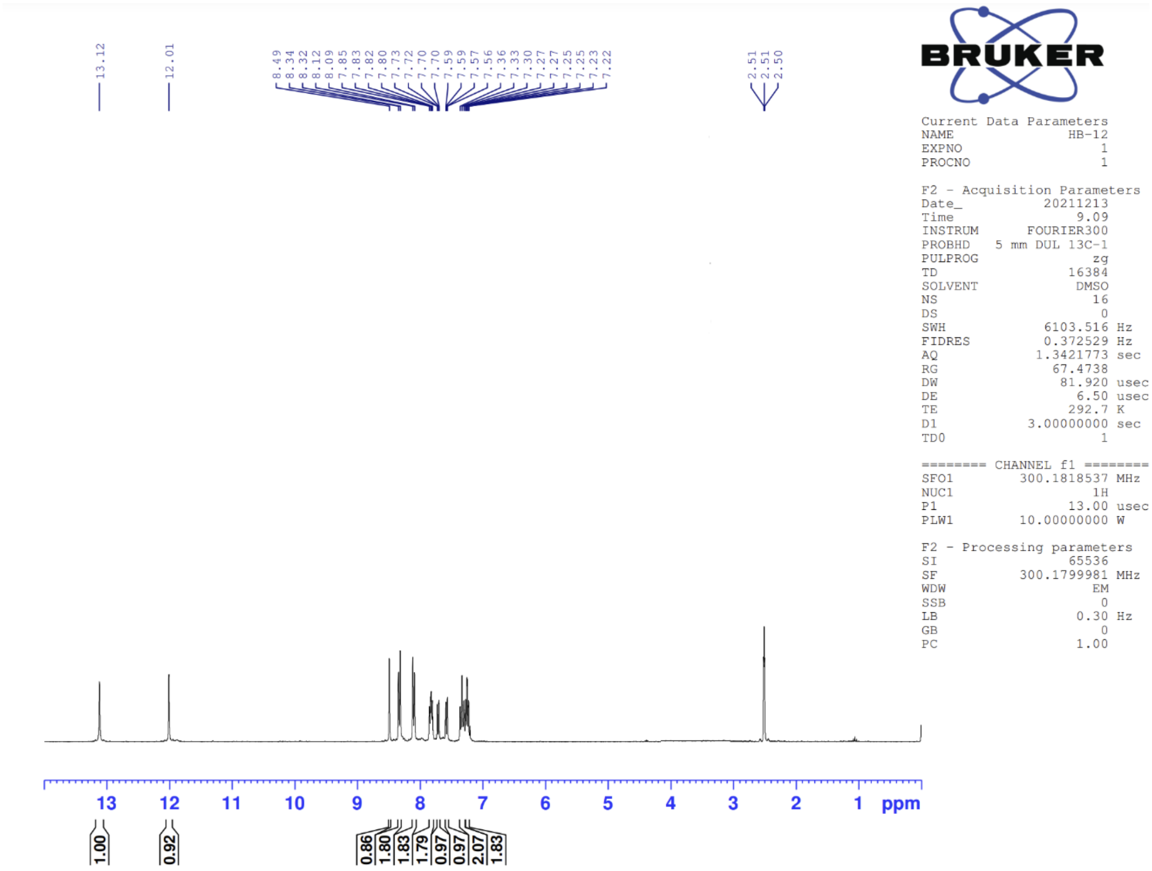

Figure 34. ^{1}H-NMR spectrum of compound **3l**

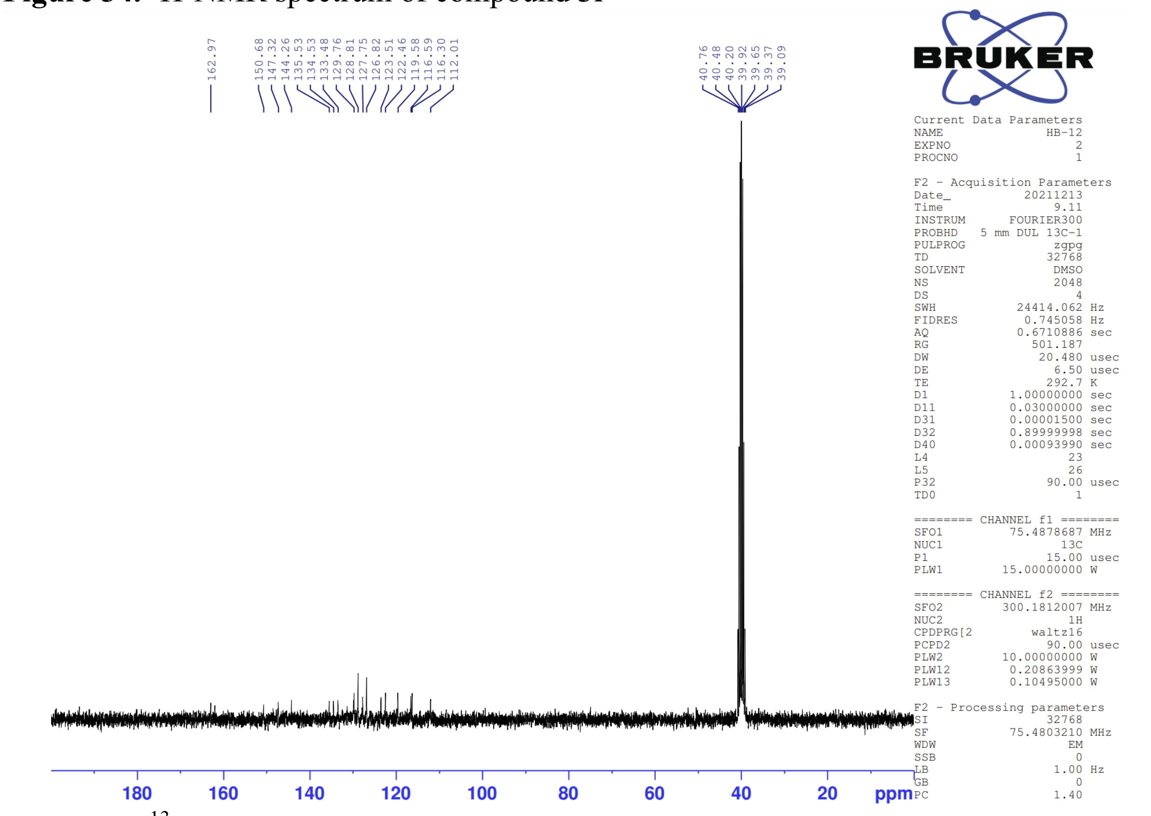

Figure 35. ^{13}C-NMR spectrum of compound **3l**

Formula Predictor Report - HB-12_62.lcd Page 1 of 1

Data File: C:\LabSolutions\Data\Analiz\uac\HB-12_62.lcd

Elmt	Val.	Min	Max	Elmt	Val.	Min	Max	Elmt	Val.	Min	Max	Elmt	Val.	Min	Max	Use Adduct
H	1	10	40	O	2	1	3	S	2	0	0	Ru	2	0	0	H
C	4	9	40	F	1	1	1	Cl	1	0	0	Pd	2	0	0	
N	3	4	6	P	3	0	0	Br	1	0	0	I	3	0	0	

Error Margin (ppm): 5
HC Ratio: unlimited
Max Isotopes: 3
MSn Iso RI (%): 10.00

DBE Range: 5.0 - 20.0
Apply N Rule: yes
Isotope RI (%): 1.00
MSn Logic Mode: AND

Electron Ions: both
Use MSn Info: yes
Isotope Res: 9000
Max Results: 150

Event#: 1 MS(E+) Ret. Time : 3.253 Scan# : 489

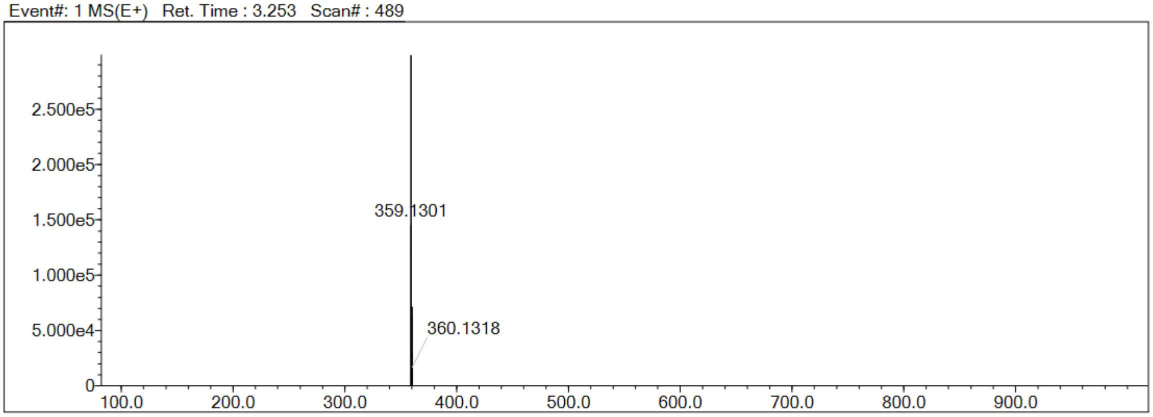

Measured region for 359.1301 m/z

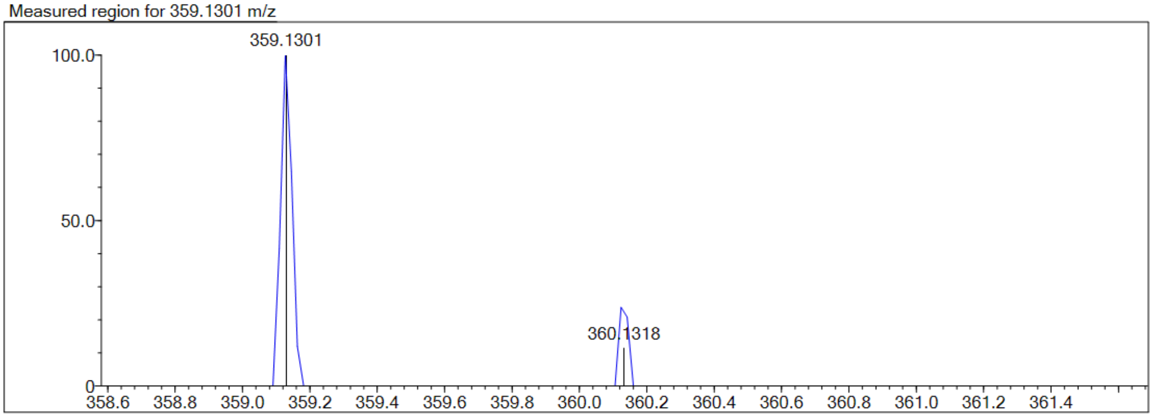

C21 H15 N4 O F [M+H]+ : Predicted region for 359.1303 m/z

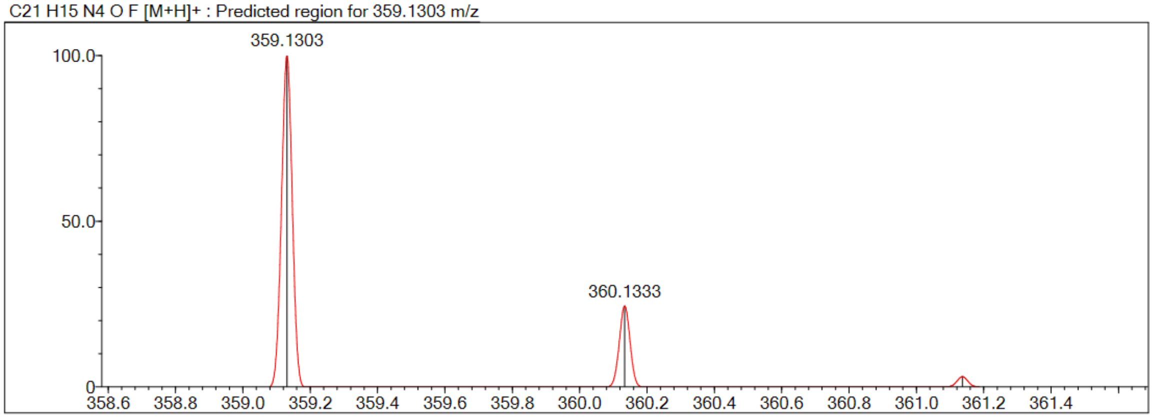

Rank	Score	Formula (M)	Ion	Meas. m/z	Pred. m/z	Df. (mDa)	Df. (ppm)	Iso	DBE
1	73.27	C21 H15 N4 O F	[M+H]+	359.1301	359.1303	-0.2	-0.56	73.27	16.0

Figure 36. Mass spectrum of compound **3l**

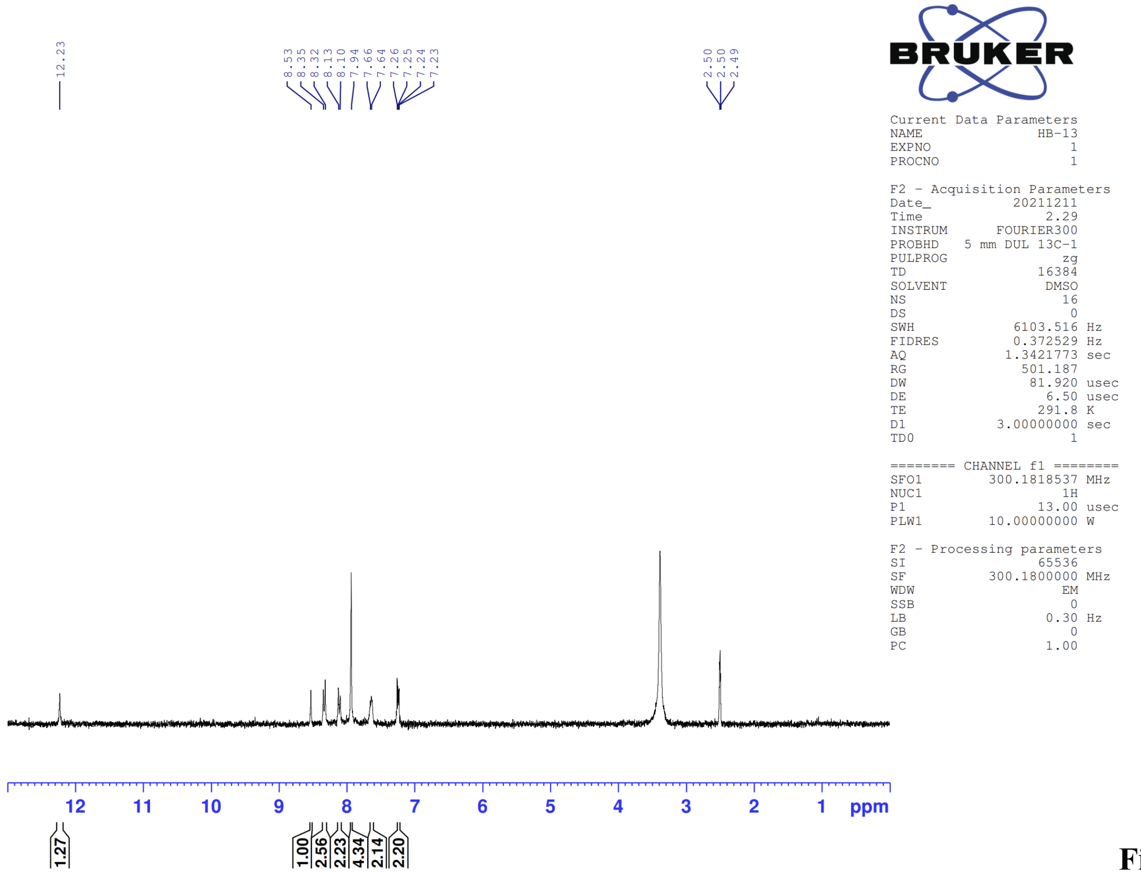

Figure 37. ^{1}H-NMR spectrum of compound **3m**

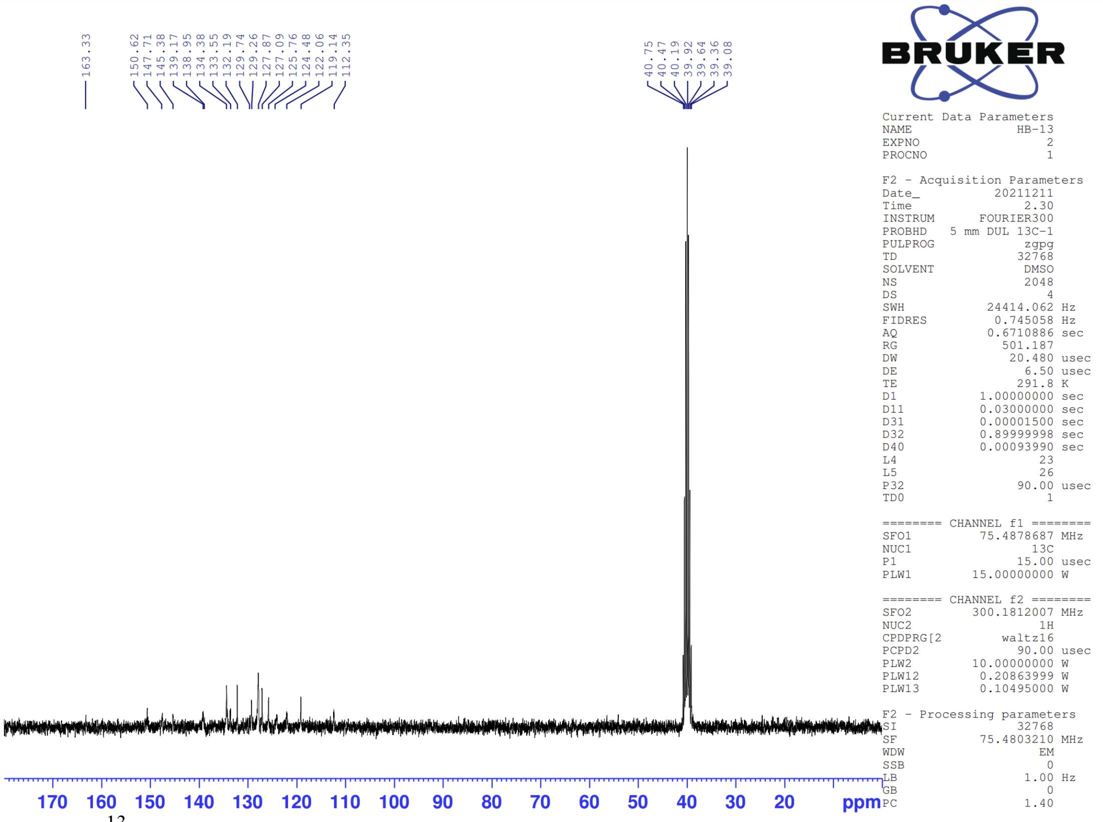

Figure 38. ^{13}C-NMR spectrum of compound **3m**

Formula Predictor Report - HB-13_63.lcd Page 1 of 1

Data File: C:\LabSolutions\Data\Analiz\uac\HB-13_63.lcd

Elmt	Val.	Min	Max	Elmt	Val.	Min	Max	Elmt	Val.	Min	Max	Elmt	Val.	Min	Max	Use Adduct
H	1	10	40	O	2	1	3	S	2	0	0	Ru	2	0	0	H
C	4	9	40	F	1	0	0	Cl	1	0	0	Pd	2	0	0	
N	3	4	6	P	3	0	0	Br	1	0	0	I	3	0	0	

Error Margin (ppm): 5
HC Ratio: unlimited
Max Isotopes: 3
MSn Iso RI (%): 10.00

DBE Range: 5.0 - 20.0
Apply N Rule: yes
Isotope RI (%): 1.00
MSn Logic Mode: AND

Electron Ions: both
Use MSn Info: yes
Isotope Res: 9000
Max Results: 150

Event#: 1 MS(E+) Ret. Time : 3.200 Scan# : 481

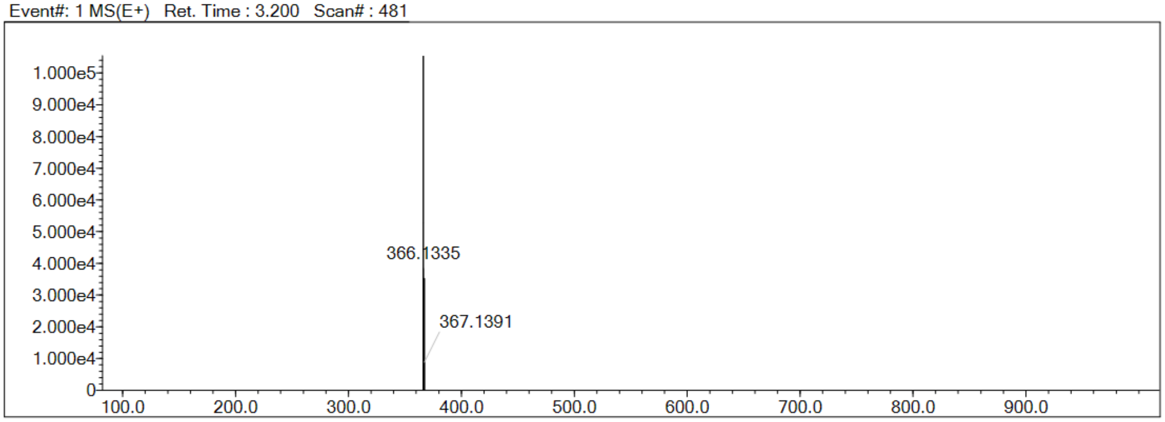

Measured region for 366.1335 m/z

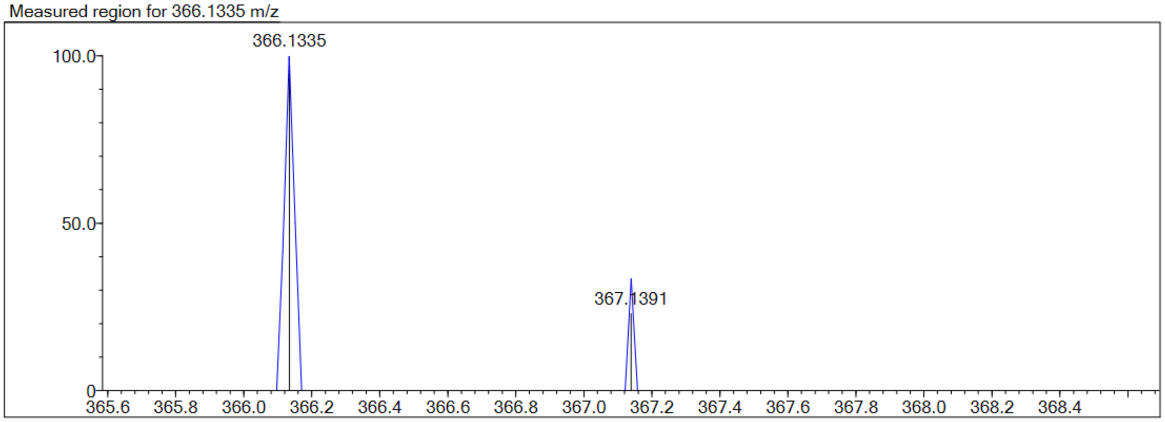

C22 H15 N5 O [M+H]+ : Predicted region for 366.1349 m/z

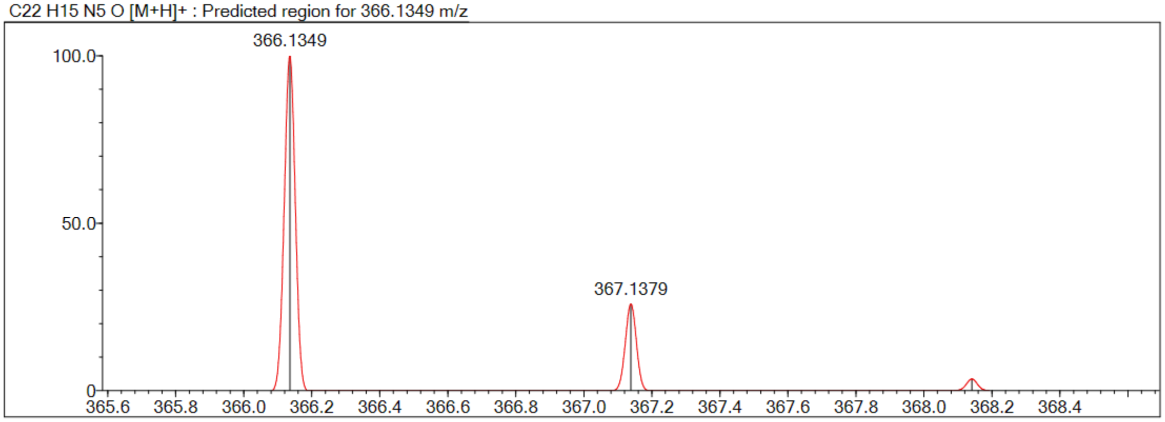

Rank	Score	Formula (M)	Ion	Meas. m/z	Pred. m/z	Df. (mDa)	Df. (ppm)	Iso	DBE
1	66.88	C22 H15 N5 O	[M+H]+	366.1335	366.1349	-1.4	-3.82	71.96	18.0

Figure 39. Mass spectrum of compound **3m**

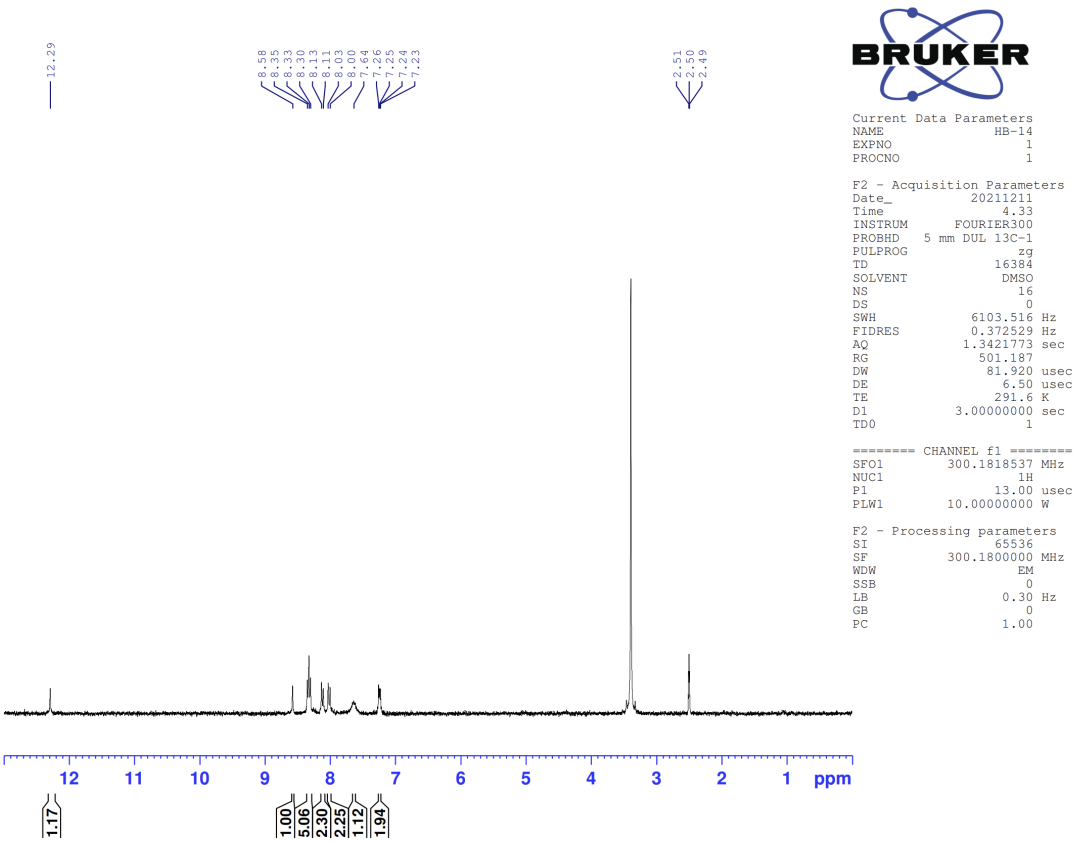

Figure 40. ^{1}H-NMR spectrum of compound **3n**

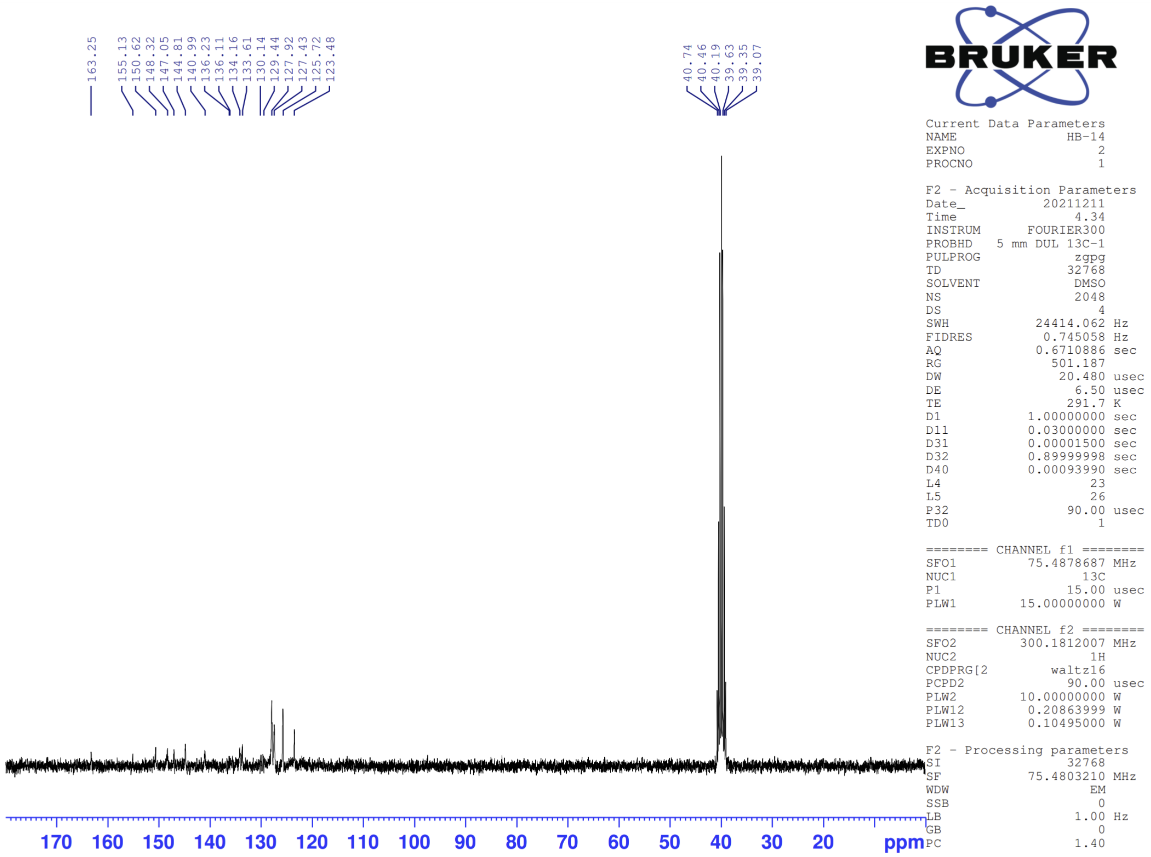

Figure 41. ^{13}C-NMR spectrum of compound **3n**

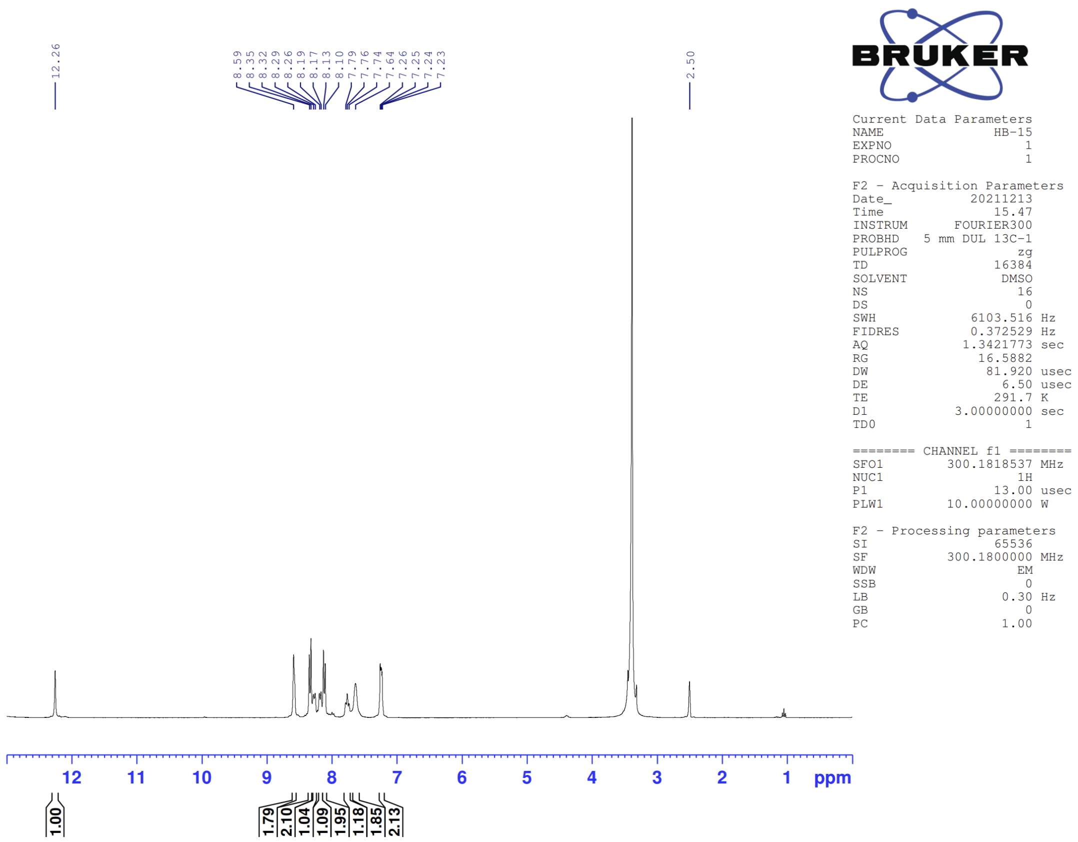

Figure 42. ^{1}H-NMR spectrum of compound **3o**

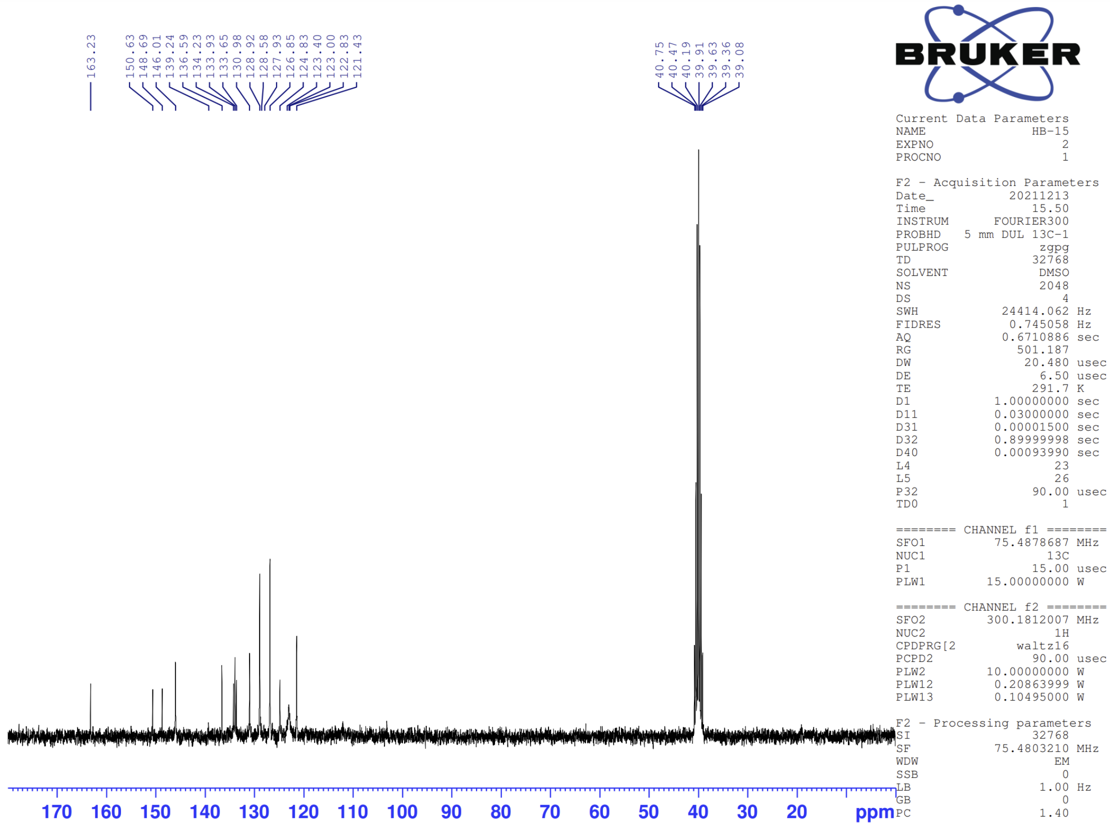

Figure 43. ^{13}C-NMR spectrum of compound **3o**

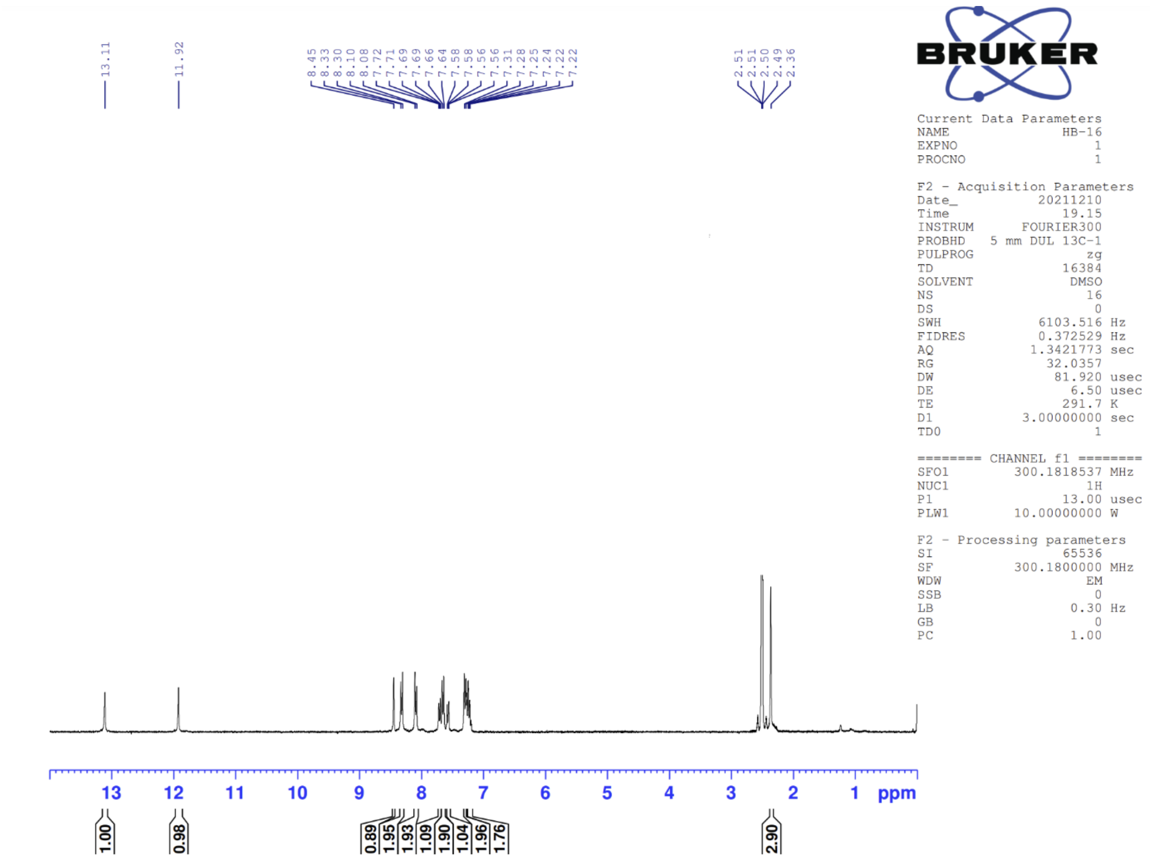

Figure 44. ^{1}H-NMR spectrum of compound **3p**

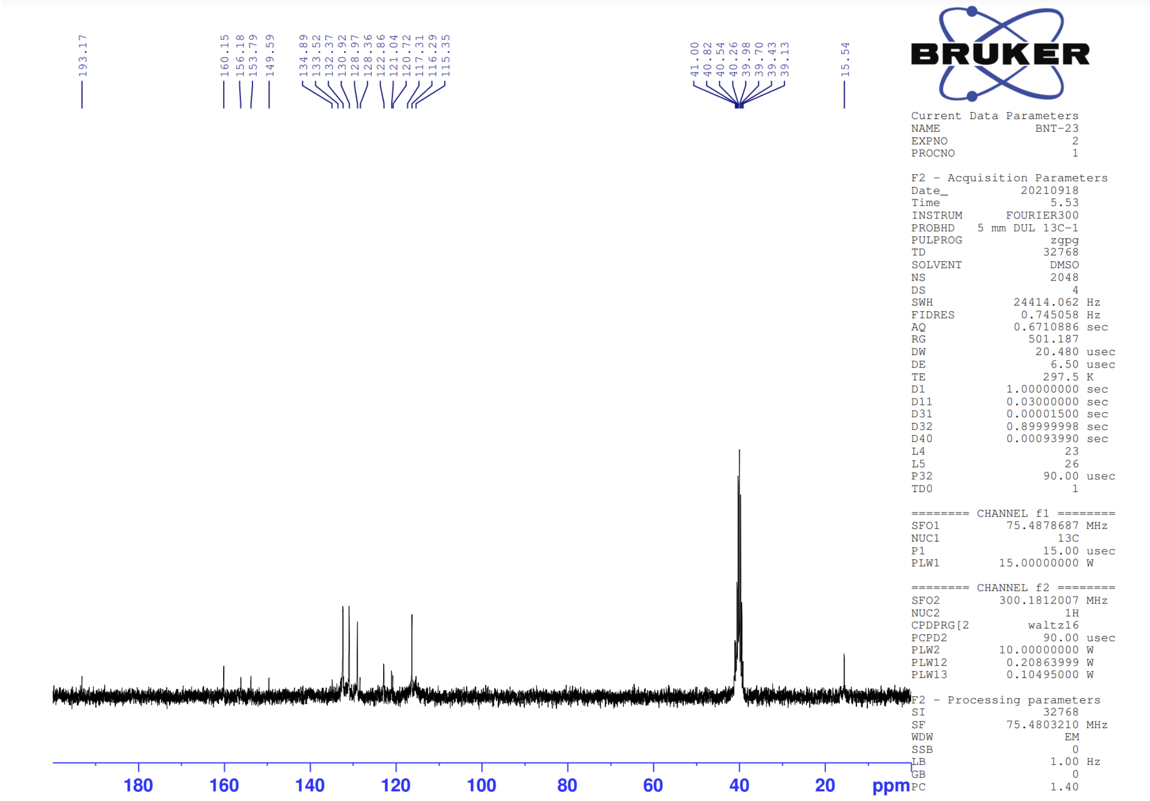

Figure 45. ^{13}C-NMR spectrum of compound **3p**

Formula Predictor Report - HB-16_66.lcd Page 1 of 1

Data File: C:\LabSolutions\Data\Analiz\uac\HB-16_66.lcd

Elmt	Val.	Min	Max	Elmt	Val.	Min	Max	Elmt	Val.	Min	Max	Elmt	Val.	Min	Max	Use Adduct
H	1	10	40	O	2	1	3	S	2	0	0	Ru	2	0	0	H
C	4	9	40	F	1	0	0	Cl	1	0	0	Pd	2	0	0	
N	3	4	6	P	3	0	0	Br	1	0	0	I	3	0	0	

Error Margin (ppm): 5
HC Ratio: unlimited
Max Isotopes: 3
MSn Iso RI (%): 10.00

DBE Range: 5.0 - 20.0
Apply N Rule: yes
Isotope RI (%): 1.00
MSn Logic Mode: AND

Electron Ions: both
Use MSn Info: yes
Isotope Res: 9000
Max Results: 150

Event#: 1 MS(E+) Ret. Time : 3.360 Scan# : 505

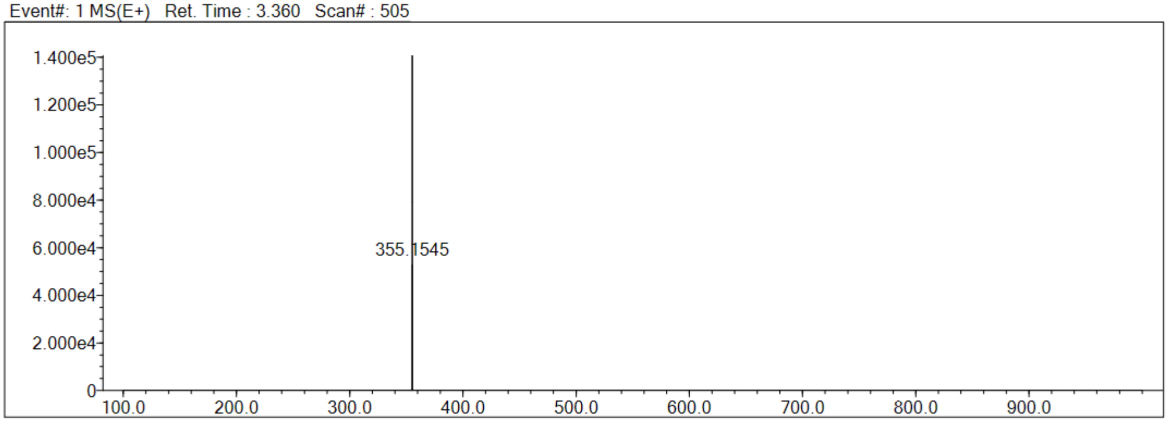

Measured region for 355.1545 m/z

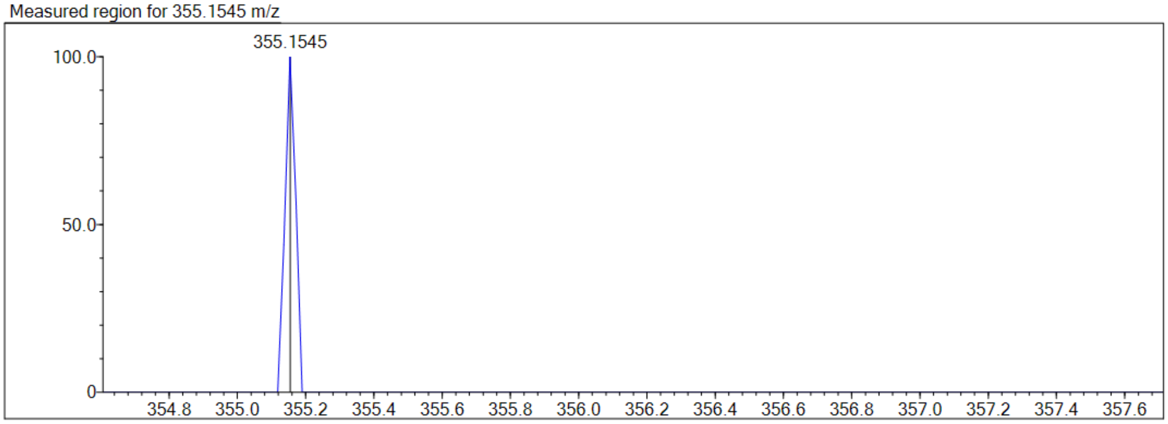

C22 H18 N4 O [M+H]+ : Predicted region for 355.1553 m/z

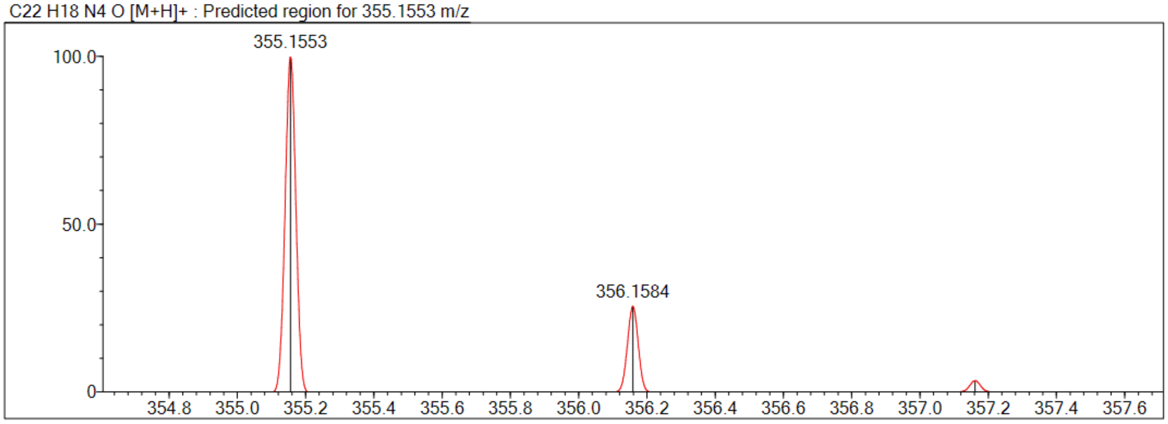

Rank	Score	Formula (M)	Ion	Meas. m/z	Pred. m/z	Df. (mDa)	Df. (ppm)	Iso	DBE
1	0.00	C22 H18 N4 O	[M+H]+	355.1545	355.1553	-0.8	-2.25	0.00	16.0

Figure 46. Mass spectrum of compound **3p**

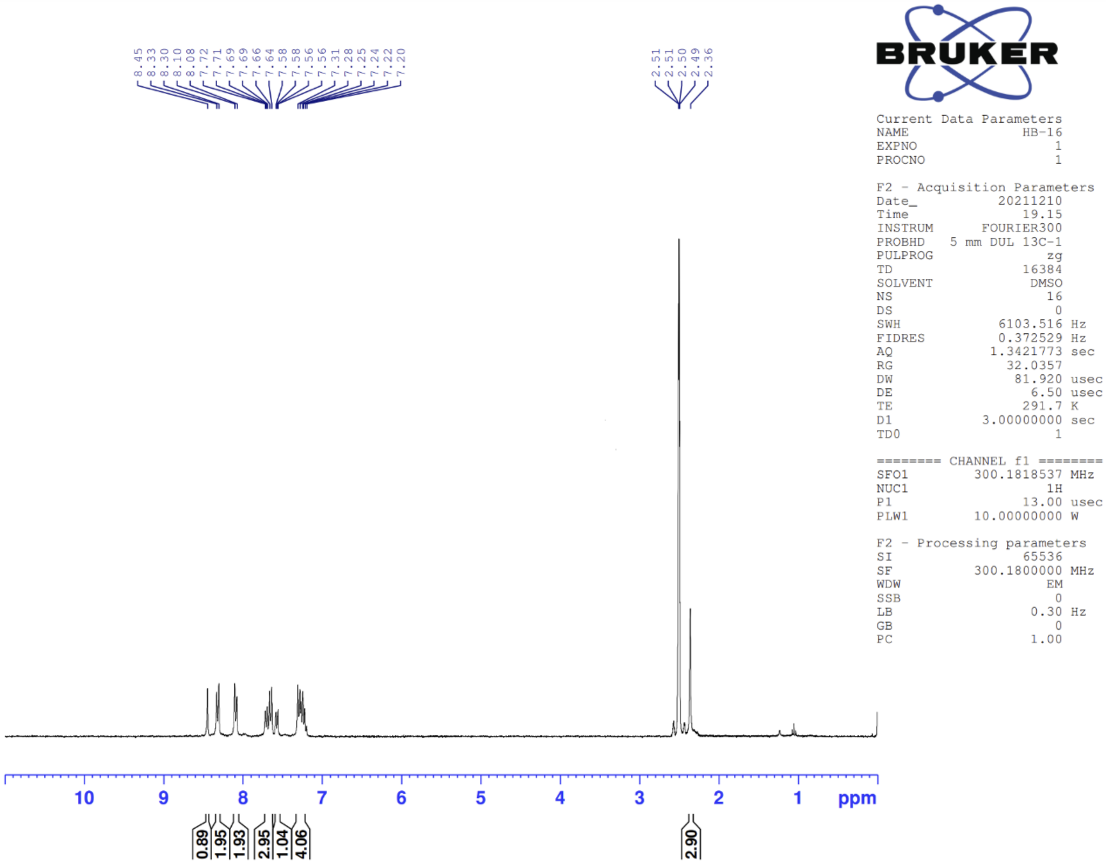

Figure 47. ^{1}H-NMR spectrum of compound **3r**

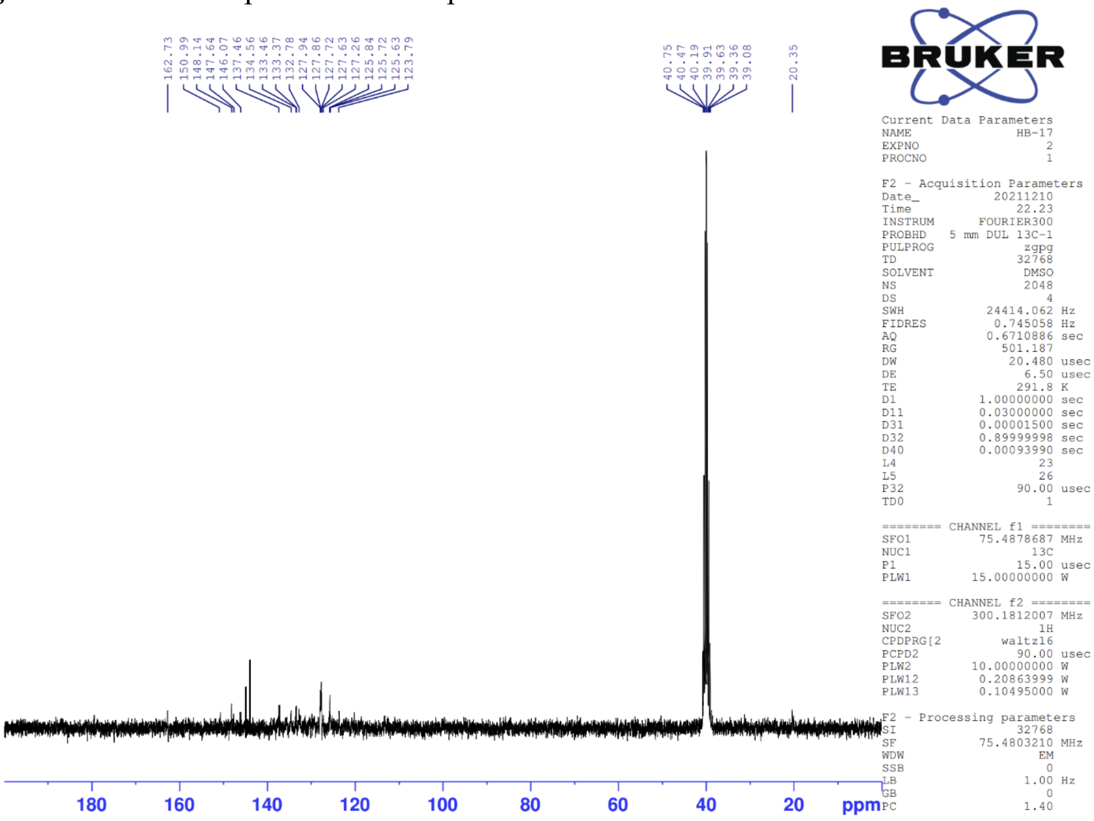

Figure 48. ^{13}C-NMR spectrum of compound **3r**

Formula Predictor Report - HB-17_67.lcd Page 1 of 1

Data File: C:\LabSolutions\Data\Analiz\uac\HB-17_67.lcd

Elmt	Val.	Min	Max	Elmt	Val.	Min	Max	Elmt	Val.	Min	Max	Elmt	Val.	Min	Max	Use Adduct
H	1	10	40	O	2	1	3	S	2	0	0	Ru	2	0	0	H
C	4	9	40	F	1	0	0	Cl	1	0	0	Pd	2	0	0	
N	3	4	6	P	3	0	0	Br	1	0	0	I	3	0	0	

Error Margin (ppm):	5	DBE Range:	5.0 - 20.0	Electron Ions:	both
HC Ratio:	unlimited	Apply N Rule:	yes	Use MSn Info:	yes
Max Isotopes:	3	Isotope RI (%):	1.00	Isotope Res:	9000
MSn Iso RI (%):	10.00	MSn Logic Mode:	AND	Max Results:	150

Event#: 1 MS(E+) Ret. Time : 3.800 Scan# : 571

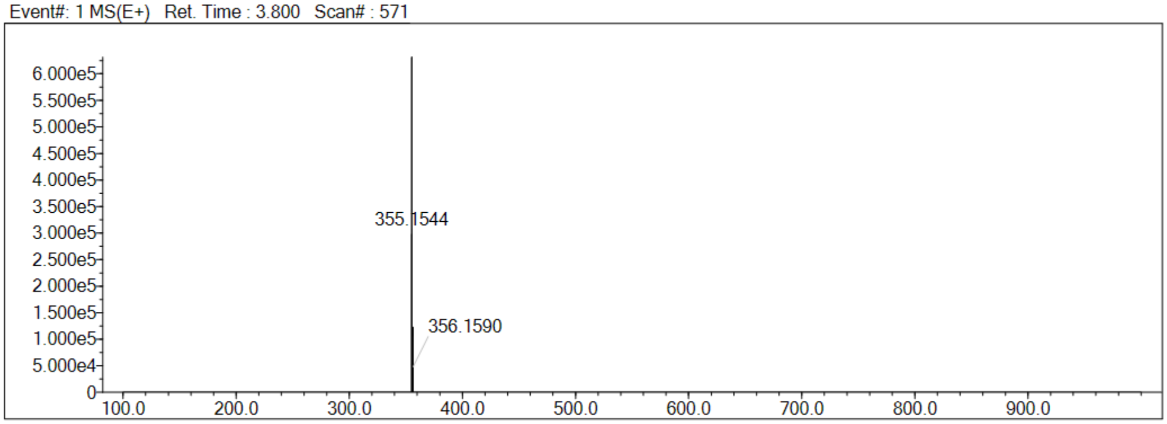

Measured region for 355.1544 m/z

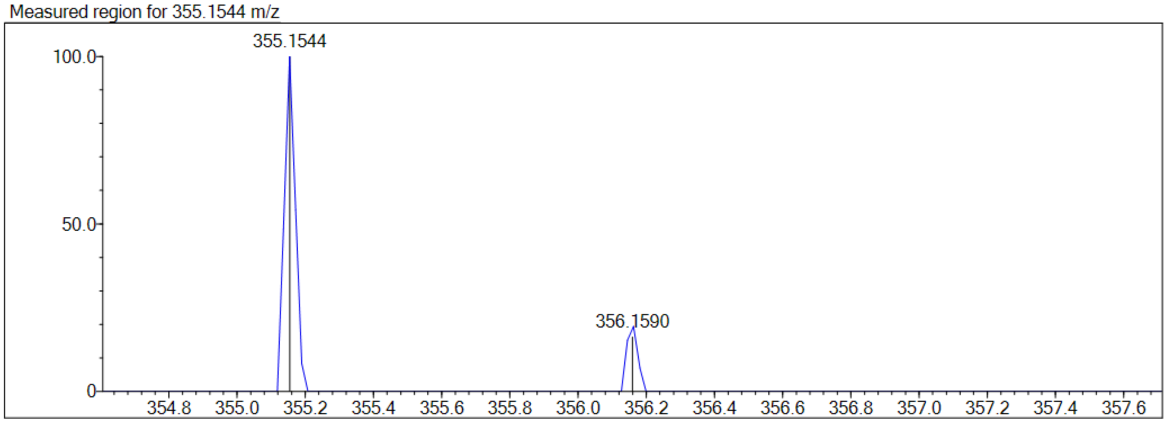

C22 H18 N4 O [M+H]+ : Predicted region for 355.1553 m/z

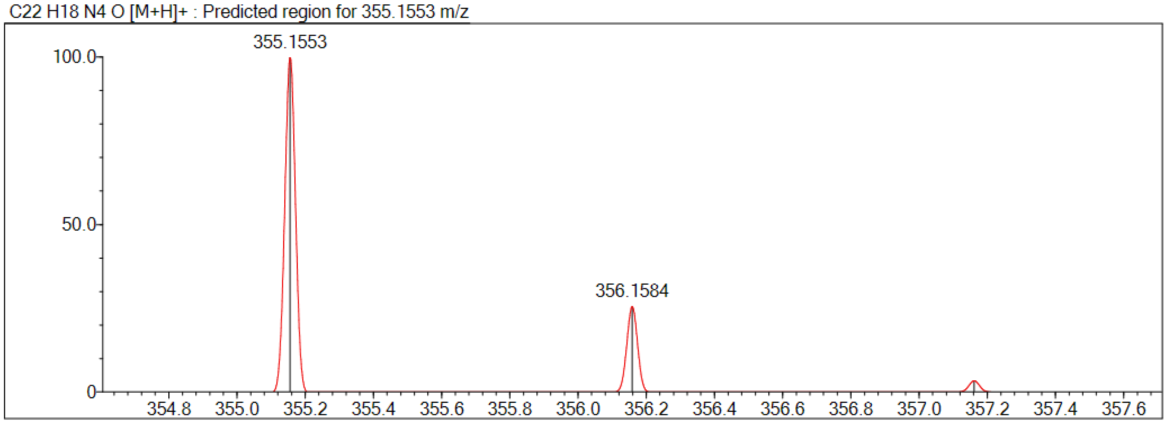

Rank	Score	Formula (M)	Ion	Meas. m/z	Pred. m/z	Df. (mDa)	Df. (ppm)	Iso	DBE
1	68.01	C22 H18 N4 O	[M+H]+	355.1544	355.1553	-0.9	-2.53	70.72	16.0

Figure 49. Mass spectrum of compound **3r**
